# Novel psoralen derivatives as anti-breast cancer agents and their light-activated cytotoxicity against HER2 positive breast cancer cells

**DOI:** 10.1038/s41598-022-17625-x

**Published:** 2022-08-05

**Authors:** Chiphada Aekrungrueangkit, Sirilak Wangngae, Anyanee Kamkaew, Ruchuta Ardkhean, Sanit Thongnest, Jutatip Boonsombat, Somsak Ruchirawat, Tanatorn Khotavivattana

**Affiliations:** 1 Center of Excellence in Natural Products Chemistry, Department of Chemistry, Faculty of Science, Chulalongkorn University, Bangkok, 10330 Thailand; 2 School of Chemistry, Institute of Science, Suranaree University of Technology, Nakhon Ratchasima, 30000 Thailand; 3 Princess Srisavangavadhana College of Medicine, Chulabhorn Royal Academy, Bangkok, 10210 Thailand; 4 Chulabhorn Research Institute, Bangkok, 10210 Thailand; 5 Center of Excellence on Environmental Health and Toxicology (EHT), OPS, MHESI, Bangkok, Thailand; 6 Program in Chemical Sciences, Chulabhorn Graduate Institute, Chulabhorn Royal Academy, Bangkok, 10210 Thailand

**Keywords:** Medicinal chemistry, Drug discovery and development, Drug discovery and development

## Abstract

Psoralen derivatives are well known for their unique phototoxicity and also exhibits promising anti-breast cancer activity both in the presence and the absence of UVA irradiation. However, the structure–activity relationship on this scaffold remains lacking. Herein, a series of psoralen derivatives with various C-5 substituents were synthesized and evaluated for their in vitro dark and light-activated cytotoxicity against three breast cancer cell lines: MDA-MB-231, T47-D, and SK-BR-3. The type of substituents dramatically impacted the activity, with the 4-bromobenzyl amide derivative (**3c**) exhibiting the highest dark cytotoxicity against T47-D (IC_50_ = 10.14 µM), with the activity comparable to those of the reference drugs (doxorubicin, 1.46 µM; tamoxifen citrate, 20.86 µM; lapatinib 9.78 µM). On the other hand, the furanylamide **3g** exhibits the highest phototoxicity against SK-BR-3 cells with the IC_50_ of 2.71 µM, which is almost tenfold increase compared to the parent compound, methoxsalen. Moreover, these derivatives showed exceptional selectivity towards HER2+ (SK-BR-3) over the HER2− (MDA-MB-231) breast cancer cell lines, which correlates well with the results from the molecular docking study, revealing that **3g** formed favorable interactions within the active site of the HER2. Additionally, the cell morphology of SK-BR-3 cells suggested that the significant phototoxicity was related to induction of cell apoptosis. Most of the synthesized psoralen derivatives possess acceptable physicochemical properties and are suitable for being further developed as a novel anti-breast cancer agent in the future.

## Introduction

Breast cancer is currently a major global health concern with high incidence and mortality rate in women, as there were 2.26 million cases reported in 2020, with the ASR (Age Standardized Incidence Rate per 100,000 people) as high as 47.8, according to the World Health Organization (WHO)^1^. Both chemotherapy and phototherapy have been regularly used for the treatment of breast cancer, especially in the breast-conserving therapy (BCT), and also in combination with surgery^2^. However, the effectiveness drops dramatically if the tumors are detected in the later stages. Moreover, these methods also suffer from a certain degree of limitations such as toxic side effects stemming from the limited selectivity of the currently available anti-cancer drugs^3,4^, as well as the lack of efficacy due to the development of resistance in some breast cancer strains^5^. Consequently, the development of novel anti-breast cancer agents with high efficacy, selectivity, and biocompatibility is highly in demand.

**Figure 1:**
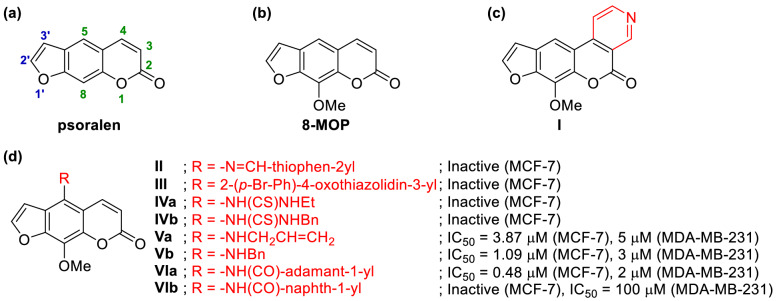
Example of psoralen derivatives and their anti-cancer activity.

For the past few decades, furocoumarin, a class of heterocyclic compound containing a furan fused with a coumarin, has proved to be a promising chemical scaffold for the development of novel drugs against breast cancer^6^. In early dates, natural-occurring psoralen (linear furocoumarin; Fig. 1a) derivatives such as 8-methoxypsoralen (8-MOP, Fig. 1b) were utilized clinically in a photochemotherapy^7^, due to their ability to intercalate DNA causing interstrand DNA crosslinks (ICL) upon photo-activated by UV irradiation, and hence inhibiting transcription and DNA replication^8^. This concept known as PUVA (psoralen and ultraviolet A) therapy is extensively used for the treatment of epidermal proliferative and inflammatory disorders^9^, and was shown to exert anti-proliferative effects in some breast cancer cell lines^10^. However, recent studies on psoralen derivative **I** incapable of inducing ICL formation (Fig. 1c) revealed the evidence that the anti-cancer effects of PUVA can also be mediated through a DNA-independent mechanism, involving the direct binding of psoralen with the catalytic autokinase domain of human epidermal growth factor receptor 2 (HER2; also known as erythroblastic oncogene B or ErbB2)^11^. This mechanism of action reflects in the high selectivity of PUVA towards HER2-overexpressing breast cancer cells such as SK-BR-3, which are known to be more aggressive and have a greater risk for disease progression^12^. However, the structure–activity relationship (SAR) study on the PUVA against breast carcinoma has never been reported.

Apart from photoactivation, psoralen derivatives have been demonstrated to possess moderate activity against breast cancer cell lines even in the absence of radiation^13^. Previous studies on the synthesis of novel psoralen derivatives focused on the structural modification at the C-5 position with substituents ranging from immine (**II**)^14^, thiazolidine (**III**)^14^, thiourea (**IV**)^14^, amine (**V**)^15^, and amide (**VI**)^15^, as shown in Fig. 1d. Among them, the amide derivative **VIa** bearing adamantoyl group exhibited promising activity towards both estrogenic (MCF-7) and non-estrogenic (MDA-MB-231) breast cancer cell lines, while little to no activity was found in the naphthoyl derivative **VIb**. The stark contrast in the activity, together with the lack of the scope of substituents encouraged us to further explore this amide series. Herein, we report the synthesis and anti-breast cancer activity of novel psoralen derivatives bearing different types of amide and other substituents against both hormone-dependent (T47-D) and hormone-independent (MDA-MB-231) breast cancer cells, compared with the normal cells. Moreover, since these derivatives have never been tested under UV irradiation, we also explored their phototoxicity against both HER2+ (SK-BR-3) and HER2− (MDA-MB-231) breast cancer cell lines. Since the actual targets are not unequivocally proven, we also carried out molecular docking with a potential protein target; HER2, in order to shed light on the potential binding interactions of these psoralen derivatives. Finally, the physicochemical properties, the essential parameters for discovering and developing new drugs, of these novel psoralen derivatives were also investigated.

## Results and discussion

### Synthesis of psoralen derivatives

**Figure 2:**
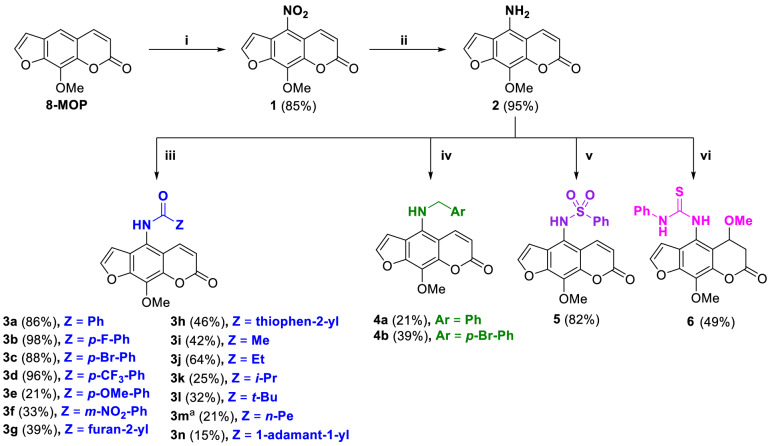
Synthesis of psoralen derivatives. (i) conc. HNO_3_, CH_3_COOH, rt; (ii) Sn (10.0 equiv.), 12 M HCl, EtOH, rt; (iii) acid chlorides (2.0–4.0 equiv.), K_2_CO_3_ (1.5 equiv.), DCM, rt or acetic anhydride (10.0 equiv.), K_2_CO_3_ (2.0 equiv.), pyridine (2.0 equiv.), cat. DMAP, DCM, rt; (iv) benzyl bromide (1.2–1.8 equiv.), K_2_CO_3_ (1.5 equiv.), acetone, 55 °C; (v) benzenesulfonyl chloride (1.1 equiv.), cat. DMAP, pyridine, 60 °C; (vi) phenylisothiocyanate (3.1 equiv.), MeOH, 65 °C; ^a^disubstituted product.

Four types of psoralen derivatives including amides (**3a**–**n**), amines (**4a**–**b**), sulfonamide (**5**), and thiourea (**6**) were synthesized following the synthetic route shown in Fig. 2. First, the commercially available 8-MOP was converted into the 5-nitro analog **1**, which was then reduced into the 5-amino analog **2** using tin powder as a reducing agent^16,17^. Next, **2** was treated with different acid chlorides or acid anhydrides to afford the corresponding amide derivatives **3a**–**n** with the yields ranging from 15 to 98%. Since the previous work reported that the amide derivatives showed promising results against breast cancer cell lines^15^, in this work we then emphasized on the structure variation of the amide derivatives, with the substitution patterns ranging from aromatic amides bearing substituents with various electronic and steric effects (**3a**–**f**), heteroaromatic amides (**3g**–**h**), and aliphatic amides with different carbon chain lengths (**3i**–**n**). The adamantoyl derivative **3n** (**VIa**), the best compound reported in the literature^15^, was also included as a control. Notably, the acylation using hexanoyl chloride resulted in the unusual disubstituted analog **3m** in 21% yield as confirmed by spectroscopic analysis. Apart from the amide derivatives, the amine analogs **4a** and **4b** bearing benzyl and 4-bromobenzyl substituents, respectively, were synthesized by the alkylation of **2** in moderate yields^15^. The sulfonation of **2** with benzenesulfonyl chloride afforded sulfonamide derivative **5** in 82% yield^18^. Lastly, the phenylthiourea analog **6** was synthesized by treating **2** with phenylisothiocyanate in methanol in 49% yield^19^. However, a thorough characterization with ^1^H, ^13^C, and HRMS revealed the lack of double bond on the coumarin part and the presence of a methoxy substituent at the C-6 position, which could be resulted from the nucleophilic Michael addition of MeOH used as the solvent.

### Anti-breast cancer activity and cancer chemoprevention

Twenty of the synthesized psoralen derivatives were subjected to the evaluation of their cytotoxic effect against two breast cancer cell lines: MDA-MB-231 (hormone-independent) and T47-D (hormone-dependent), compared with three positive controls: doxorubicin, tamoxifen citrate, and lapatinib, via an MTT assay. Additionally, the cytotoxicity towards normal cells, human embryonal lung fibroblast cell MRC-5 was also assessed. Table 1 shows the %inhibition at the concentration of 50 μg mL^−1^ and the IC_50_ values after 48 h treatment of each compound without UVA irradiation. Almost all psoralen derivatives showed no cytotoxicity towards MRC-5, except only for **3f** with an IC_50_ value of 77.33 µM, which was still less toxic than those of the three positive controls, implying their high safety profile. For the anti-cancer activity, most of the compounds exhibited modest cytotoxicity towards both cell lines, with the IC_50_ of 10.14 µM for the most active compound against T47-D. We surmised that the cell lines used in our work were relatively less sensitive, as can be clearly observed for the adamantoyl derivative **3n** (**VIa**) with the IC_50_ greater than 100 µM against MDA-MB-231, compared with the previously reported value (IC_50_ = 2 µM)^15^. This lack of sensitivity of MDA-MB-231 was also observed for the positive control tamoxifen citrate whereby in our experiment exhibited much higher IC_50_ value (IC_50_ = 66.00 µM vs 1.6 µM)^15^. In fact, breast cancer cell lines could exhibit DNA alterations from the serial cultivation and cause phenotypical changes to cells resulting in variable sensitivities to drugs^20^. Thus, in our case, the cancer cell lines used in this work could be more resistant than the previous report^15^.

**Table 1:** Cytotoxic and aromatase inhibitory activities (IC_50_ [µM]) of psoralen derivatives.

Comp.			Cytotoxic activity			Selectivity Index^d^ (SI)	Aromatase inhibitory activity
	R^1^	R^2^	MDA-MB-231^a^	T47-D^b^	MRC-5^c^		
**8-MOP**	–H		I (6%)^e^	I (16%)^e^	I (13%)^e^	–	I (21%)^f^
**1**	–NO_2_		I (39%)^e^	> 100	I (49%)^e^	> 1.2	I (34%)f
**2**	–NH_2_		I (3%)^e^	I (28%)^e^	I (5%)^e^	–	I (9%)^f^
**3a**	H		I (25%)^e^	I (45%)^e^	I (16%)e	–	I (3%)^f^
**3b**	H		I (34%)^e^	I (49%)^e^	I (11%)^e^	–	I (6%)^f^
**3c**	H		91.33 ± 7.84	10.14 ± 0.53	I (45%)^e^	> 11.9	I (13%)f
**3d**	H		I (35%)^e^	13.64 ± 0.26	I (41%)^e^	> 9.1	I (10%)f
**3e**	H		I (19%)^e^	I (38%)^e^	I (6%)^e^	–	I (15%)^f^
**3f**	H		71.01 ± 2.34	49.77 ± 0.50	77.33 ± 3.52	1.6	I (4%)^f^
**3g**	H		I (0.5%)^e^	I (24%)^e^	I (4%)^e^	–	I (16%)f
**3h**	H		I (8%)^e^	I (25%)^e^	I (3%)^e^	–	I (8%)^f^
**3i**	H		I (6%)^e^	I (20%)^e^	I (12%)^e^	–	I (7%)^f^
**3j**	H		I (3%)^e^	I (22%)^e^	I (4%)^e^	–	I (8%)^f^
**3k**	H		I (4%)^e^	I (36%)^e^	I (18%)^e^	–	I (8%)^f^
**3l**	H		I (1%)^e^	I (21%)^e^	I (6%)^e^	–	I (14%)^f^
**3m**			I (5%)^e^	I (15%)^e^	I (10%)e	–	9.4 ± 0.4
**3n**	H		> 100	43.06 ± 0.09	I (0%)^e^	> 3.0	I (5%)^f^
**4a**	H		> 100	31.12 ± 1.54	I (0%)^e^	> 5.0	I (46%)^f^
**4b**	H		94.35 ± 4.77	10.39 ± 0.79	I (0%)^e^	> 12.0	0.9 ± 0.2
**5**	H		I (36%)^e^	87.87 ± 6.34	I (9%)^e^	> 1.5	I (33%)^f^
**6**			91.86 ± 4.49	> 100	I (3%)^e^	> 1.2	I (18%)^f^
Doxorubicin			3.16 ± 0.26	1.46 ± 2.79	3.07 ± 0.22	2.1	–
Tamoxifen citrate			66.00 ± 2.48	20.86 ± 1.31	67.56 ± 10.50	3.2	–
Lapatinib			53.37 ± 0.31	9.78 ± 0.46	48.75 ± 9.45	5.0	–
Letrozole			–	–	–		2.6 ± 0.1 nM

Results are obtained from three independent biological repeats; ^a^Hormone-independent breast cancer; ^b^Hormone-dependent breast cancer; ^c^Human embryonal lung fibroblast cell; ^d^Selectivity Index (SI) = IC_50_ for MRC-5/IC_50_ for T47-D; ^e^%Inhibition at the concentration of 50 mg mL^−1^; ^f^%Inhibition at the concentration of 12.5 µM; I = %inhibition lower than 50% at a specified concentration; – not determined.

Nevertheless, the cytotoxic results emphasize the importance of C-5 substituents in the anti-breast cancer activity of the synthesized psoralen derivatives. In general, the amide derivatives **3a**–**n** were relatively less active than the other series, with the benzamide derivatives (**3a**–**f**) exhibiting much greater cytotoxicity than the heteroaromatic (**3g**–**h**) and the aliphatic (**3i**–**n**) derivatives. Among the benzamide series, electron-withdrawing groups such as *p*-Br (**3c**), *p*-CF_3_ (**3d**), and *m*-NO_2_ (**3f**) showed better activity compared to other analogs. Likewise, the introduction of the *p*-Br in the benzyl amine analog **4b** also resulted in the increase in activity compared with the non-substituted analog **4a**. The sulfonamide (**5**) and thiourea (**6**) analogs were shown to inhibit breast cancer to some extent; however, the IC_50_ were relatively high for both cell lines.

Comparing between the two breast cancer cell lines, the psoralen derivatives were less sensitive toward MDA-MB-231 cells with the best IC_50_ values only at 71.01 µM from compounds **3f**. However, they appeared to exhibit good activity with T47-D and some of which exhibited the more potent IC_50_ values than the positive drug tamoxifen citrate. This included compounds **3c**, **3d**, and **4b** with the IC_50_ values of 10.14, 13.64, and 10.39 µM, respectively. Gratifyingly, these three derivatives also presented high selectivity. The derivatives **3c**, **3d**, and **4b** had selectivity index (SI) with T47-D more than 11.9, 9.08, and 12.0, which were at least ca. 6.7, 2.8 and 3.7-fold superior to that of tamoxifen citrate (SI = 3.23), the current approved therapeutic drug for estrogen positive breast cancer.

Estrogens are the critical risk factor for breast cancer initiation and progression and the aromatase enzyme involves in the final step conversion of androgens to estrogens^21,22^. As more than 70% of all breast cancers are classified as ER+ (estrogen receptor positive) and estrogen dependent, identification of compounds that can interfere this molecular mechanism could be clinically useful for management of hormone dependent breast cancer. In this study, the synthesized psoralen derivatives were also tested for aromatase inhibitory activity (AIA) which is considered to be one of chemopreventive mechanisms for hormone dependent breast cancer. According to Table 1, although only compounds **3m** and **4b** exhibited anti-aromatase activity, both of them possessed very high efficacy with the IC_50_ values of 9.4 and 0.9 µM, respectively. Further analysis to find any correlation between the cytotoxicity of the hormone dependent breast cancer T47-D and anti-aromatase activity revealed that, even though the tertiary amide **3m** has good chemopreventive activity in AIA with the IC_50_ = 9.4 µM, it has no anti-cancer activity. Moreover, the compounds with good anti-breast cancer activity such as **3c** and **3d**, even so, showed no chemopreventive activity in AIA. Hence, it might be said that the inhibition of aromatase enzyme and anti-breast cancer activity in T47-D cells were not necessarily correlated. Nevertheless, secondary amine **4b** was highly potent in both anti-breast cancer activities (cytotoxic activity in T47-D cells, IC_50_ = 10.39 µM and chemo-preventive activity in AIA, IC_50_ = 0.9 µM). As the preferable concurrent cytotoxic and aromatase inhibitory activities could provide synergistic potential for management of estrogen-dependent breast cancer, the structure of bromobenzyl amine **4b** is particularly promising for further study to improve anti-cancer activity and further elucidate the mechanism.

Psoralen derivatives have been reported to be associated with various aromatase and estrogen related events. For example, administer of 5-MOP (bergapten) and 8-MOP to female rats significantly reduced circulating estrogen levels in a dose-dependent manner^23^. 8-MOP decreased both the amount of aromatase protein in the ovary and the level of circulating estradiol in female Wistar rats^24^. Bergapten reduced the levels of estrogen receptor in MCF-7 breast cancer cells as well as in the tamoxifen-resistant clone^25^. In the in-vitro ERα antagonist potential, xanthotoxol, bergapten, angelicin, psoralen and isoimperatorin antagonized ETRα activity in MCF-7 cells with IC_50_ values of 0.7, 1.2, 11.0, 24.1 and 54.3 μM, respectively^26^. On estrogens and growth factor signaling in breast cancer, psoralens may act by inhibiting aromatase enzyme, metalloproteinases (MMPs) and CYP enzymes^6^. However, to date, the supporting evidence on a role of psorlarens as an aromatase inhibitor is quite elusive. The results from this work revealed some psorlaren derivatives that could exert aromatase inhibitory activity, thus this finding could be initial point for futher investigatation to the more potent aromatase inhibitor. Additionally, as psoralens have been reported to be related to several anti-breast cancer mechanistic pathways^6^, targeting multiple pathways by psoralens which could promote synergistic effects for cancer treatment is worth to be explored.

Apart from anti-aromatase activity, all psoralen derivatives were also evaluated in several chemopreventive assays that represented different defense mechanisms against cancer, including radical scavenging of 1,1-diphenyl-2-picrylhydrazyl (DPPH) assay, inhibition of superoxide formation by inhibiting xanthine oxidase (IXO), antioxidant properties by scavenging of superoxide anion radical generation induced by 12-*O*-tetradecanoyl-phorbol-13-acetate (TPA) in differentiated HL-60 human promyelocytic leukemia cells (HL-60 antiox.) and by reaction of xanthine/xanthine oxidase (XXO), anti-inflammatory by inhibition of lipoxygenase (LOX). The results showed that the majority of compounds had low to moderate chemopreventive activities (see Supplementary Table S1).

### Phototoxicity

**Figure 3:**
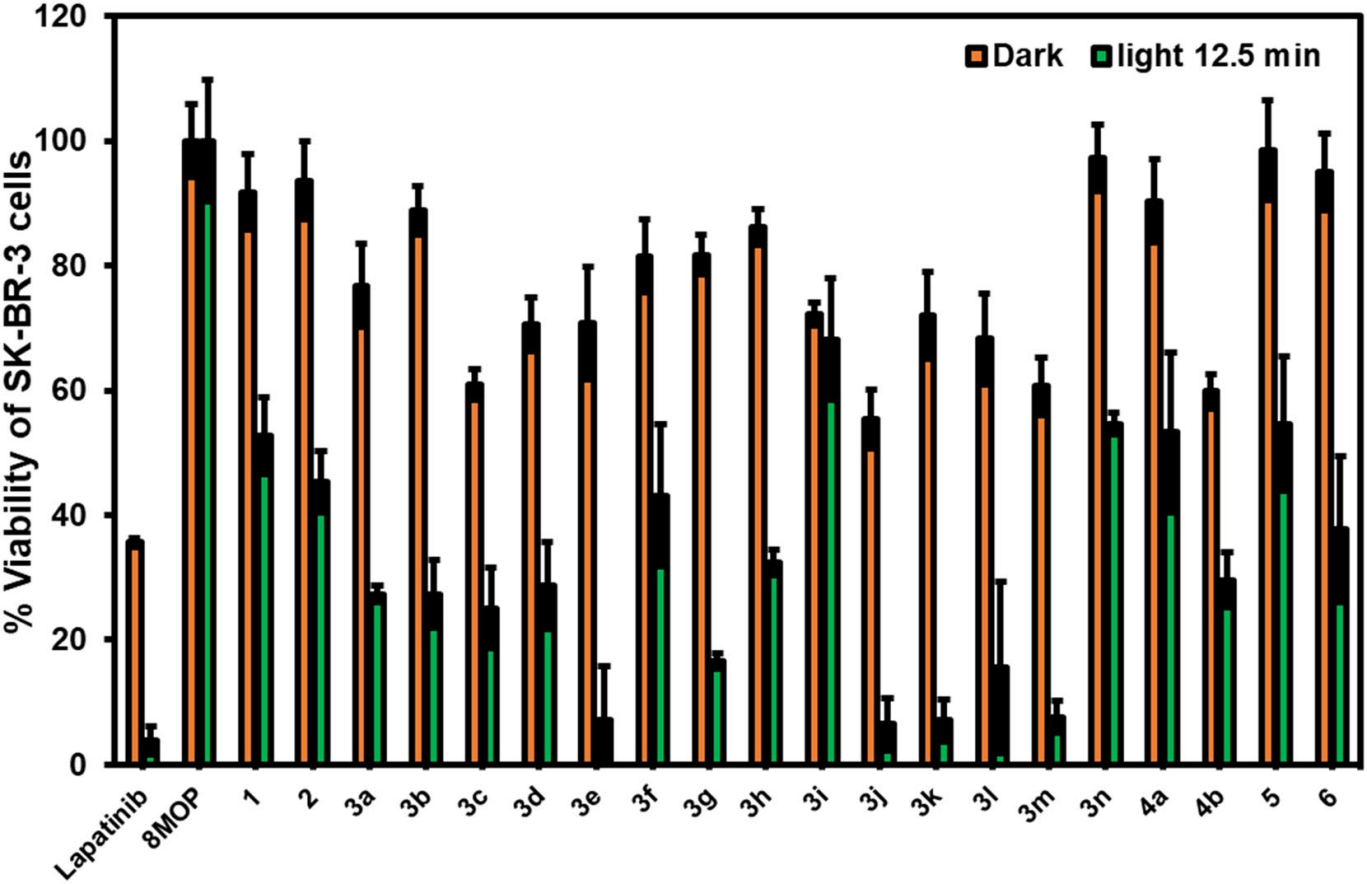
Dark and light-activated cytotoxicity of psoralen derivatives against SK-BR-3 at the concentration of 50 µM; orange bar = %viability in the dark, green bar = %viability under irradiation with UVA (2.0 J cm^−2^).

All the synthesized compounds, and a positive control lapatinib were first preliminarily tested for their cytotoxicity towards an HER2+ breast cancer cells, SK-BR-3, both in the absence and the presence of UVA irradiation (2.0 J cm^−2^) as shown in Fig. 3.

Strikingly, the cytotoxicity towards SK-BR-3 for most of the psoralen derivatives were immensely enhanced by the UVA irradiation, except for the parent 8-MOP, which showed little to no activity neither in the absence nor the presence of UV light. In contrast with the results of MDA-MB-231 and T47-D (Table 1), the amide derivatives **3a**–**n** exhibited slightly greater dark cytotoxicity against the SK-BR-3, compared with the amine, sulfonamide, and thiourea derivatives. Notably, the UVA irradiation enhanced the cytotoxicity of the furanyl (**3g**) and aliphatic amides (**3j**–**m**) to a much greater extent than those with aromatic or thiophene rings. Furthermore, the size of the aliphatic substituent contributed greatly to the phototoxicity: the compound bearing a small acetyl (**3i**) or very bulky adamantoyl (**3n**) groups were found to produce relatively higher %viability of the cancer cells. In addition, the benzamide derivative bearing an electron-donating *p*-OMe (**3e**) also showed very promising anti-cancer activity. In this regard, we selected the compounds that gave the %viability under UVA irradiation lower than 20% for further investigation. The selected compounds were screened for their IC_50_ values against both HER2+ breast cancer (SK-BR-3), and HER2− breast cancer (MDA-MB-231) in both dark and light-activated conditions (Table 2). Additionally, a negative control study was also performed with SK-BR-3 and MDA-MB-231 cells in the absence of tested compound under UVA irradiation (See Fig S7., Supporting Information). The % cell viability of > 85% of both SK-BR-3 and MDA-MB-231 cells after UVA irradiation suggested that the UVA radiation exhibited marginal significant effect toward both cell lines.

**Table 2:** Dark and light-activated cytotoxicity of psoralen derivatives against breast cancer cells.

Comp	IC_50_ (µM) dark		IC_50_ (µM) with UVA^a^	
	SK-BR-3^b^	MDA-MB-231^c^	SK-BR-3^b^	MDA-MB-231^c^
**8-MOP**	> 100	> 100	24.66 ± 3.02	> 100
**3e**	> 100	> 100	66.72 ± 2.75	> 100
**3g**	> 100	> 100	2.71 ± 0.84	> 100
**3j**	> 100	> 100	3.05 ± 1.02	> 100
**3k**	> 100	> 100	66.09 ± 5.21	> 100
**3l**	> 100	> 100	48.14 ± 3.07	> 100
**3m**	> 100	> 100	27.09 ± 1.20	> 100
Lapatinib	25.27 ± 0.99	73.00 ± 2.53	0.16 ± 0.03	8.60 ± 0.82

Results are obtained from three independent biological repeats; ^a^Irradiation with UVA (2.0 J cm^−2^); ^b^HER2+ breast cancer; ^c^HER2− breast cancer.

In the experiment with the tested compounds, all the compounds were found to be inactive against both cancer cells in the absence of UV irradiation. However, a stark contrast in the phototoxicity between the two cell lines was observed. For SK-BR-3, the selected psoralen derivatives exhibited moderate to exceptional phototoxicity with the IC_50_ ranging from 2.71 to 66.72 µM. In contrast, no phototoxicity was observed for all derivatives against MDA-MB-231, which is in consistent with the previously reported finding by Xia and co-workers, suggesting that these psoralen derivatives may exert phototoxicity via the mechanism involving the interaction with HER2^11^. To our surprise, the most active compound was found to be the amide analog **3g** bearing a furanyl group, giving the IC_50_ of 2.71 µM which is almost tenfold increase from the parent 8-MOP.

It is worth mentioning that in contrast to the psoralen derivatives, the strong phototoxicity of a positive control lapatinib could be clearly observed in both SK-BR-3 and MDA-MB-231. Since the selective phototoxicity activity of 8-MOP towards HER2+ breast cancer has been proposed to associate with the catalytic autokinase domain of the HER2^11^, while it is well-known that lapatinib binds to the ATP-binding sites of both HER1 and HER2^27^. Therefore, it is conceivable that the strong phototoxicity of lapatinib might involve other mechanisms of action as the activity could be found in both HER2+ and HER2− breast cancer cell lines. It should also be noted that lapatinib had an anti-cancer activity even without UVA irradiation (Table 2) and had significant toxicity towards normal cells (Table 1), hence resulting in a limited selectivity. Consequently, the PUVA phototherapy with these psoralen derivatives could provide a unique selectivity not only towards the breast cancer cells, but also in the area of the biological action, which could be valuable for potential targeted therapy. Nevertheless, it should also take caution that both lapanib and the parent 8-MOP are known to have phototoxic potential as determined by 3T3 NRU assay^28,29^; therefore, the cutaneous adverse effects associated with these compounds is neccessay to be identified. Additionally, mitigation of the adverse effects of UVA by using therapeutic approach such as X-PACT (X-ray Psoralen Activated Cancer Therapy)^30^, should be worthwhile considered in the development of psoralen derivatives as breast cancer targeted therapy.

### Molecular docking studies

**Figure 4:**
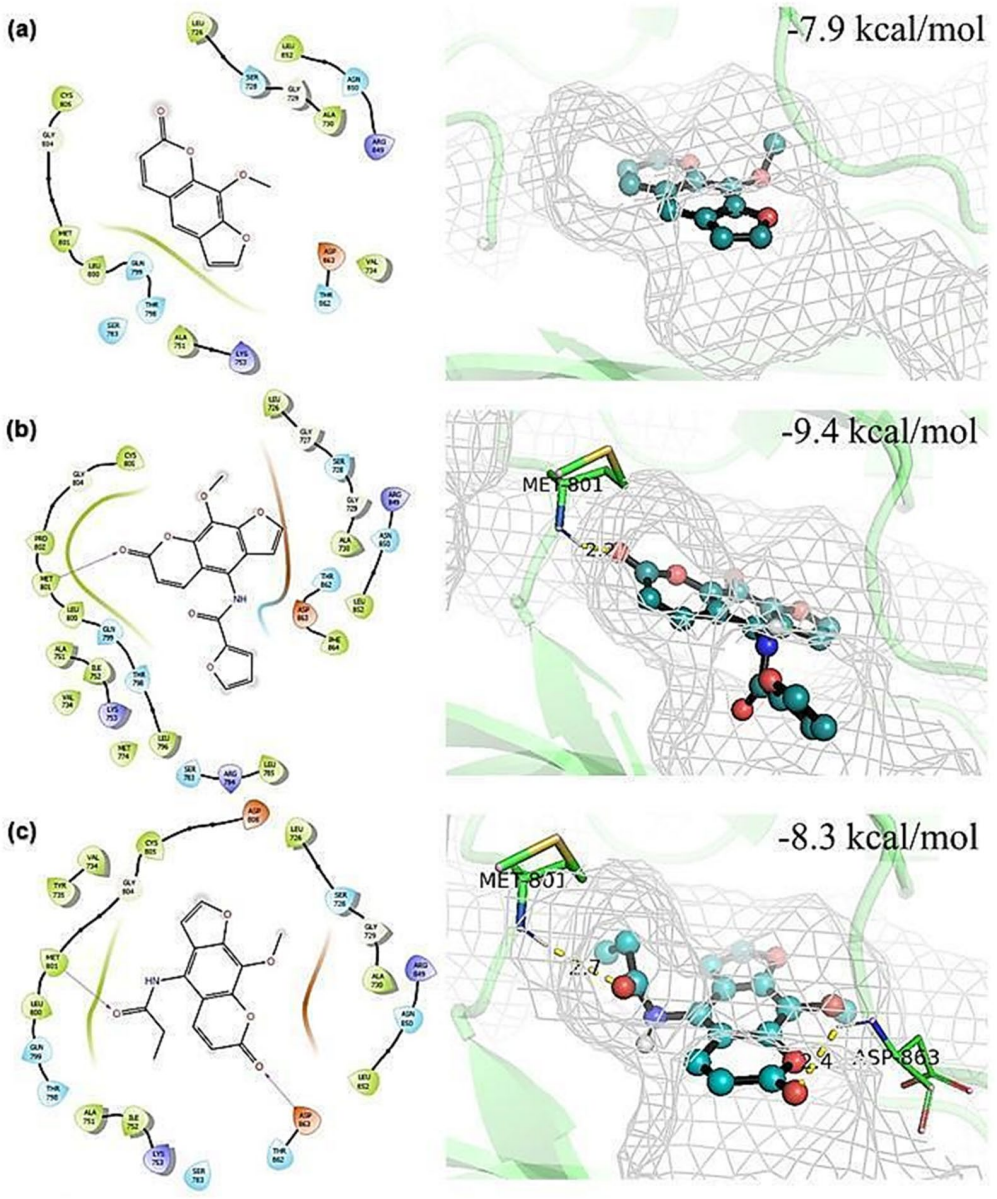
2D and 3D representations of top-scoring poses obtained from molecular docking models between psoralen derivatives: 8-MOP (**a**), **3g** (**b**), or **3j** (**c**) and HER2 (PDB code: 3PP0)^44,45^. The purple arrows represent the hydrogen bonding. Ligand molecules shown as ball and stick models are colored cyan (carbon), red (oxygen), blue (nitrogen), and white (polar hydrogen). Hydrogen bonding is shown in yellow dashed lines to amino acid residues, and the distance (Å) between atoms of amino acids and ligand is displayed.

The mechanism in which psoralens can inhibit breast cancer cells with UVA irradiation have been reported via multiple pathways, such as through DNA intercalation and inhibition of HER2^11^. From our study, the difference in the phototoxicity between MDA-MB-231 and SK-BR-3 demonstrated by our newly synthesized psoralen derivatives strongly suggests HER2 as a plausible target. In order to analyze the molecular binding between the psoralen derivatives and HER2, we performed molecular docking study between all seven ligands in Table 2 against HER2 using Autodock Vina (Supplementary Table S2). The docking results of three representative ligands 8-MOP (parent ligand, IC_50_ = 24.66 µM), **3g** (the most active, IC_50_ = 2.71 µM) and **3j** (the second most active, IC_50_ = 3.05 µM) docked poses and docking scores of the corresponding ligands are shown in Fig. 4.

As shown in Fig. 4, HER2 can suitably accommodate all the psoralen ligands in the same binding pocket. This result is coherent with the work by Xia et al., reporting that psoralen derivative was bound to the HER2 at the aa 861–868 and aa 869–883 regions, albeit upon UVA irradiation^11^. The docking scores of ligands, 8-MOP, **3g**, and **3j** are − 7.9, − 9.4 and − 8.3 kcal mol^−1^, respectively. A less negative molecular docking score of 8-MOP suggests less favorable interactions between the predicted pose of 8-MOP with the HER2 binding site, compared to **3g**, and **3j**. Both **3g** and **3j** docked poses revealed hydrogen-bonds with Met801 residue (2.2 and 2.7 Å) and with ASP863 (2.4 Å). Notably, the hydrogen bonds with Met801 residue are also seen in the co-crystallized inhibitor as reported by Aertgeerts et al.^31^. Therefore, these favorable interactions of **3g** and **3j** at the active site of HER2 could be a reason that contributes towards the exceptional phototoxicity and selectivity of these derivatives against the HER2-overexpressing breast cancer cells.

### Cell morphology

**Figure 5:**
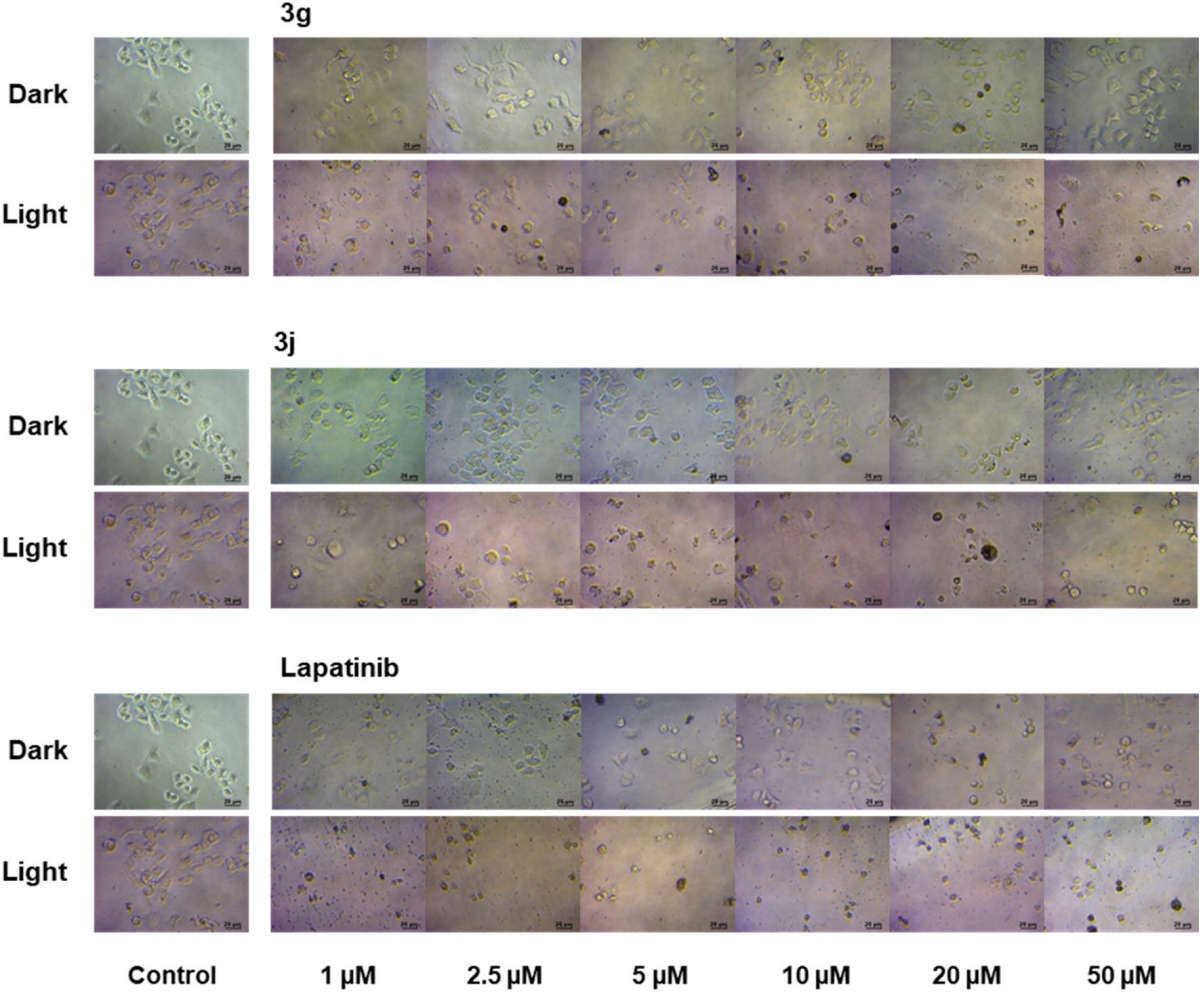
The morphology of SK-BR-3 cells treated with different concentrations of compounds **3g**, **3j**, and lapatinib at 48 h under dark and light activation (irradiation with UVA 2.0 J/cm^2^). Cells treated with 1% DMSO served as controls.

To clarify their anti-proliferative effects, the morphology of SK-BR-3 cells treated with different concentrations of compounds **3g**, **3j**, and lapatinib under dark and light activation was observed using a light microscope. Notably, the difference of cell morphology between dark and light activation of the **3g** and **3j**-treated SK-BR-3 cells could be clearly presented (Fig. 5). The cells under dark condition maintained morphology of intact cells after treatment at 48 h, while under light activation using UVA 2.0 J cm^−2^, the morphology of treated SK-BR-3 cells was significantly changed. These results were in agreement with the cytotoxicity results whereby the cytotoxic activity of the **3g** and **3j**-treated SKBR-3 cells could be observed only under UVA irradiation (Table 2). Furthermore, the increase in the apoptotic index, which is characterized by the features such as cell shrinkage, plasma membrane blebbing, and cell disintegration into apoptotic bodies, was presented in a dose dependent manner in both the **3g-** and **3j**-treated SK-BR-3 cells. At higher concentrations (10, 20 or 50 µM), the cells were almost completely broken down, while at lower concentrations (1, 2.5, or 5 µM), varying degrees of cell shrinkage and membrane blebbing were still depicted. For lapatinib, in contrast to **3g** and **3j**, the morphological changes indicating apoptosis of the treated cells were depicted under both dark and light activation in a dose dependent manner. Nevertheless, the higher degree of apoptotic index was detected under light activation.

Based on these finding, the significant inhibition of SK-BR-3 cells under UVA irradiation of **3g** and **3j** appeared to be related to induction of cell apoptosis, which is corresponding to the previous report regarding the ability PUVA to block HER2 signaling and induce tumor cell apoptosis^11^. However, the apoptosis induced by lapatinib may involve in different mechanism as dissimilar results could be observed.

### The predicted physicochemical properties

In order to evaluate the druggability of the compounds, the physicochemical properties and analysis by Lipinski's rule of five were conducted using SwissADME website service. All the calculated parameters are presented in Table 3. Although the introduction of the C-5 substituents generally leads to an increase in the TPSA, the values remain less than 140 Å^2^. Combining with the number of rotatable bonds of less than 10, this suggests that the compounds might exhibit good oral bioavailability^32^. Moreover, the increase in the lipophilicity for these derivatives also benefits cell permeability^33^. However, these derivatives suffer from a slight decrease in the aqueous solubility; nevertheless, based on Log S, these derivatives are categorized within the “soluble” or “moderately soluble” classes, which are sufficient to be used as a drug^34^. For example, the aqueous solubility of the most active compound, **3g**, was predicted to be 182 µM, which is well above its working concentration (IC_50_ with UVA = 2.71 µM against SK-BR-3). Finally, no violation of Lipinski’s rule of five was observed for all the compounds as the molecular weight, the number of hydrogen bond acceptors and donors, and the lipophilicity are all within the acceptable limits^35^.

**Table 3:** Predicted physicochemical properties of psoralen derivatives^46,47^.

Comp.	MW	Fraction Csp3^a^	Rotatable bonds	HBA^b^	HBD^c^	TPSA^d^	MLOGP^e^	ESOL Log S^f^	Lipinski #violations^g^
**8-MOP**	216.19	0.08	1	4	0	52.58	1.18	− 2.98	0
**1**	261.19	0.08	2	6	0	98.4	0.21	− 2.99	0
**2**	231.21	0.08	1	4	1	78.6	0.61	− 2.6	0
**3a**	335.31	0.05	4	5	1	81.68	1.94	− 4.18	0
**3b**	353.31	0.05	4	6	1	81.68	2.32	− 4.34	0
**3c**	414.21	0.05	4	5	1	81.68	2.55	− 5.09	0
**3d**	403.31	0.1	5	8	1	81.68	2.5	− 5.02	0
**3e**	365.34	0.1	5	6	1	90.91	1.63	− 4.24	0
**3f**	380.31	0.05	5	7	1	127.5	1.06	− 4.23	0
**3g**	325.27	0.06	4	6	1	94.82	0.7	− 3.74	0
**3h**	341.34	0.06	4	5	1	109.92	1.5	− 4.22	0
**3i**	273.24	0.14	3	5	1	81.68	0.67	− 2.56	0
**3j**	287.27	0.2	4	5	1	81.68	0.92	− 3.03	0
**3k**	301.29	0.25	4	5	1	81.68	1.17	− 3.46	0
**3l**	315.32	0.29	4	5	1	81.68	1.42	− 3.75	0
**3m**	427.49	0.46	12	6	0	89.96	2.51	− 5.32	0
**3n**	393.43	0.48	4	5	1	81.68	2.77	− 4.89	0
**4a**	321.33	0.11	4	4	1	64.61	2.15	− 4.52	0
**4b**	400.22	0.11	4	4	1	64.61	2.76	− 5.42	0
**5**	371.36	0.06	4	6	1	107.13	1.24	− 4.19	0
**6**	398.43	0.2	6	5	2	114.05	1.55	− 3.95	0

^a^The ratio of *sp*^3^ hybridized carbons over the total carbon count of the molecule; ^b^hydrogen bond acceptor; ^c^hydrogen bond donor; ^d^topological polar surface area [Å^2^]; ^e^lipophilicity: Moriguchi octanol–water partition coefficient (MLOGP) is based on quantitative structure-logP relationships, by using topological indexes; ^f^log of the aqueous solubility measured in mol L^−1^, according to the Estimating Aqueous Solubility (ESOL); ^g^Lipinski’s rule of five (MW ≤ 500, MLogP ≤ 4.15, HBA ≤ 10, and HBD ≤ 5).

## Materials and methods

### Synthesis and identification of psoralen derivatives

All reagents and solvents were obtained from Sigma-Aldrich (St. Louis, MO, USA), TCI Chemicals (Tokyo, Japan), Fluorochem (Hadfield, Derbyshire, UK) and Merck (Darmstadt, Germany). All solvents for column chromatography from RCI Labscan (Samutsakorn, Thailand) were distilled before use. ^1^H, ^13^C and ^19^F NMR spectra were recorded on Bruker Avance (400, 101 and 376 MHz) and Jeol Avance (500 and 126 MHz). Chemical shifts are reported as δ values in parts per million (ppm) relative to tetramethylsilane (δ 0.00) or the solvent residue in DMSO-*d*_*6*_ (δ 2.50) or CDCl_3_ (δ 7.26). Data are reported as follows; chemical shift (multiplicity, coupling constants in Hz, integrate intensity, assignment). Abbreviations of multiplicity were as follows; s: singlet, d; doublet, t: triplet, q: quartet, m: multiplet, br: broad. Chemical shifts for ^13^C NMR are reported as δ values in parts per million (ppm) relative to DMSO-*d*_*6*_ (δ 39.52) and CDCl_3_ (δ 77.16) as internal standard. High-resolution mass spectra (HRMS) data were obtained with micro-TOF mass spectrometer. IR spectra were recorded using the Thermo Scientific Nicolet iS50 FTIR spectrometer with ATR module and are reported in wave number (cm^−1^). Reactions were monitored by thin-layer chromatography (TLC) using aluminium Merck TLC plates coated with silica gel 60 F254. The reactions were monitored by TLC (ethyl acetate:hexanes, 1:1, 1:2, 1:3, 2:3, 1:4, 1:9, 100%DCM, and DCM:MeOH, 95:5 v/v). Normal phase column chromatography was performed using silica gel 60 (0.063–0.200 mm, 70–230 mesh ASTM, Merck, Darmstadt, Germany). Melting points were measured using a melting point apparatus (Griffin) and are uncorrected.

#### 9-Methoxy-4-nitro-7*H*-furo[3,2-*g*]chromen-7-one (1)

The title compound was synthesized using a slightly modified literature procedure using 8-MOP (2.20 g, 10.00 mmol) in glacial acetic acid (21.0 mL), and conc. HNO_3_ (17.1 mL)^16^. The precipitate was collected and washed with DI water to give the title compound. Yellow solid; 85% yield; **Mp**: 237–238 °C; **HPLC**: 99.11% purity; **TLC** (100%DCM): R_f_ = 0.45; ^**1**^**H NMR** (400 MHz, CDCl_3_): δ 8.78 (d, *J* = 10.2 Hz, 1H), 7.86 (d, *J* = 2.3 Hz, 1H), 7.42 (d, *J* = 2.3 Hz, 1H), 6.63 (d, *J* = 10.2 Hz, 1H), 4.49 (s, 3H); ^**13**^**C NMR** (101 MHz, CDCl_3_): δ 165.1, 158.5, 157.7, 149.6, 145.3, 145.1, 139.4, 124.7, 118.8, 112.4, 107.6, 61.7; **IR** (neat): 3156 (C–H), 1736 (C=O), 1571 (C=C), 1497 (N=O), 1314 (N=O), 1282 (C–O), 1265 (C–O) cm^−1^; Data are consistent with literature values^36^.

#### 4-Amino-9-methoxy-7***H***-furo[3,2-***g***]chromen-7-one (2)

The title compound was synthesized using a slightly modified literature procedure using **1** (2.09 g, 8.00 mmol) in methanol (15.0 mL), tin powder (3.80 g, 40.00 mmol), and conc. hydrochloric acid (40.0 mL)^17^. The precipitate was collected and washed with hexanes to give the title compound. Green solid; 95% yield; **Mp**: 238–239 °C; **HPLC**: 98.12% purity; **TLC** (DCM:MeOH, 95:5 v/v): R_f_ = 0.60; ^**1**^**H NMR** (500 MHz, DMSO-*d*_*6*_): δ 8.32 (d, *J* = 9.8 Hz, 1H), 7.85 (d, *J* = 2.2 Hz, 1H), 7.20 (d, *J* = 2.2 Hz, 1H), 6.49 (br s, 2H), 6.12 (d, *J* = 9.8 Hz, 1H), 3.90 (s, 3H); ^**13**^**C NMR** (126 MHz, DMSO-*d*_*6*_): δ 160.8, 150.5, 145.0, 144.7, 141.5, 136.6, 123.4, 111.7, 108.6, 106.2, 100.5, 61.7; **IR** (neat): 3478 (N–H), 3383 (N–H), 3137 (C–H), 3107 (C=C), 2957 (C–H), 1701 (C=O), 1647 (N–H), 1587 (C=C), 1480 (C=C), 1446 (C=C), 1050 (C–O) cm^−1^; **HRMS** (ESI^+^): m/z calcd for C_12_H_9_NO_4_Na^+^ [M+Na]^+^ 254.0429, found 254.0428. Data are consistent with literature values^36^.

#### General procedure for the synthesis of 3a–n^15^

To a solution of **2** in DCM was added the corresponding acid chloride (4.0 equiv.) and K_2_CO_3_ (1.5 equiv.) at room temperature. The reaction mixture was refluxed overnight. Upon completion, the reaction mixture was quenched with DI water. The resulting mixture was neutralized by sat. NaHCO_3_ and extracted with EtOAc (3 times). The combined organic layers were washed with brine, dried, filtered, then concentrated in vacuo to give the amide derivatives.

#### ***N***-(9-Methoxy-7-oxo-7***H***-furo[3,2-***g***]chromen-4-yl)benzamide (3a)

The crude product was purified by silica gel column chromatography (eluent: EtOAc:hexanes = 1:9–1:1) to provide the title compound. Off-white solid; 86% yield; **Mp**: 220–222 °C; **HPLC**: 98.67% purity; **TLC** (EtOAc:hexanes, 1:1): R_f_ = 0.67; ^**1**^**H NMR** (400 MHz, DMSO-*d*_*6*_): δ 10.60 (br s, 1H), 8.09–7.99 (m, 4H), 7.61 (d, *J* = 7.5 Hz, 1H), 7.54 (t, *J* = 7.5 Hz, 2H), 6.92 (d, *J* = 2.3 Hz, 1H), 6.41 (d, *J* = 9.8 Hz, 1H), 4.16 (s, 3H); ^**13**^**C NMR** (101 MHz, DMSO-*d*_*6*_): δ 166.4, 159.6, 147.4, 147.2, 142.9, 141.7, 133.9, 132.2, 130.8, 128.7, 128.2, 123.8, 121.8, 113.9, 112.9, 106.5, 6.4; **IR** (neat): 3235 (N–H), 3060 (C–H), 2944 (C–H), 1726 (C=O), 1647 (C=O), 1593 (C=C), 1145 (C–O) cm^−1^; **HRMS** (ESI^+^): m/z calcd for C_19_H_13_NO_5_Na^+^ [M+Na]^+^ 358.0686, found 358.0681.

#### 4-Fluoro-***N***-(9-methoxy-7-oxo-7***H***-furo[3,2-***g***]chromen-4-yl)benzamide (3b)

The crude product was washed with hexanes to give the title compound. Gray solid; 98% yield; **Mp**: 276–277 °C; **HPLC**: 98.91% purity; **TLC** (EtOAc:hexanes, 1:1): R_f_ = 0.60; ^**1**^**H NMR** (400 MHz, DMSO-*d*_*6*_): δ 10.60 (br s, 1H), 8.11 (dd, *J* = 8.5, 5.4 Hz, 2H), 8.05 (d, *J* = 2.3 Hz, 1H), 8.02 (d, *J* = 9.8 Hz, 1H), 7.36 (t, *J* = 8.6 Hz, 2H), 6.90 (d, *J* = 2.3 Hz, 1H), 6.39 (d, *J* = 9.7 Hz, 1H), 4.14 (s, 3H); ^**13**^**C NMR** (101 MHz, DMSO-*d*_*6*_): δ 166.0, 165.6, 159.9, 147.7, 147.4, 143.1, 141.9, 131.2 (d, *J* = 9.3 Hz), 130.6 (d, *J* = 2.9 Hz), 124.1, 121.8, 115.9, 115.7, 114.2, 113.2, 106.7, 61.6; ^**19**^**F NMR** (376 MHz, DMSO-*d*_*6*_): δ –108.20 (tt, *J* = 8.6, 3.2 Hz); **IR** (neat): 3395 (N–H), 3163 (C–H), 2950 (C–H), 1725 (C=O), 1674 (C=O), 1592 (C=C), 1475 (N–H), 1257 (C–O), 1168 (C–O), 1139 (C–O), 748 (C–F) cm^−1^; **HRMS** (ESI^+^): m/z calcd for C_19_H_12_FNO_5_Na^+^ [M+Na]^+^ 376.0597, found 376.0590.

#### 4-Bromo-***N***-(9-methoxy-7-oxo-7***H***-furo[3,2-***g***]chromen-4-yl)benzamide (3c)

The crude product was purified by silica gel column chromatography (eluent: DCM:MeOH = 100:0 to 97:3 v/v) to provide the title compound. Gray solid; 88% yield; **Mp**: 293–294 °C; **HPLC**: 98.12% purity; **TLC** (DCM:MeOH, 95:5 v/v): R_f_ = 0.42; ^**1**^**H NMR** (400 MHz, DMSO-*d*_*6*_): δ 10.56 (br s, 1H), 7.93 (d, *J* = 2.2 Hz, 1H), 7.90 (d, *J* = 9.8 Hz, 1H), 7.85 (d, *J* = 8.5 Hz, 2H), 7.62 (d, *J* = 8.5 Hz, 2H), 6.78 (d, *J* = 2.2 Hz, 1H), 6.28 (d, *J* = 9.8 Hz, 1H), 4.02 (s, 3H); ^**13**^**C NMR** (126 MHz, DMSO-*d*_*6*_): δ 166.0, 160.0, 147.8, 147.4, 143.1, 142.0, 137.8, 133.3, 132.0, 131.2, 130.6, 126.3, 124.1, 121.7, 114.3, 113.2, 106.8, 61.7; **IR** (neat): 3211 (N–H), 3149 (C–H), 2924 (C–H), 1734 (C=O), 1647 (C=O), 1590 (N–H), 1142 (C–O), 1116 (C–O), 752 (C–Br) cm^−1^; **HRMS** (ESI^+^): m/z calcd for C_19_H_12_BrNO_5_Na^+^ [M+Na]^+^ 437.9776, found 437.9762.

#### ***N***-(9-Methoxy-7-oxo-7***H***-furo[3,2-***g***]chromen-4-yl)-4-(trifluoromethyl)benzamide (3d)

The crude product was washed with hexanes to give the title compound. Off-white solid; 96% yield; **Mp**: 284–286 °C; **HPLC**: 96.30% purity; **TLC** (EtOAc:hexanes, 1:1): R_f_ = 0.50; ^**1**^**H NMR** (400 MHz, DMSO-*d*_*6*_): δ 10.78 (br s, 1H), 8.20 (d, *J* = 8.0 Hz, 2H), 8.08–8.00 (m, 2H), 7.88 (d, *J* = 8.1 Hz, 2H), 6.90 (d, *J* = 2.3 Hz, 1H), 6.38 (d, *J* = 9.9 Hz, 1H), 4.12 (s, 3H); ^**13**^**C NMR** (101 MHz, DMSO-*d*_*6*_): δ 165.4, 159.6, 147.6, 147.2, 142.9, 141.6, 137.8, 132.0 (q, *J* = 32.0 Hz), 131.0, 129.2, 125.7 (q, *J* = 4.1 Hz), 124.1 (q, *J* = 271.7 Hz), 123.9, 121.2, 114.1, 113.0, 106.5, 61.4; ^**19**^**F NMR** (376 MHz, DMSO-*d*_*6*_): δ –61.34 (s, 3F); **IR** (neat): 3230 (N–H), 3123 (C–H), 2945 (C–H), 1724 (C=O), 1652 (C=O), 1590 (C=C), 1521 (N–H), 1145 (C–O), 1108 (C–O), 824 (C–F) cm^−1^; **HRMS** (ESI^+^): m/z calcd for C_20_H_12_F_3_NO_5_Na^+^ [M+Na]^+^ 426.0565, found 426.0570.

#### 4-Methoxy-***N***-(9-methoxy-7-oxo-7***H***-furo[3,2-***g***]chromen-4-yl)benzamide (3e)

The crude product was purified by silica gel column chromatography (eluent: EtOAc:hexanes = 1:9–1:1) to provide the title compound. White solid; 31% yield; **Mp**: 211–212 °C; **HPLC**: 98.31% purity; **TLC** (EtOAc:hexanes, 4:1): R_f_ = 0.40; ^**1**^**H NMR** (500 MHz, DMSO-*d*_*6*_): δ 10.44 (br s, 1H), 8.05 (d, *J* = 2.3 Hz, 1H), 8.03 (d, *J* = 8.8 Hz, 2H), 7.99 (d, *J* = 9.9 Hz, 1H), 7.06 (d, *J* = 8.8 Hz, 2H), 6.88 (d, *J* = 2.1 Hz, 1H), 6.40 (d, *J* = 9.8 Hz, 1H), 4.15 (s, 3H), 3.82 (s, 3H); ^**13**^**C NMR** (126 MHz, DMSO-*d*_*6*_): δ 166.0, 162.7, 159.9, 147.6, 147.4, 143.1, 142.0, 130.9, 130.4, 126.1, 124.0, 122.3, 114.1, 114.0, 113.1, 106.8, 61.6, 55.9; **IR** (neat): 3214 (N–H), 3128 (C–H), 2950 (C–H), 1725 (C=O), 1644 (C=O), 1607 (C=C), 1592 (N–H), 1498 (C=C), 1478 (C=C), 1254 (C–O), 1023 (C–O) cm^−1^; **HRMS** (ESI^+^): m/z calcd for C_24_H_29_NO_6_Na^+^ [M+Na]^+^ 388.0797, found 388.0804.

#### ***N***-(9-Methoxy-7-oxo-***7H***-furo[3,2-***g***]chromen-4-yl)-3-nitrobenzamide (3f)

The crude product was purified by silica gel column chromatography (eluent: EtOAc:hexanes = 2:3–2:1) and then recrystallized with acetone to provide the title compound. Yellow solid; 33% yield; **Mp**: 267–268 °C; **HPLC**: 99.21% purity; **TLC** (EtOAc:hexanes, 1:2): R_f_ = 0.59; ^**1**^**H NMR** (500 MHz, DMSO-*d*_*6*_): δ 10.88 (br s, 1H), 8.81 (s, 1H), 8.42–8.35 (m, 2H), 8.04 (d, *J* = 9.9 Hz, 1H), 8.00 (d, *J* = 2.2 Hz, 1H), 7.77 (t, *J* = 8.0 Hz, 1H), 6.89 (d, *J* = 2.2 Hz, 1H), 6.35 (d, *J* = 9.9 Hz, 1H), 4.09 (s, 3H); ^**13**^**C NMR** (126 MHz, DMSO-*d*_*6*_): δ 164.8, 160.0, 148.3, 147.9, 147.4, 143.2, 142.0, 135.8, 135.0, 131.3, 130.8, 127.0, 124.1, 123.4, 121.4, 114.4, 113.2, 106.9, 61.7; **IR**: (neat): 3236 (N–H), 3117 (C–H), 1720 (C=O), 1660 (C=C), 1520 (N–O), 1350 (N–H) cm^−1^; **HRMS** (ESI^+^): m/z calcd for C_19_H_12_N_2_O_7_H^+^ [M+H]^+^ 381.0767, found 381.0817.

#### ***N***-(9-Methoxy-7-oxo-7***H***-furo[3,2-***g***]chromen-4-yl)furan-2-carboxamide (3g)

The crude product was purified by silica gel column chromatography (eluent: DCM:MeOH = 100:0 to 99:1 v/v) to provide the title compound. Yellow-green solid; 39% yield; **Mp**: 227–228 °C; **HPLC**: 99.45% purity; **TLC** (DCM:MeOH, 95:5 v/v): R_f_ = 0.64; ^**1**^**H NMR** (500 MHz, DMSO-*d*_*6*_): δ 10.52 (br s, 1H), 7.97 (d, *J* = 2.3 Hz, 1H), 7.93–7.85 (m, 2H), 7.28 (d, *J* = 3.4 Hz, 1H), 6.79 (d, *J* = 2.3 Hz, 1H), 6.63 (dd, *J* = 3.6, 1.8 Hz, 1H), 6.32 (d, *J* = 9.8 Hz, 1H), 4.06 (s, 3H); ^**13**^**C NMR** (126 MHz, DMSO-*d*_*6*_): δ 160.0, 157.5, 147.8, 147.4, 147.4, 146.5, 143.1, 141.9, 131.2, 124.2, 121.0, 115.9, 114.2, 113.3, 112.7, 106.8, 61.6; **IR** (neat): 3257 (N–H), 3142 (C–H), 3065 (C=C), 2955 (C–H), 1720 (C=O), 1653 (C=O), 1590 (C=C), 1466 (N–H), 1423 (C=C), 1137 (C–O), 1170 (C–O) cm^−1^; **HRMS** (ESI^+^): m/z calcd for C_17_H_11_NO_6_Na^+^ [M+Na]^+^ 348.0484, found 348.0484.

#### ***N***-(9-Methoxy-7-oxo-7***H***-furo[3,2-***g***]chromen-4-yl) thiophene-2-carboxamide (3h)

The crude product was purified by silica gel column chromatography (eluent: 100%hexanes to EtOAc:hexanes = 1:3) to provide the title compound. Green solid; 49% yield; **Mp**: 213–214 °C; **HPLC**: 98.42% purity; **TLC** (DCM:MeOH, 95:5 v/v): R_f_ = 0.50; ^**1**^**H NMR** (500 MHz, DMSO-*d*_*6*_): δ 10.63 (br s, 1H), 8.09 (d, *J* = 3.3 Hz, 1H), 8.08 (d, *J* = 2.2 Hz, 1H), 8.01 (d, *J* = 9.8 Hz, 1H), 7.88 (dd, *J* = 5.0, 0.9 Hz, 1H), 7.24 (dd, *J* = 4.9, 3.8 Hz, 1H), 6.90 (d, *J* = 2.2 Hz, 1H), 6.42 (d, *J* = 9.9 Hz, 1H), 4.16 (s, 3H); ^**13**^**C NMR** (126 MHz, DMSO-*d*_*6*_): δ 161.1, 159.8, 147.8, 147.3, 143.0, 141.8, 139.1, 132.6, 131.1, 130.4, 128.6, 124.0, 121.2, 114.3, 113.1, 106.7, 61.6; **IR** (neat): 3236 (N–H), 3123 (C–H), 2921 (C–H), 1725 (C=O), 1635 (C=O), 1590 (C=C), 1525 (N–H), 1414 (C=C), 1140 (C–O), 1098 (C–O), 718 (C–S) cm^−1^; **HRMS** (ESI^+^): m/z calcd for C_17_H_11_NO_5_SNa^+^ [M+Na]^+^ 364.0256, found 364.0261.

#### ***N***-(9-Methoxy-7-oxo-7***H***-furo[3,2-***g***]chromen-4-yl)acetamide (3i)

The title compound was synthesized following a slightly modified literature procedure^37^ using **2** (24.0 mg, 0.10 mmol, 1.0 equiv.) in DCM (2.0 mL), a solution of acetic anhydride in DCM (1.00 mL, 1.0 mmol, 10.0 equiv.), pyridine (16 µL, 0.20 mmol, 2.0 equiv.) and DMAP (2.5 mg, 0.02 mmol, 0.2 equiv.), and K_2_CO_3_ (27.6 mg, 0.20 mmol, 2.0 equiv.). The crude product was purified by recrystallization with EtOH to provide the title compound. White solid; 42% yield; **Mp**: 251–252 °C; **HPLC**: 99.61% purity; **TLC** (DCM:MeOH, 95:5 v/v): R_f_ = 0.67; ^**1**^**H NMR** (500 MHz, DMSO-*d*_*6*_): δ 10.06 (br s, 1H), 7.95 (d, *J* = 2.2 Hz, 1H), 7.91 (d, *J* = 9.9 Hz, 1H), 6.79 (d, *J* = 2.2 Hz, 1H), 6.32 (d, *J* = 9.8 Hz, 1H), 4.03 (s, 3H), 2.05 (s, 3H); ^**13**^**C NMR** (126 MHz, DMSO-*d*_*6*_): δ 169.5, 159.8, 147.3, 147.3, 142.9, 141.8, 130.6, 123.2, 122.0, 113.7, 112.2, 106.7, 61.4, 23.2; **IR** (neat): 3324 (N–H), 3114 (C–H), 2955 (C–H), 1709 (C=O), 1687 (C=O), 1593 (C=C), 1506 (N–H), 1163 (C–O), 1134 (C–O), 1058 (C–O) cm^−1^; **HRMS** (ESI^+^): m/z calcd for C_14_H_11_NO_5_Na^+^ [M+Na]^+^ 296.0535, found 296.0533.

#### ***N***-(9-Methoxy-7-oxo-7***H***-furo[3,2-***g***]chromen-4-yl)propionamide (3j)

The crude product was washed with hexanes to give the title compound. Gray solid; 64% yield; **Mp**: 233–234 °C; **HPLC**: 98.25% purity; **TLC** (DCM:MeOH, 95:5 v/v): R_f_ = 0.75; ^**1**^**H NMR** (500 MHz, DMSO-*d*_*6*_): δ 10.06 (br s, 1H), 8.03 (s, 1H), 7.96 (d, *J* = 9.8 Hz, 1H), 6.83 (s, 1H), 6.40 (d, *J* = 9.8 Hz, 1H), 4.10 (s, 3H), 2.42 (q, *J* = 7.4 Hz, 2H), 1.12 (t, *J* = 7.4 Hz, 3H); ^**13**^**C NMR** (126 MHz, DMSO-*d*_*6*_): δ 173.2, 159.8, 147.3, 147.2, 142.9, 141.7, 130.5, 123.1, 122.0, 113.7, 112.2, 106.6, 61.4, 28.9, 10.0; **IR** (neat): 3243 (N–H), 3118 (C–H), 2980 (C–H), 1719 (C=O), 1655 (C=O), 1591 (C=C), 1515 (N–H), 1425 (C=C), 1162 (C–O), 1135 (C–O) cm^−1^; **HRMS** (ESI^+^): m/z calcd for C_15_H_13_NO_5_Na^+^ [M+Na]^+^ 310.0691, found 310.0683.

#### ***N***-(9-Methoxy-7-oxo-7***H***-furo[3,2-***g***]chromen-4-yl)isobutyramide (3k)

The crude product was purified by silica gel column chromatography (eluent: EtOAc:hexanes = 1:3–1:1) to provide the title compound. White solid; 25% yield; **Mp**: 234–235 °C; **HPLC**: 99.19% purity; **TLC** (EtOAc:hexanes, 4:1): R_f_ = 0.53; ^**1**^**H NMR** (500 MHz, CDCl_3_): δ 7.70 (d, *J* = 9.8 Hz, 1H), 7.62 (d, *J* = 2.2 Hz, 1H), 7.50 (br s, 1H), 6.64 (d, *J* = 2.2 Hz, 1H), 6.31 (d, *J* = 9.7 Hz, 1H), 4.25 (s, 3H), 2.79–2.67 (m, 1H), 1.35 (d, *J* = 6.9 Hz, 6H); ^**13**^**C NMR** (126 MHz, CDCl_3_): δ 176.4, 159.9, 147.3, 146.3, 142.9, 139.5, 131.8, 123.5, 118.8, 114.5, 112.8, 104.9, 61.3, 35.8, 19.7; **IR** (neat): 3248 (N–H), 3167 (C–H), 3129 (C=C), 2971 (C–H), 1732 (C=O), 1650 (C=O), 1588 (C=C), 1518 (N–H), 1161 (C–O), 1142 (C–O), 1128 (C–O) cm^−1^; **HRMS** (ESI^+^): m/z calcd for C_16_H_15_NO_5_Na^+^ [M+Na]^+^ 324.0848, found 324.0843.

#### ***N***-(9-Methoxy-7-oxo-7***H***-furo[3,2-***g***]chromen-4-yl)pivalamide (3l)

The crude product was purified by recrystallization with EtOH to provide the title compound. Yellow solid; 32% yield; **Mp**: 210–212 °C; **HPLC**: 97.01% purity; **TLC** (DCM:MeOH, 95:5 v/v): R_f_ = 0.40; ^**1**^**H NMR** (500 MHz, DMSO-*d*_*6*_): δ 9.65 (br s, 1H), 8.05 (s, 1H), 7.83 (d, *J* = 9.3 Hz, 1H), 6.74 (s, 1H), 6.42 (d, *J* = 9.3 Hz, 1H), 4.12 (s, 3H), 1.28 (s, 9H); ^**13**^**C NMR** (126 MHz, DMSO-*d*_*6*_): δ 178.0, 159.9, 147.6, 147.3, 142.9, 141.6, 130.8, 124.1, 122.4, 114.1, 113.2, 106.5, 61.5, 31.1, 27.7; **IR** (neat): 3215 (N–H), 3134 (C–H), 2984 (C–H), 1730 (C=O), 1640 (C=O), 1590 (C=C), 1505 (N–H), 1475 (C=C), 1376 (C–C), 1154 (C–O), 1133 (C–O) cm^−1^; **HRMS** (ESI^+^): m/z calcd for C_17_H_17_NO_5_Na^+^ [M+Na]^+^ 338.1004, found 338.1004.

#### ***N-H***exanoyl-***N***-(9-methoxy-7-oxo-7***H***-furo[3,2-***g***]chromen-4-yl)hexanamide (3m)

The crude product was purified by silica gel column chromatography (eluent: EtOAc:hexanes = 1:9–1:2) to provide the title compound. White solid; 21% yield; **Mp**: 170–171 °C; **HPLC**: 97.31% purity; **TLC** (DCM:MeOH, 95:5 v/v): R_f_ = 0.67; ^**1**^**H NMR** (500 MHz, CDCl_3_): δ 7.71 (d, *J* = 2.3 Hz, 1H), 7.54 (d, *J* = 9.8 Hz, 1H), 6.61 (d, *J* = 2.3 Hz, 1H), 6.44 (d, *J* = 9.9 Hz, 1H), 4.35 (s, 3H), 2.57–2.50 (m, 4H), 1.67–1.57 (m, 4H), 1.31–1.16 (m, 8H), 0.84 (t, *J* = 7.0 Hz, 6H); ^**13**^**C NMR** (126 MHz, CDCl_3_): δ 175.7, 159.3, 147.7, 147.0, 143.0, 137.9, 133.3, 125.5, 120.4, 116.6, 114.7, 103.8, 61.3, 38.0, 31.1, 24.2, 22.3, 13.7; **IR** (neat): 3441 (N–H), 3126 (C–H), 2953 (C–H), 2926 (C–H), 2858 (C–H), 1726 (C=O), 1685 (C=O), 1589 (C=C), 1480 (N–H), 1163 (C–O), 1135 (C–O) cm^−1^; **HRMS** (ESI^+^): m/z calcd for C_24_H_31_NO_5_Na^+^ [M+Na]^+^ 450.1893, found 450.1885.

#### (3***r***,5***r***,7***r***)-***N***-(9-Methoxy-7-oxo-7***H***-furo[3,2-***g***]chromen-4-yl)adamantane-1-carboxamide (3n)

The crude product was purified by silica gel column chromatography (eluent: 100%hexanes to EtOAc:hexanes = 1:4) to provide the title compound. Off-white solid; 15% yield; **Mp**: 240–242 °C; **HPLC**: 97.53% purity; **TLC** (EtOAc:hexanes, 2:1): R_f_ = 0.48; ^**1**^**H NMR** (500 MHz, CDCl_3_): δ 7.62 (br s, 1H), 7.59 (d, *J* = 9.8 Hz, 1H), 7.58 (d, *J* = 2.2 Hz, 1H), 6.55 (d, *J* = 2.2 Hz, 1H), 6.23 (d, *J* = 9.8 Hz, 1H), 4.22 (s, 3H), 2.13 (s, 3H), 2.04 (d, *J* = 2.5 Hz, 6H), 1.79 (q, *J* = 12.5 Hz, 6H); ^**13**^**C NMR** (126 MHz, CDCl_3_): δ 177.6, 160.1, 147.4, 146.4, 143.0, 139.8, 131.7, 123.8, 119.5, 114.4, 113.0, 105.2, 61.5, 41.6, 39.5, 36.5, 28.2; **IR**: (neat): 2953 (C–H), 1729 (C=O), 1640 (C=C), 1590 (C=C), 1133 (C–O) cm^−1^; **HRMS** (ESI^+^): m/z calcd for C_23_H_23_NO_5_Na^+^ [M+Na]^+^ 416.1468, found 416.1462; Data are consistent with literature values^15^.

#### 4-(Benzylamino)-9-methoxy-7H-furo[3,2-g]chromen-7-one (4a)

The title compound was synthesized following a slightly modified literature procedure^15^ using **2** (72.0 mg, 0.30 mmol, 1.0 equiv.) in anhydrous acetone (1.5 mL) benzyl bromide (43 µL, 0.36 mmol, 1.2 equiv.) and K_2_CO_3_ (63.0 mg, 0.45 mmol, 1.5 equiv.) at 55 °C overnight. The crude product was purified by silica gel column chromatography (eluent: 100%hexanes to EtOAc:hexanes = 2:3) to provide the title compound. Yellow solid; 21% yield; **Mp**: 168–169 °C; **HPLC**: 97.00% purity; **TLC** (EtOAc:hexanes, 1:1): R_f_ = 0.50; ^**1**^**H NMR** (400 MHz, CDCl_3_): δ 7.84 (d, *J* = 9.9 Hz, 1H), 7.53 (d, *J* = 2.3 Hz, 1H), 7.37–7.34 (m, 5H), 6.82 (d, *J* = 2.3 Hz, 1H), 6.18 (d, *J* = 9.9 Hz, 1H), 4.65 (s, 2H), 4.11 (s, 3H); ^**13**^**C NMR** (101 MHz, CDCl_3_): δ 160.6, 150.3, 144.7, 144.4, 138.8, 134.4, 129.0, 127.9, 127.5, 126.5, 114.3, 112.9, 111.2, 105.9, 104.6, 61.7, 52.5; **IR**: (neat): 3421 (N–H), 3121 (C–H), 2932 (C–H), 1703 (C=O), 1585 (C=C), 1152 (C–O) cm^−1^; **HRMS** (ESI^+^): m/z calcd for C_19_H_15_NO_4_H^+^ [M+H]^+^ 322.1080, found 322.1078; Data are consistent with literature values^15^.

#### 4-((4-Bromobenzyl)amino)-9-methoxy-***7H***-furo[3,2-***g***]chromen-7-one (4b)

The title compound was synthesized with the same protocol as **4a** using 4-bromobenzyl bromide (48 µL, 0.36 mmol, 1.2 equiv.)^15^. The crude product was purified by silica gel column chromatography (eluent: EtOAc:hexanes = 1:9–2:1) to provide the title compound. Yellow solid; 39% yield; **Mp**: 150–151 °C; **HPLC**: 97.78% purity; **TLC** (EtOAc:hexanes, 1:1): R_f_ = 0.60; ^**1**^**H NMR** (500 MHz, CDCl_3_): δ 7.85 (d, *J* = 9.9 Hz, 1H), 7.53 (d, *J* = 2.3 Hz, 1H), 7.48 (d, *J* = 8.3 Hz, 2H), 7.23 (d, *J* = 8.2 Hz, 2H), 6.75 (d, *J* = 2.3 Hz, 1H), 6.18 (d, *J* = 9.8 Hz, 1H), 4.60 (s, 2H), 4.11 (s, 3H); ^**13**^**C NMR** (126 MHz, CDCl_3_): δ 160.7, 150.3, 144.8, 144.6, 138.9, 138.0, 134.2, 132.1, 129.2, 126.6, 121.8, 114.4, 111.4, 105.9, 104.8, 61.8, 51.7; **IR**: (neat): 3411 (N–H), 1691 (C=O), 1604 (C=C), 1530 (C=C), 716 (C–Br) cm^−1^; **HRMS** (ESI^+^): m/z calcd for C_19_H_14_BrNO_4_H^+^ [M+H]^+^ 400.0179, found 400.0169.

#### ***N***-(9-Methoxy-7-oxo-7***H***-furo[3,2-***g***]chromen-4-yl)benzenesulfonamide (5)

The title compound was synthesized following a slightly modified literature procedure^18^ using **2** (47.0 mg, 0.20 mmol) in pyridine (400 µL); benzenesulfonyl chloride (30 µL, 0.22 mmol) and DMAP (2.5 mg, 0.02 mmol). The crude product was purified by silica gel column chromatography (eluent: EtOAc:hexanes = 1:4–3:1) to provide the title compound. Dark-green solid; 82% yield; **Mp**: 208–209 °C; **HPLC**: 98.02% purity; **TLC** (EtOAc:hexanes, 4:1): R_f_ = 0.53; ^**1**^**H NMR** (300 MHz, acetone-*d*_*6*_): δ 9.07 (br s, 1H), 8.05 (d, *J* = 9.9 Hz, 1H), 7.70 (d, *J* = 2.3 Hz, 1H), 7.65–7.61 (m, 2H), 7.45 (t, *J* = 7.7 Hz, 3H), 6.35 (d, *J* = 2.3 Hz, 1H), 6.26 (d, *J* = 9.9 Hz, 1H), 4.18 (s, 3H); ^**13**^**C NMR** (75 MHz, acetone-*d*_*6*_): δ 160.2, 148.3, 148.0, 144.6, 142.0, 140.4, 134.3, 133.4, 130.4, 128.4, 126.6, 120.1, 116.5, 115.1, 106.3, 61.9; **IR** (neat): 3201 (N–H), 3160 (C–H), 2951 (C–H), 1697 (C=O), 1615 (C=O), 1588 (C=C), 1474 (C=C), 1338 (S=O), 1164 (C–O), 1130 (C–O), 1053 (C–O), 724 (C–S) cm^−1^; **HRMS** (ESI^+^): m/z calcd for C_18_H_13_NO_6_SNa^+^ [M+Na]^+^ 394.0362, found 394.0365.

#### 1-(5,9-Dimethoxy-7-oxo-6,7-dihydro-5***H***-furo[3,2-***g***]chromen-4-yl)-3-phenylthiourea (6)

The title compound was synthesized following a slightly modified literature procedure^19^ using **2** (47.0 mg, 0.20 mmol, 1.0 equiv.) in MeOH (1.0 mL), phenylisothiocyanate (90 µL, 2.22 mmol, 3.1 equiv.) under Ar atmosphere at 65 °C overnight. The crude product was purified by silica gel column chromatography (eluent: EtOAc:hexanes = 1:9–1:1) to provide the title compound. Dark-green solid; 49% yield; **Mp**: 191–192 °C; **HPLC**: 98.42% purity; **TLC** (EtOAc:hexanes, 2:3): R_f_ = 0.74; ^**1**^**H NMR** (400 MHz, CDCl_3_): δ 8.82 (br s, 1H), 7.52 (d, *J* = 2.0 Hz, 1H), 7.49–7.43 (m, 4H), 7.39 (t, *J* = 6.5 Hz, 1H), 6.89 (d, *J* = 1.9 Hz, 1H), 6.23 (br s, 1H), 5.50 (t, *J* = 5.0 Hz, 1H), 4.15 (s, 4H), 3.55 (s, 4H), 3.00–2.74 (m, 2H); ^**13**^**C NMR** (101 MHz, CDCl_3_): δ 176.4, 170.2, 146.0, 144.0, 143.6, 140.4, 129.2, 128.6, 128.4, 128.1, 122.0, 108.6, 103.2, 103.0, 60.9, 57.2, 51.9, 38.4; **IR**: (neat): 3274 (N–H), 2940 (C–H), 1744 (C=O), 1721 (C=O), 1644 (C=C), 1477 (C–H), 1342 (S=O), 1068 (S=O), 1045 (S=O) cm^−1^; **HRMS** (ESI^+^): m/z calcd for C_20_H_18_N_2_O_5_SH^+^ [M+H]^+^ 399.1015, found 399.1011.

### Anti-cancer activity

#### Cell lines

MDA-MB-231 (triple-negative breast cancer) was obtained from M.D. Anderson Cancer Center, Houston, TX, USA). T47-D (hormone-dependent breast carcinoma), SK-BR-3 (human HER2+ breast cancer), and MRC-5 (normal embryonic lung fibroblast) were obtained from the American Type Culture Collection (ATCC, Manassas, VA, USA).

#### Cytotoxicity evaluation

The cytotoxic effect of tested compounds was evaluated in two breast cancer cell lines, MDA-MB-231 and T47-D. Number of survival cells after exposure to tested compounds for 48 h was determined by MTT assay, as previously described^38^. The cancer selectivity of the tested compounds was demonstrated by comparing with cytotoxicity of a normal cell line, MRC-5. Three standard anti-cancer drugs, doxorubicin, tamoxifen citrate, and lapatinib, were used as positive controls in this assay.

### Cancer chemopreventive activities

#### Inhibition of aromatase (AIA)

Inhibition of the aromatase was performed using the method designed by Stresser et al.^39^. The reference compound, letrozole, inhibited CYP19 with an IC_50_ value of 2.6 ± 0.1 nM.

For other cancer chemoprevention assays please see Supplementary.

### Phototoxicity

SK-BR-3 and MDA-MB-231 cells were seeded into 96-well cell culture plates at 5 × 10^3^ cells/well and incubated for 24 h at 37 °C under 5% CO_2_. After that, the cells were treated with 50 μM of psoralen derivatives in DMEM with 10% FBS and the cells were continued incubation for another 4 h. After incubation, the cells were washed with PBS (3 times) and then irradiated by a 365 nm lamp with the light intensity of 2.0 J cm^−2^ for 12.5 min before re-incubation for another 72 h. After incubation, the cell was treated with methylthiazolyldiphenyl-tetrazolium bromide (MTT reagent 200 μL, 0.5 mg mL^−1^, Sigma-Aldrich) for 2 h. After reagent removal, DMSO was added to dissolve the formazan product and the cell viabilities were determined through UV–Vis absorption of the resulting formazan at wavelength 560 nm using a microplate reader (BMG Labtech/SPECTROstar Nano). The cell morphology images were captured by using a light microscope (Microscope Nikon, inverted digital) at 20X magnification prior to treating with MTT reagent.

### Molecular docking studies

The protein HER2 co-crystalized with a small molecule inhibitor (PDB ID: 3PP0) was retrieved from the protein data bank (https://www.rcsb.org/PDB). Both protein and ligands were prepared using AutoDock Tools 1.5.7^40^. The ligands and solvent molecules were removed, and polar hydrogen atoms were added. Tautomeric forms of histidine residues were set in accordance with the PQR computed with AMBER forcefield at pH 7.0 using APBS-PDB2PQR software suite (https://server.poissonboltzmann.org/)^41^. Two docking grid boxes were set as: box 1, center at (10,29,27) of size x = 28 Å, y = 40 Å, z = 20 Å, and box 2^11^, center at (9,24.5,42) of size x = 28 Å, y = 48 Å, z = 44 Å. Both of the grid boxes gave similar results with no significant difference in most cases (see Supplementary Table S2). The exhaustiveness of conformational search is set to 16.

Molecular docking was performed using AutoDock Vina 1.1.2^40^. The models were validated by redocking the co-crystalized ligand back into the binding site. The root mean-squared deviation (RMSD) between the heavy atoms is calculated using obrms as implemented in openbabel^42,43^. The top scoring predicted pose of the co-crystalized ligand has an RMSD of 0.60 Å (box 1) and 0.62 Å (box 2) compared with the X-ray pose. Docked poses were analyzed and figures were prepared using Obabel 3.1.1, Maestro 13.0, and PyMol 1.8^44,45^.

### The physicochemical properties

Drug-likeness properties and Lipinski's rule of five were obtained by using SwissADME website services^46,47^.

## Conclusions

Twenty psoralen derivatives with C-5 substitution were successfully synthesized, of which sixteen compounds were novel. Although the dark IC_50_ towards breast cancer cells of these derivatives were relatively high, possibly due to the unusually low sensitivity of the cancer cell lines, some important structural features at C-5 can be deduced. In addition, these derivatives showed strong selectivity towards hormone-dependent T47-D compared to the hormone-independent MDA-MB-231, and low toxicity towards normal cells. Moreover, significant cancer chemopreventive activity through aromatase inhibition was also observed in some compounds. The presence of UVA irradiation led to a dramatic increase in the cytotoxicity, and the aliphatic and heteroaromatic amides were the most active with the IC_50_ of up to 2.71 µM for **3g**. The extreme selectivity of the phototoxicity towards the HER2+ SK-BR-3 over the HER2– MDA-MB-231 suggested the HER2 as the possible target, which was further confirmed by the molecular docking study. Additionally, the cell morphology suggested that the significant inhibition of SK-BR-3 cells under UVA irradiation appeared to be related to induction of cell apoptosis. Finally, the physicochemical properties for most of the synthesized compounds are in the acceptable range for drug-likeness. The knowledge obtained from this work is crucial for the further development of anti-breast cancer agents based on the psoralen scaffold in the future.

## Supplementary Information

Supplementary Information.

## Data Availability

The datasets generated and/or analyzed during the current study are available in the supplementary file. The top scoring molecular docking poses of 8-MOP, **3g**, and **3j** overlaid on HER2 protein structure (PDB ID: 3PP0, retrieved from the protein data bank, https://www.rcsb.org/structure/3pp0) are available in the open access repository, https://github.com/ruchuta/Molecular_Docking.
