# Supplementary material for: Novel psoralen derivatives as anti-breast cancer agents and their light-activated cytotoxicity against HER2 positive breast cancer cells
Source: Sci Rep. 2022 Aug 5;12:13487. doi: 10.1038/s41598-022-17625-x (PMC9356065; doi:10.1038/s41598-022-17625-x)
Supplement: Supplementary file 1 — Supplementary Information. [file 41598_2022_17625_MOESM1_ESM.pdf]

Supporting Information

Novel Psoralen Derivatives as Anti-Breast Cancer Agents and their Light-Activated Cytotoxicity against HER2 Positive Breast Cancer Cells

Chiphada Aekrungrueangkit[1], Sirilak Wangngae[2], Anyanee Kamkaew[2], Ruchuta Ardkhean[3], Sanit Thongnest[4,5], Jutatip Boonsombat[4,5,*], Somsak Ruchirawat[4,5,6], Tanatorn Khotavivattana[1,*]

[1] *Center of Excellence in Natural Products Chemistry, Department of Chemistry, Faculty of Science, Chulalongkorn University, Bangkok, 10330, Thailand*

[2] *School of Chemistry, Institute of Science, Suranaree University of Technology, Nakhon, Ratchasima, 30000, Thailand.*

[3] *Princess Srisavangavadhana College of Medicine, Chulabhorn Royal Academy, Bangkok, 10210, Thailand*

[4] *Chulabhorn Research Institute, Bangkok, 10330, Thailand*

[5] *Center of Excellence on Environmental Health and Toxicology (EHT), OPS, MHESI, Thailand*

[6] *Program in Chemical Sciences, Chulabhorn Graduate Institute, Chulabhorn Royal Academy, Bangkok 10210, Thailand*

***Corresponding authors** *jutatip@cri.or.th and tanatorn.k@chula.ac.th*

Content

1. Chemoprevention activity S1
   1.1 Scavenging of diphenyl-picryl-hydrazyl radicals (DPPH) S1
   1.2 Inhibition of 12-*O*-tetradecanoylphorbol-13-acetate (TPA)-induced superoxide anion radical generation in differentiated HL-60 cells (HL-60 Antioxidant) S1
   1.3 Inhibition of superoxide radical formation by xanthine/xanthine oxidase (XXO) S1
   1.4 Inhibition of xanthine oxidase (IXO) S1
   1.5 Inhibition of lipoxygenase (LOX) S1
2. Molecular docking results of selected compounds S3
3. Light-activated cytotoxicity of psoralen derivatives against SK-BR-3 .... S4
4. Cell Viability of the untreated SK-BR-3 and MDA-MB-231 cells under dark and light irradiation S7
4. NMR and HRMS spectra S8
5. HPLC spectra S49
6. References S69

1. Chemoprevention activity

1.1. Scavenging of diphenyl-picryl-hydrazyl radicals (DPPH)

Radical scavenging potential was performed by reaction with 1,1-diphenyl-2-picrylhydrazyl (DPPH) free radicals as described by van Amsterdam et al., 1992.[i] Ascorbic acid was used as the reference compound, showing a half-maximal scavenging concentration (IC_{50}) at 43.1 ± 1.3 µM (**Table S1**).

1.2. Inhibition of 12-*O*-tetradecanoylphorbol-13-acetate (TPA)-induced superoxide anion radical generation in differentiated HL-60 cells (HL-60 Antioxidant)

TPA-induced superoxide anion radical formation was detected in differentiated HL-60 cells by photometric determination of cytochrome c reduction as previously described by Gerhäuser et al., 2003.[ii] Superoxide dismutase (SOD;15U) was a positive control. Only the test compounds with >50% cell viability were considered to calculate scavenging potential (**Table S1**).

1.3. Inhibition of superoxide radical formation by xanthine/xanthine oxidase (XXO)

Inhibition of superoxide radical formation was detected indirectly by measuring the rate of reduced XTT production as described by Gerhäuser et al, 2003.[iii] Gallic acid was a positive control, exhibiting an IC_{50} value of 2.8 ± 0.1 µM. Inhibition of superoxide radical formation was determined only when the tested compounds did not inhibit xanthine oxidase (**Table S1**).

1.4. Inhibition of xanthine oxidase (IXO)

Xanthine oxidase activity was determined by quantifying the amount of uric acid produced from xanthine. The method was followed as described by Rangkadilok et al., 2007.[iii] Allopurinol was used as a positive control, inhibiting xanthine oxidase activity with an IC_{50} value of 3.8 ± 0.4 µM (**Table S1**).

1.5. Inhibition of lipoxygenase (LOX)

Lipoxygenase activity was performed by measuring leukotriene metabolite as described by Gleason et al., 1995.[iv] Nordihydroquaiaretic acid, a positive compound, inhibited lipoxygenase activity with an IC_{50} value of 3.8 ± 0.6 µM (**Table S1**).

Table S1 Chemoprevention evaluation of psoralen derivatives.

Comp. 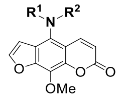			DPPH		HL-60 Antiox.		XXO		IXO		LOX	
	R^1	R^2	%Inh	IC_{50} [μM]	%Inh	IC_{50} [μM]	%Inh	IC_{50} [μM]	%Inh	IC_{50} [μM]	%Inh	IC_{50} [μM]
**8-MOP**		-H	1	-	17	-	37	-	-	183.7 ± 5.4	9	-
**1**		$-NO_2$	5	-	1	-	IX	-	HBg, HOD	-	4	-
**2**		$-NH_2$	23	-	0	-	IX	-	HBg, HOD	-	3	-
**3a**	H	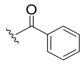	6	-	3	-	IX	-	HBg, HOD	-	0	-
**3b**	H	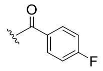	3	-	1	-	IX	-	HBg, HOD	-	3	-
**3c**	H	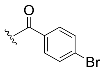	6	-	Toxic	-	25	-	HBg, HOD	-	4	-
**3d**	H	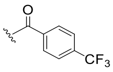	7	-	Toxic	-	IX	-	HBg, HOD	-	2	-
**3e**	H	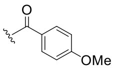	4	-	0	-	IX	-	HBg, HOD	-	0	-
**3f**	H	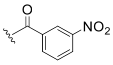	1	-	7	-	33	-	HOD	-	0	-
**3g**	H	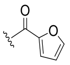	3	-	17	-	IX	-	HOD	-	3	-
**3h**	H	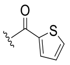	6	-	24	-	IX	-	HOD	-	5	-
**3i**	H	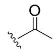	1	-	11	-	IX	-	47	-	4	-
**3j**	H	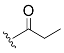	2	-	11	-	-	187.3 ± 23.3	29	-	6	-
**3k**	H	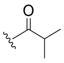	2	-	11	-	-	394.4 ± 65.8	17	-	0	-
**3l**	H	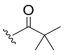	1	-	10	-	IX	-	HOD	-	6	-
**3m**	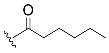	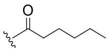	1	-	0	-	2	-	HOD	-	-	56.1 ± 0.5
**3n**	H	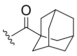	1	-	8	-	47	-	HBg, HOD	-	6	-
**4a**	H	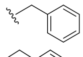	-	145.0 ± 9.6	7	-	34	-	-	27.4 ± 5.9	30	-
**4b**	H	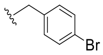	-	165.7 ± 1.1	Toxic	-	HBg	-	HBg	-	9	-
**5**	H	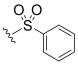	8	-	32	-	-	90.7 ± 6.2	21%	-	2	-
**6**	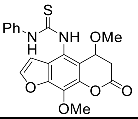		-	52.6 ± 3.1	Toxic	-	24	-	48, HOD	-	16	-
**Screening Concentration (final conc.)**			250 μM		100 μM		500 μM		500 μM		100 μM	
**Standard**			Ascorbic acid 43.1 ± 1.3 μM		Superoxide dismutase		Gallic acid 2.8 ± 0.1 μM		Allopurinol 3.8 ± 0.4 μM		Nordihydroquaiaretic acid 3.8 ± 0.6 μM	

%Inhibition at tested concentration, - = not determined, HBg = high background, HOD = High optical density (absorbance > 1), IX = Inhibit xanthine oxidase, Toxic = tested compound was toxic with cell (cannot detect the generated radical).

2. Molecular docking results of selected compounds

Table S2 Docking scores, protein residues with hydrogen-bond, halogen bond, Pi-cation, Pi-Pi stacking or hydrophobic interactions with the ligands, and phototoxicity of selected compounds.

**Comp.**	**SK-BR-3[a] with UVA light (2.0 J/cm²)**	**HER2 (PDB ID: 3PP0)**		
	**IC_{50} (μM)**	**RMSD[b] (Å)**	**Docking scores[c] (kcal/mol)**	**Protein residues[c]**
**8MOP**	24.66 ± 3.02	4.65	-7.9	-
**3e**	66.72 ± 2.75	0.06	-7.2	ARG849, PHE731
**3g**	2.71 ± 0.84	0.51	-9.4	MET801
**3j**	3.05 ± 1.02	0.04	-8.3	MET801, ASP863
**3k**	66.09 ± 5.21	0.49	-8.4	MET801
**3l**	48.14 ± 3.07	0.03	-8.5	MET801
**3m**	27.09 ± 1.20	0.22	-6.0	ARG849
**03Q[d]**	-	0.07	-11.5	MET801, Lys753, Leu796

[a]HER2 mammary carcinoma; [b]RMSD between docked poses obtained from two grid boxes [box1; center at (10,29,27), x = 28 Å, y = 40 Å, z = 20 Å, and box 2; center at (9,24.5,42) x = 28 Å, y = 48 Å, z = 44 Å]; [c]Box 1, [d]Co-crystallized ligand of 3PP0.

3. Light-activated cytotoxicity of psoralen derivatives against SK-BR-3

Light-activated cytotoxicity of psoralen derivatives against SK-BR-3 in the different concentration under irradiation with UVA (2 J/cm^2) for 12.5 min.

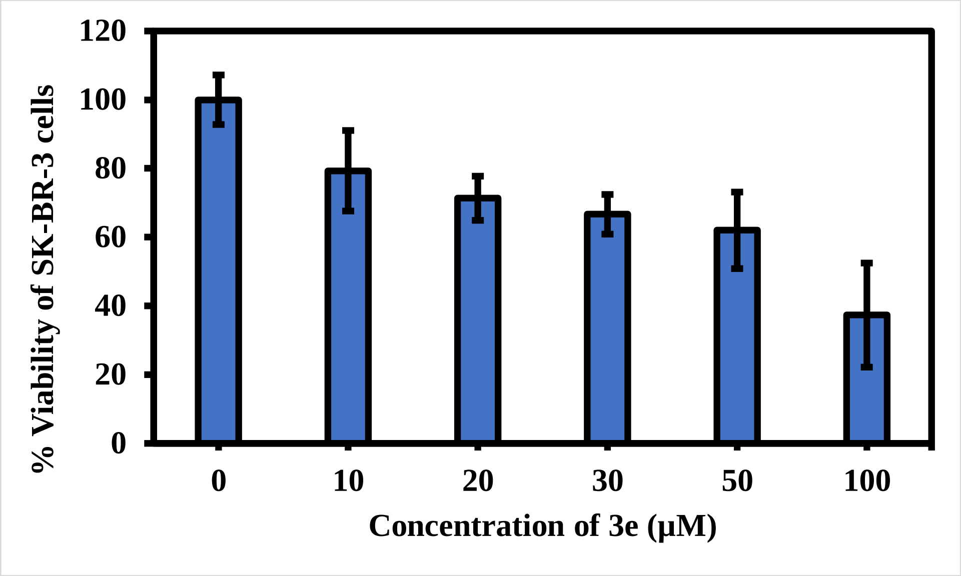

Figure S1 The IC_{50} determination of **3e**

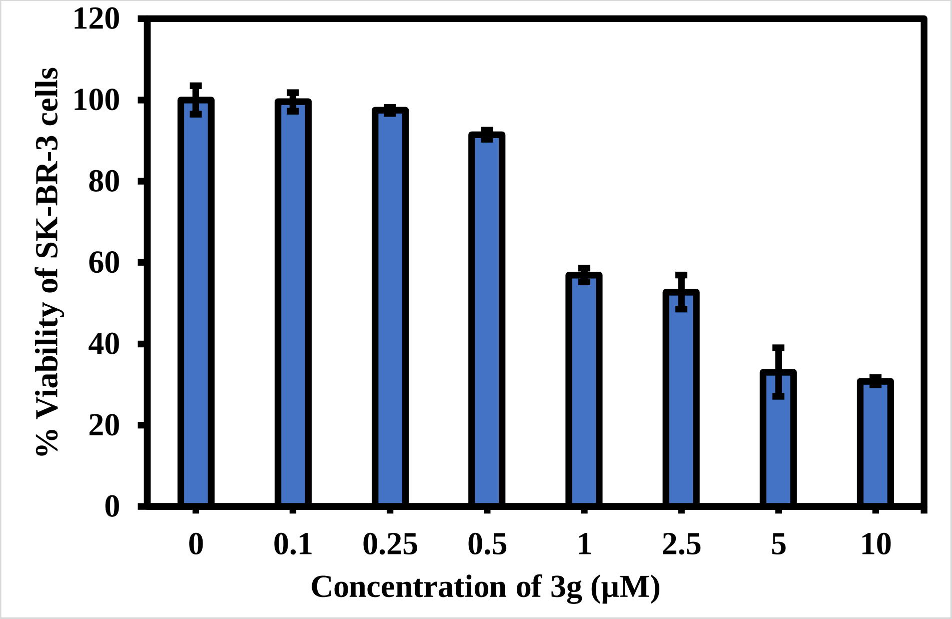

Figure S2 The IC_{50} determination of **3g**

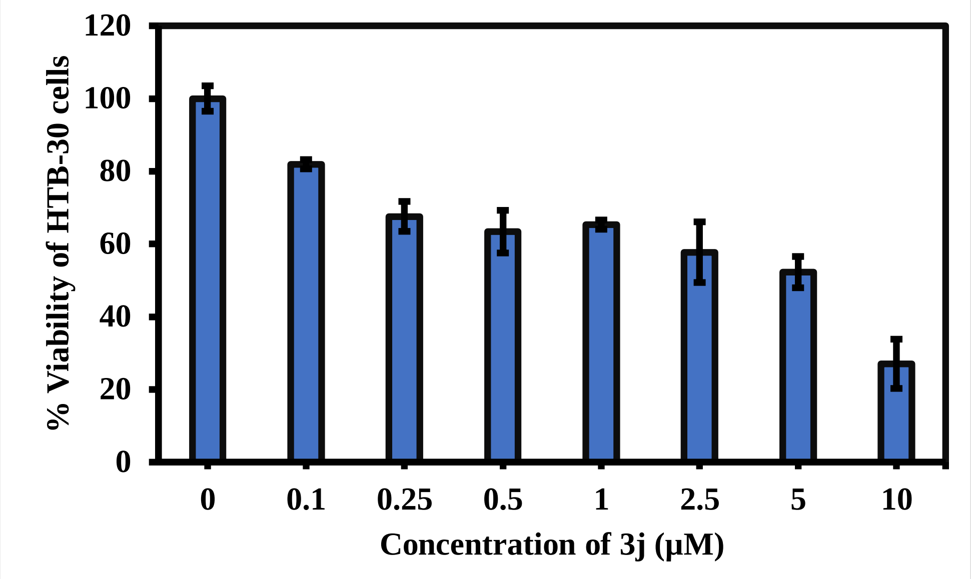

Figure S3 The IC_{50} determination of **3j**

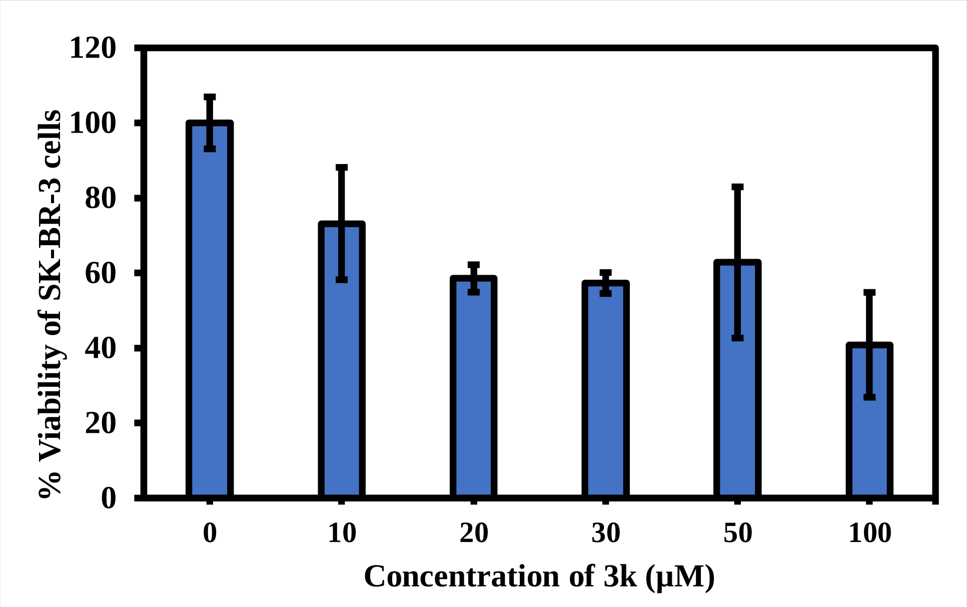

Figure S4 The IC_{50} determination of **3k**

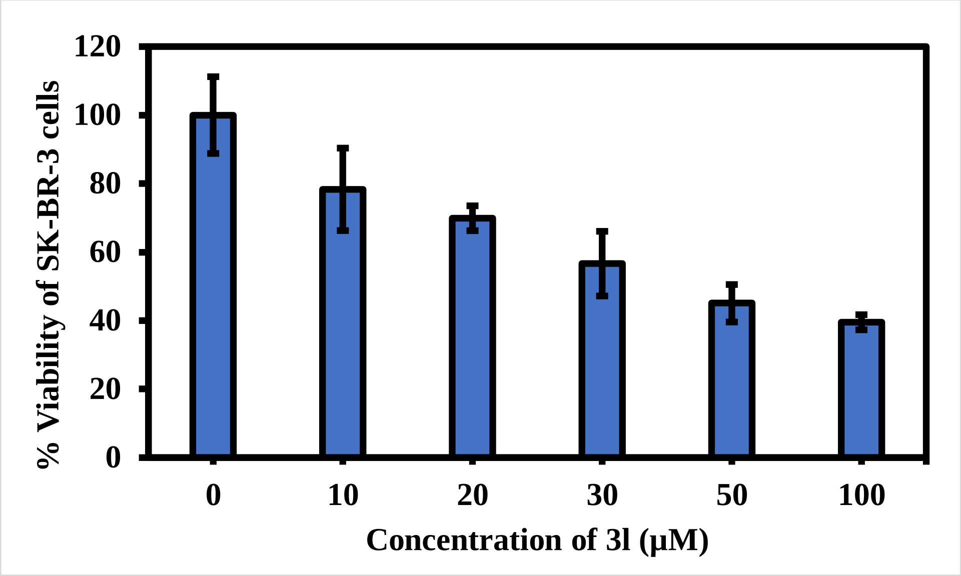

Figure S5 The IC_{50} determination of **3l**

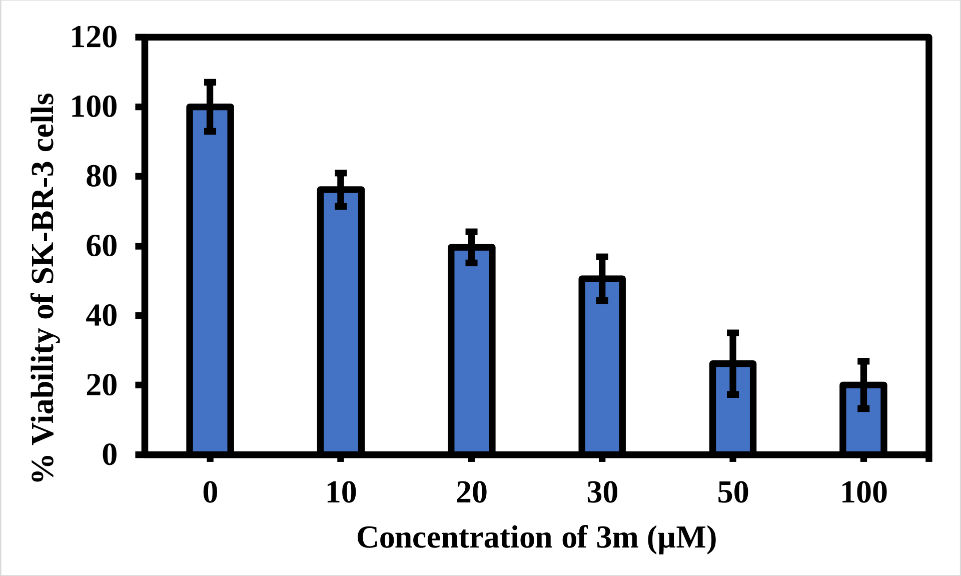

Figure S6 The IC_{50} determination of **3m**

4. Cell Viability of the untreated SK-BR-3 and MDA-MB-231 cells under dark and light irradiation

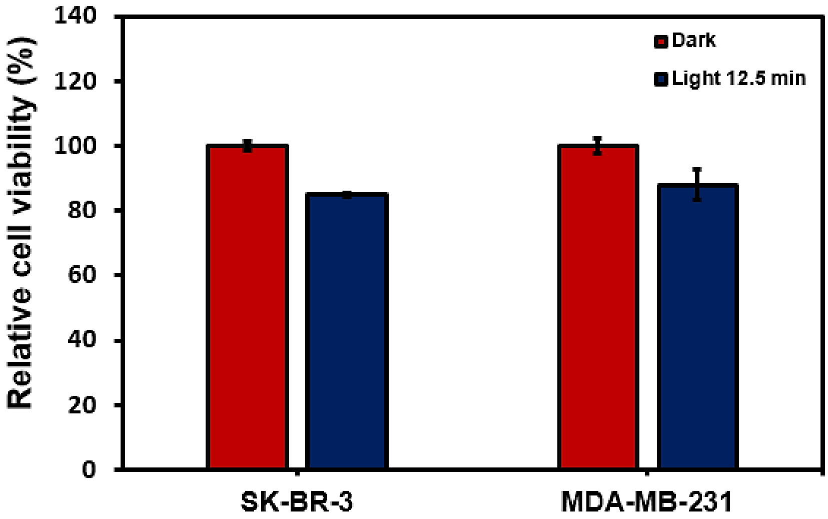

Figure S7 Cell Viability of the untreated SK-BR-3 and MDA-MB-231 cells under dark and light irradiation

5. NMR and HRMS spectra

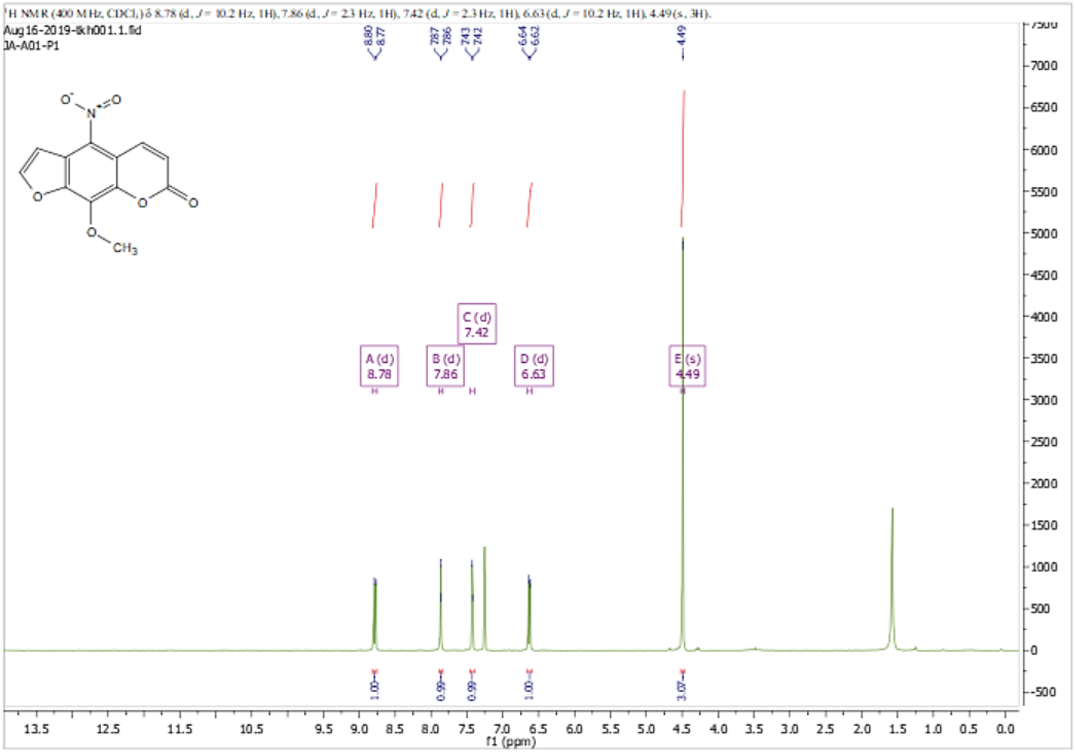

Figure S8 ^{1}H NMR spectrum of **1**

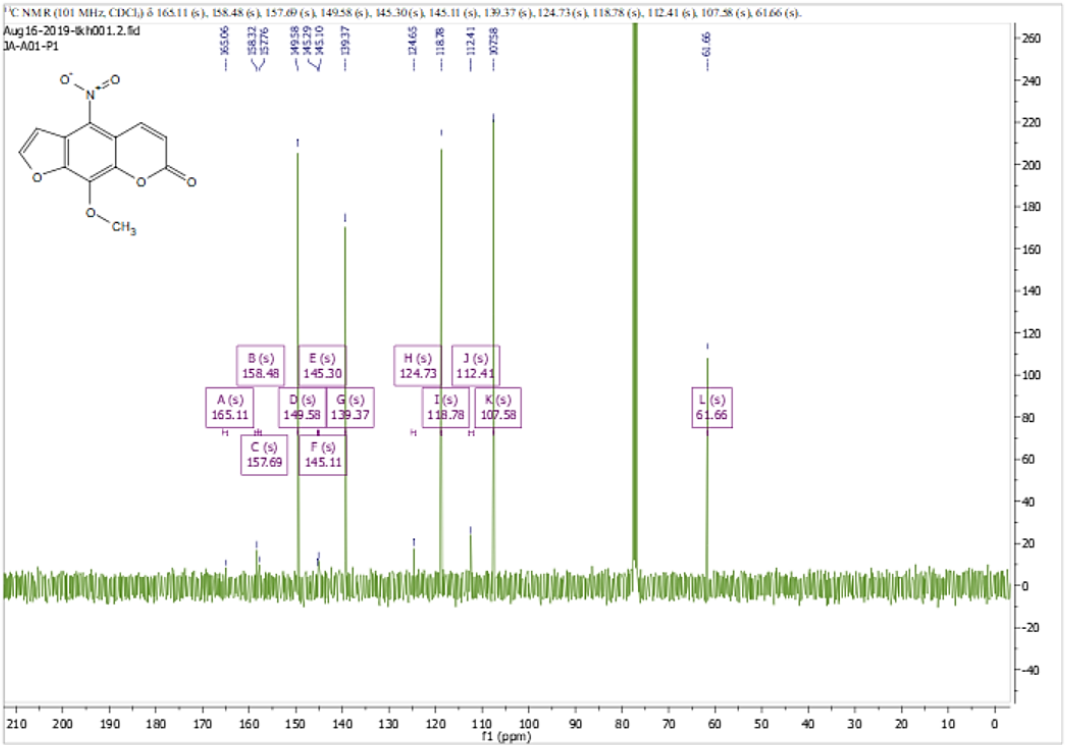

Figure S9 ^{13}C NMR spectrum of **1**

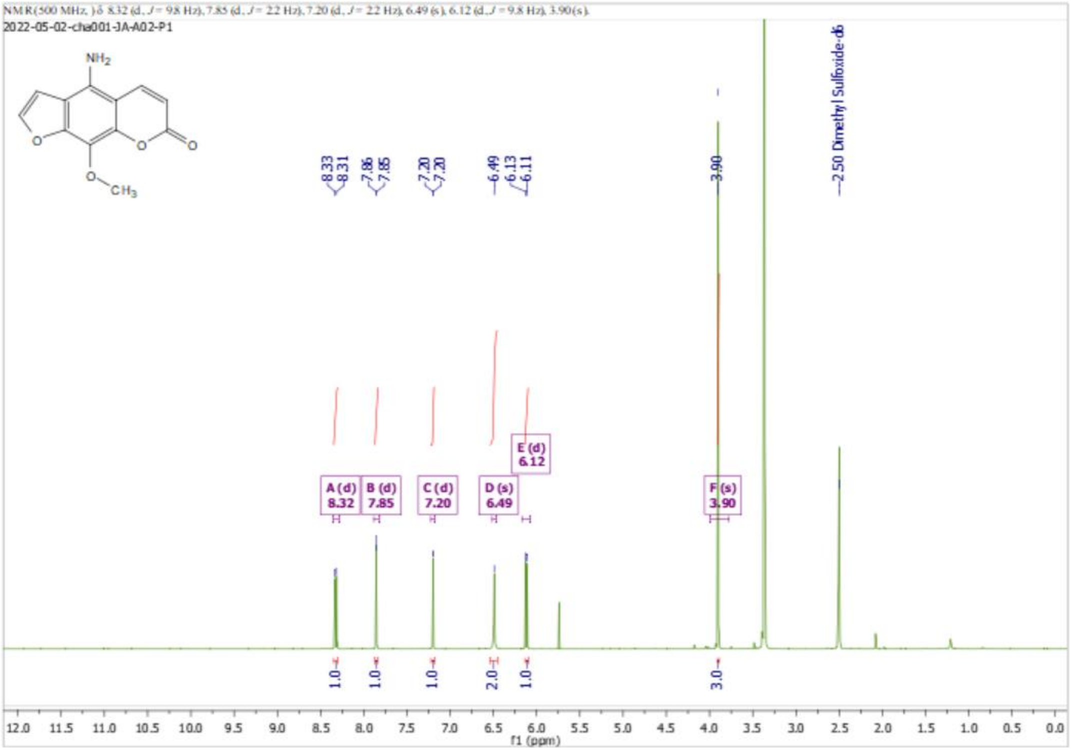

Figure S10 ¹H NMR spectrum of **2**

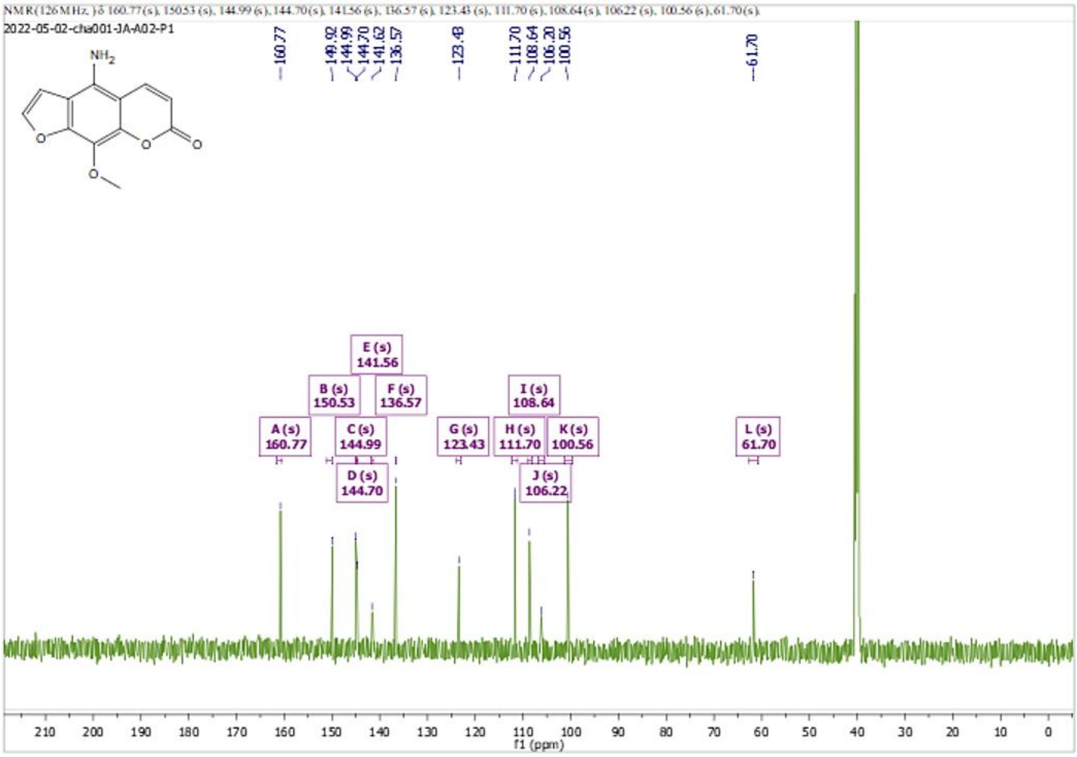

Figure S11 ¹³C NMR spectrum of **2**

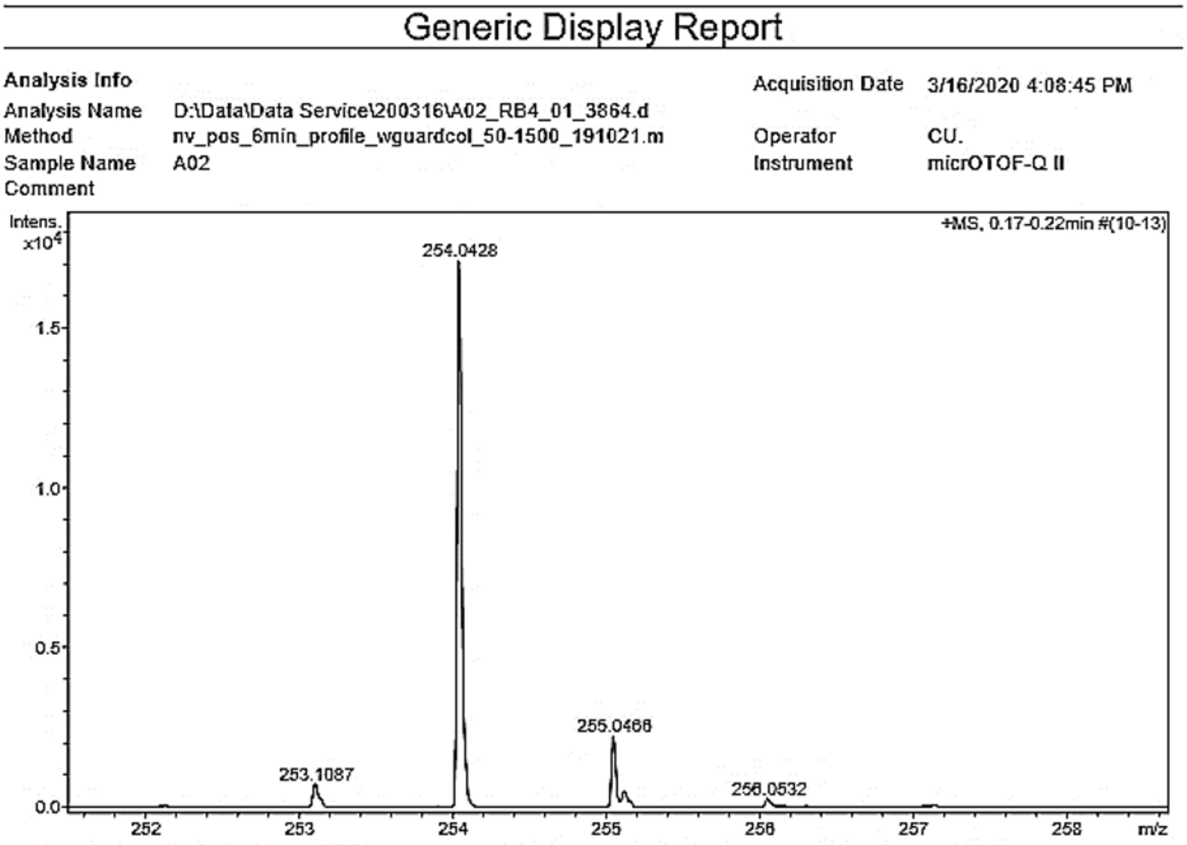

Figure S12 HRMS spectrum of **2**

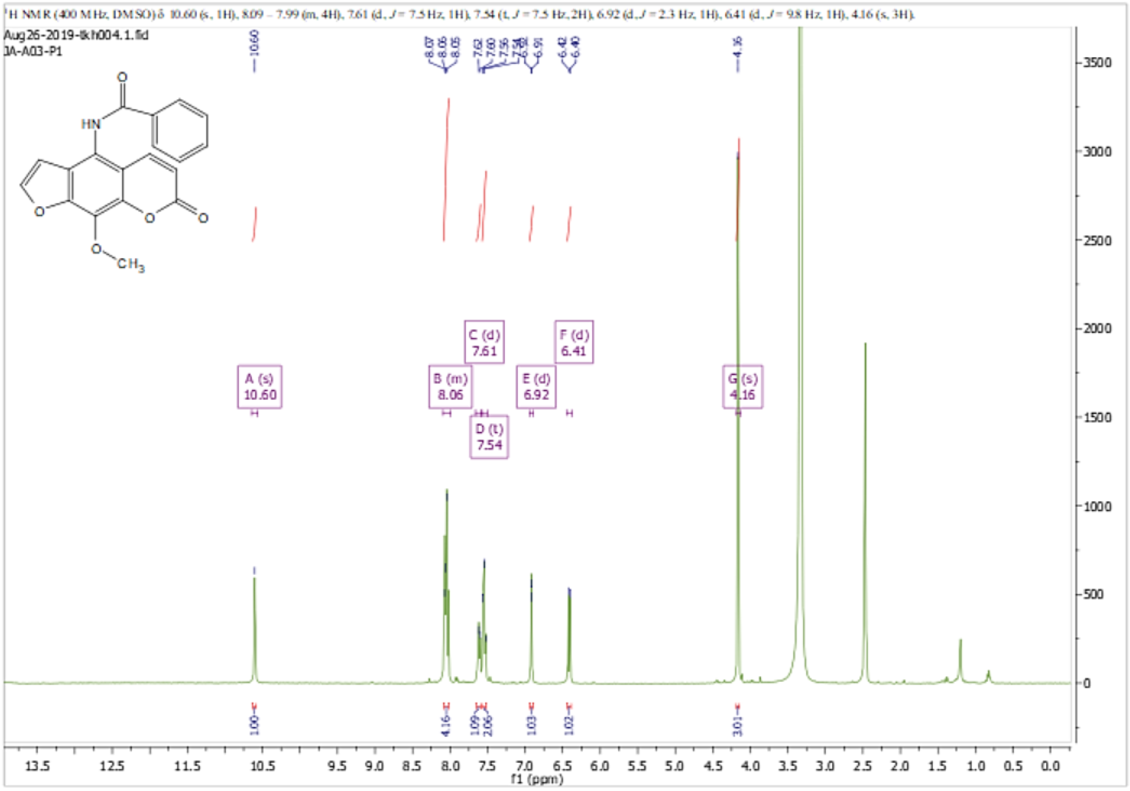

Figure S13 ^{1}H NMR spectrum of **3a**

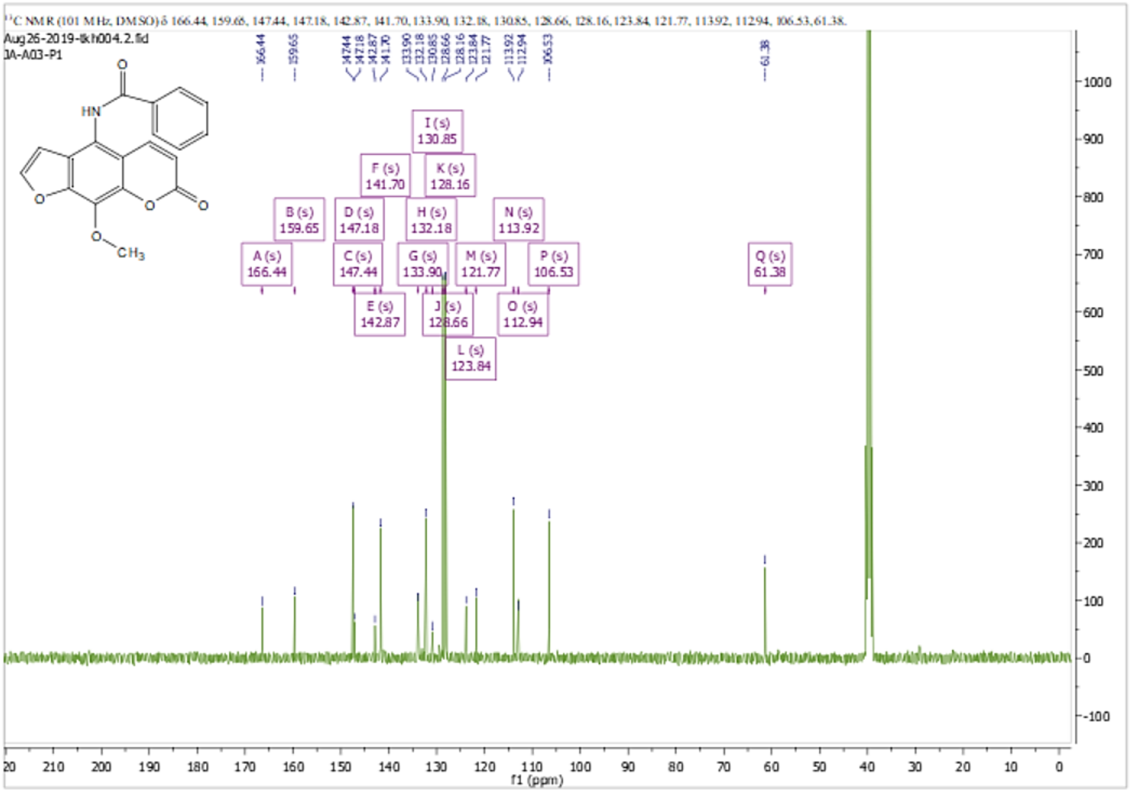

Figure S14 ^{13}C NMR spectrum of **3a**

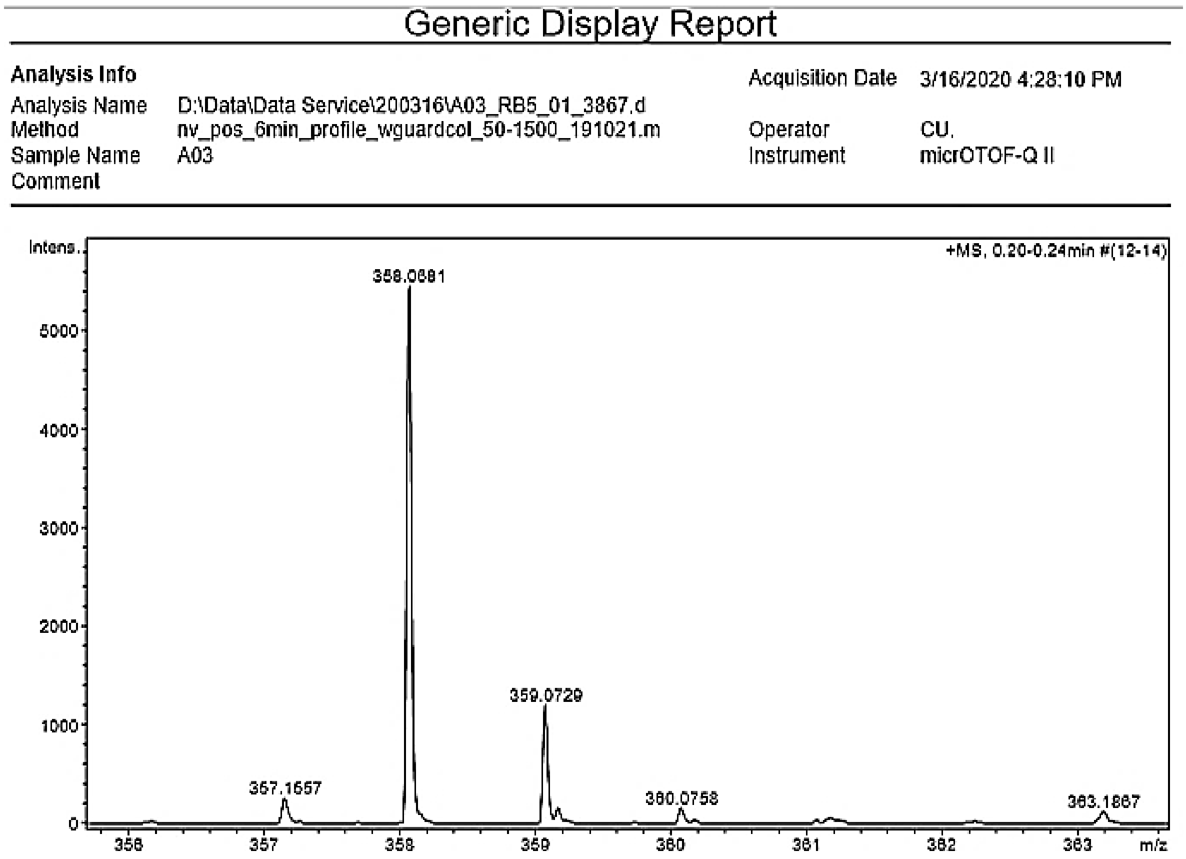

Figure S15 HRMS spectrum of **3a**

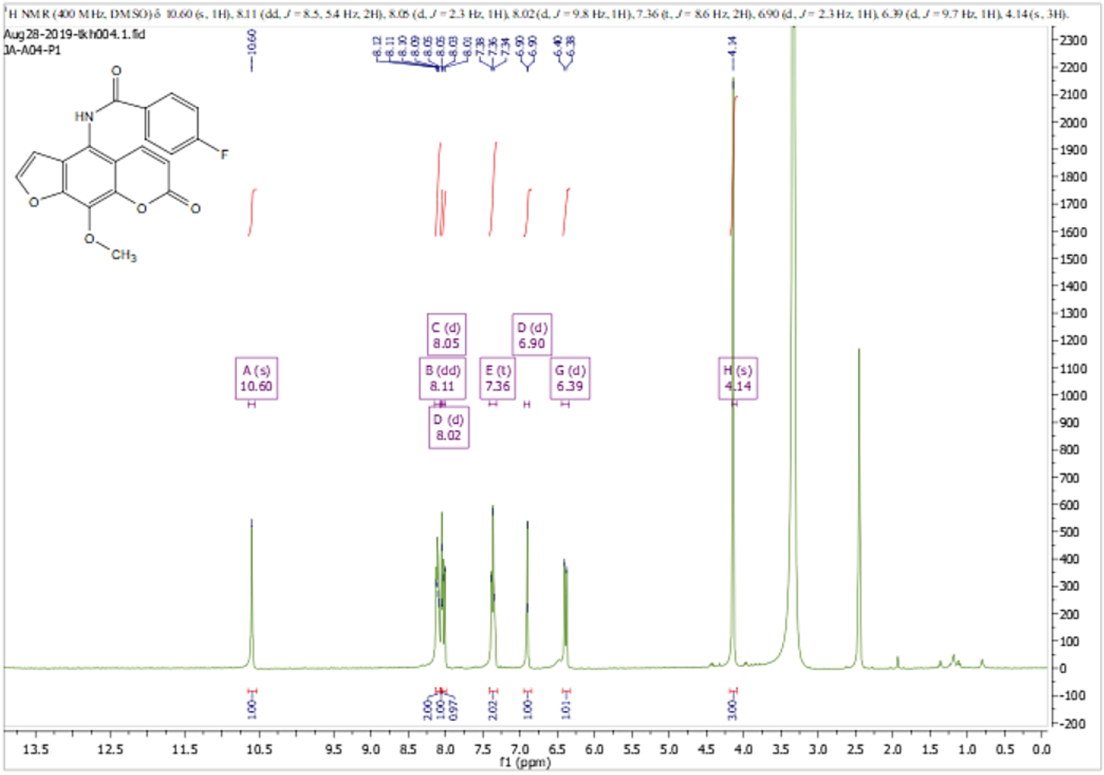

Figure S16 ^{1}H NMR spectrum of **3b**

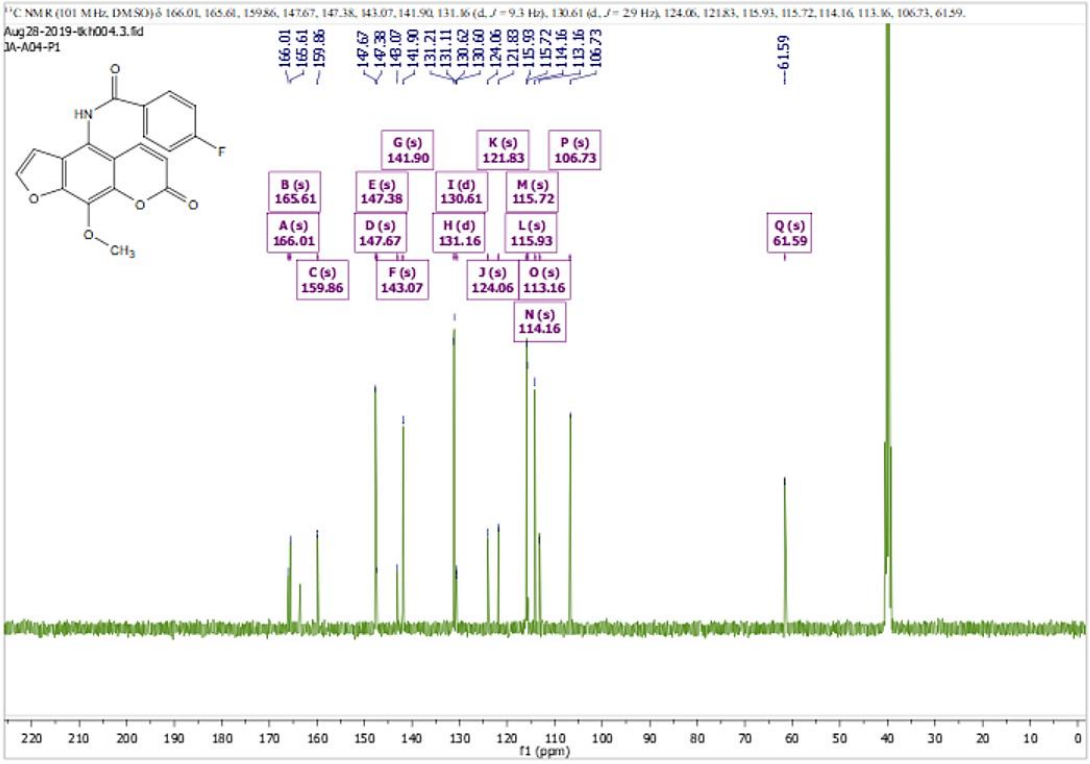

Figure S17 ^{13}C NMR spectrum of **3b**

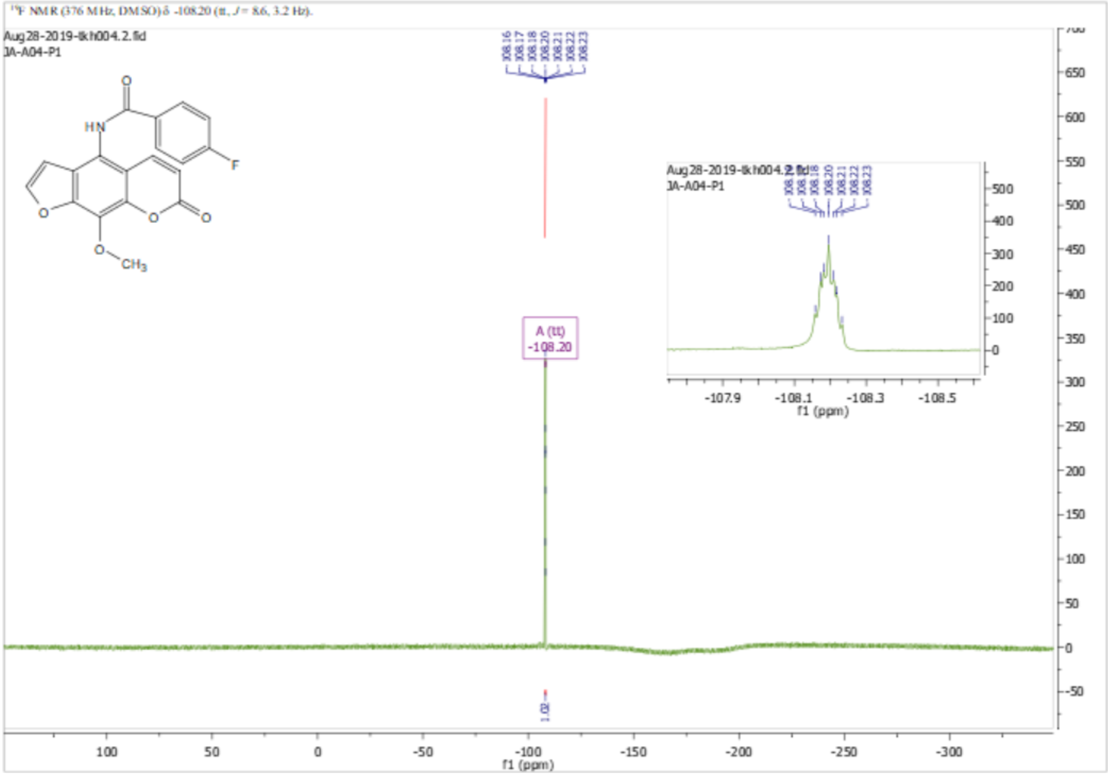

Figure S18 ^{19}F NMR spectrum of **3b**

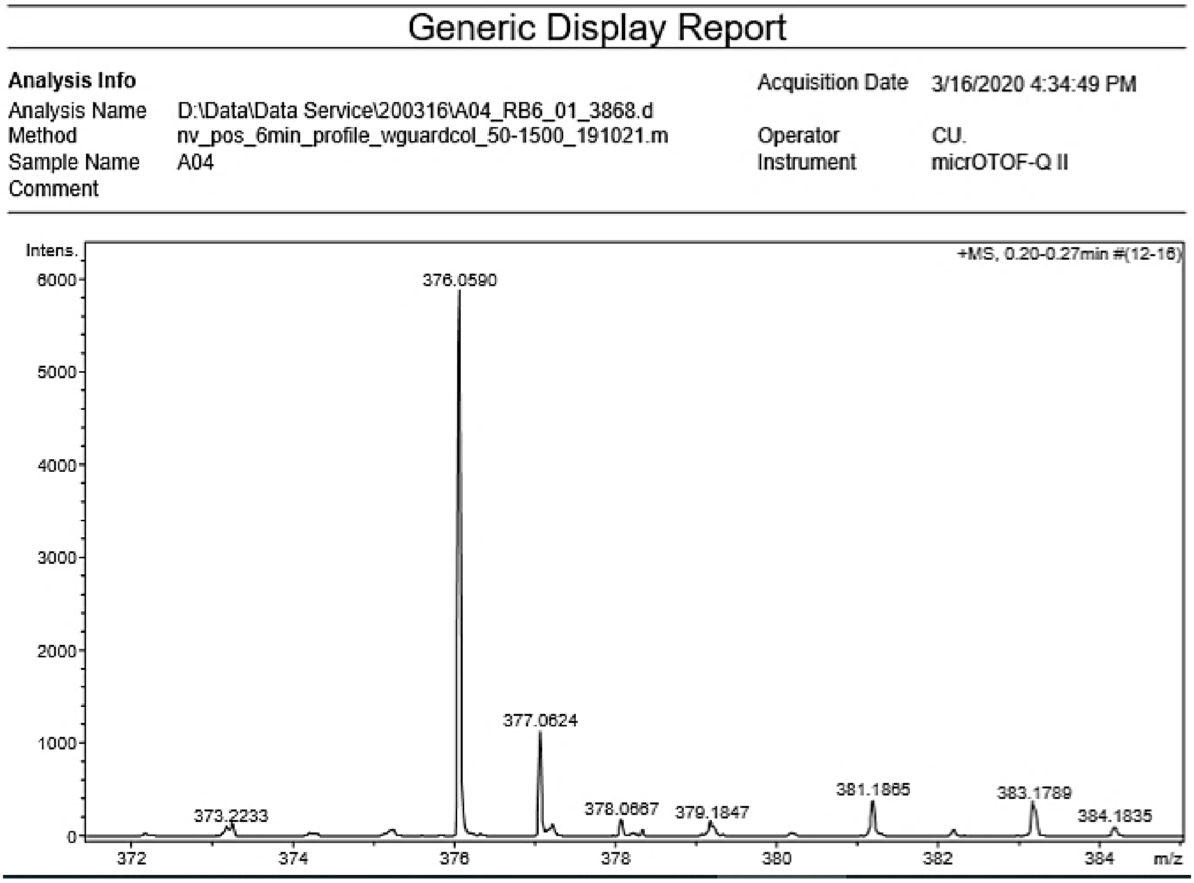

Figure S19 HRMS spectrum of **3b**

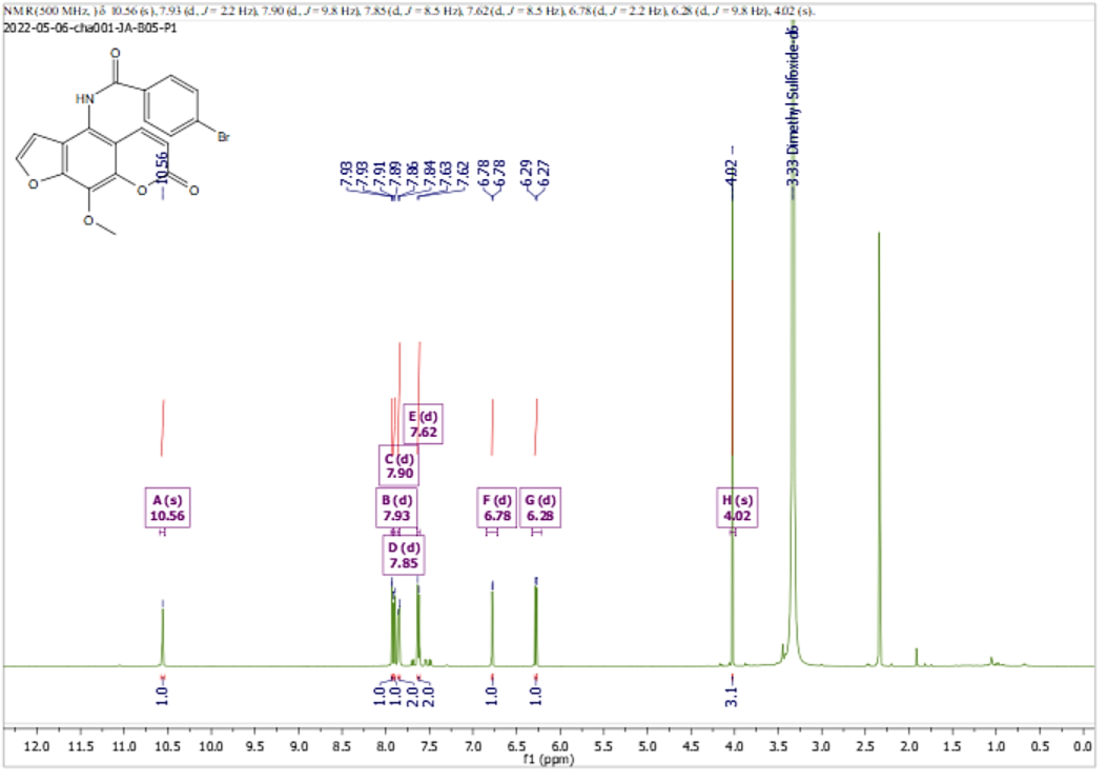

Figure S20 ^{1}H NMR spectrum of **3c**

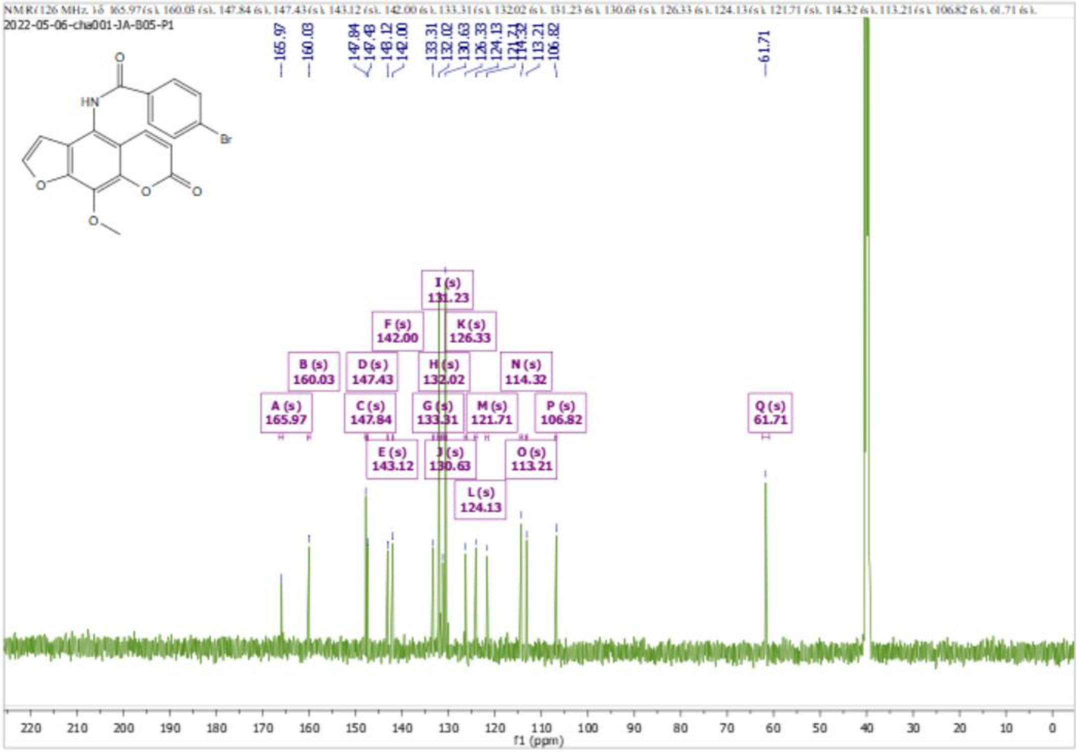

Figure S21 ^{13}C NMR spectrum of **3c**

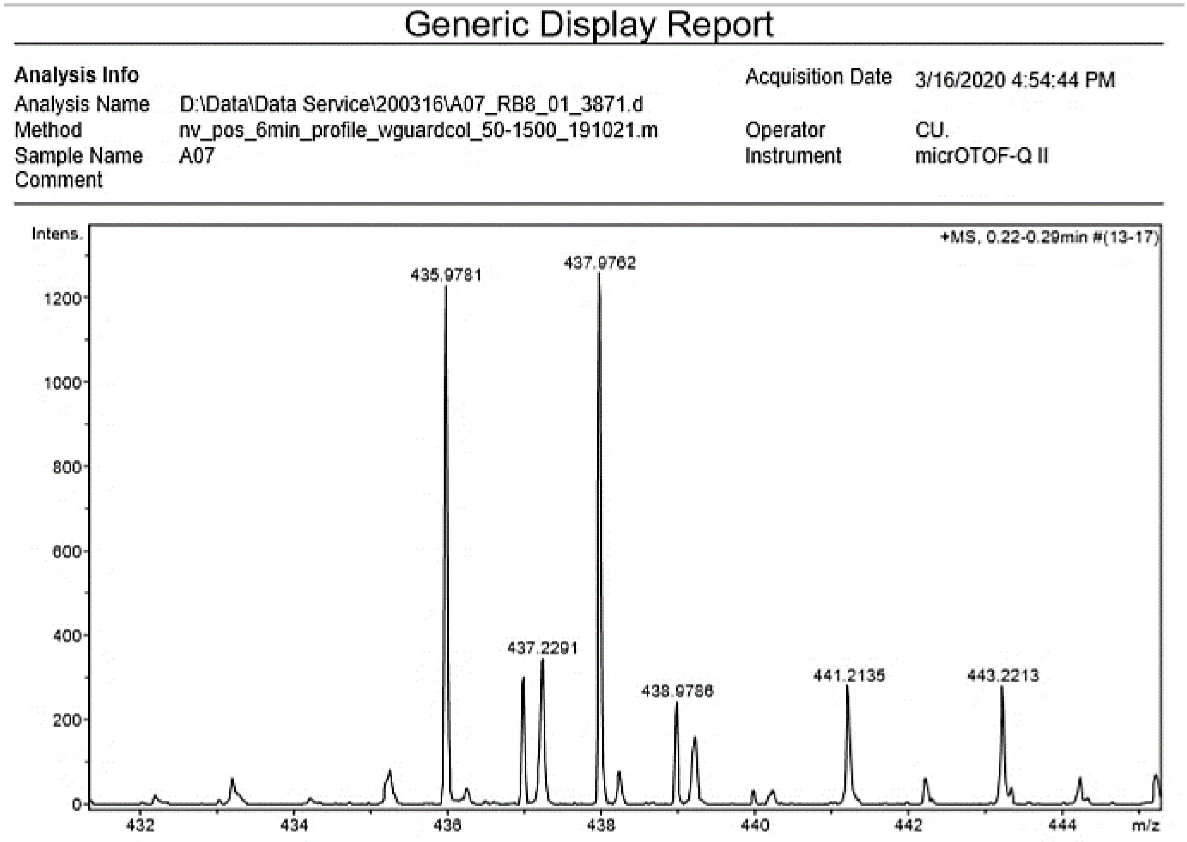

Figure S22 HRMS spectrum of **3c**

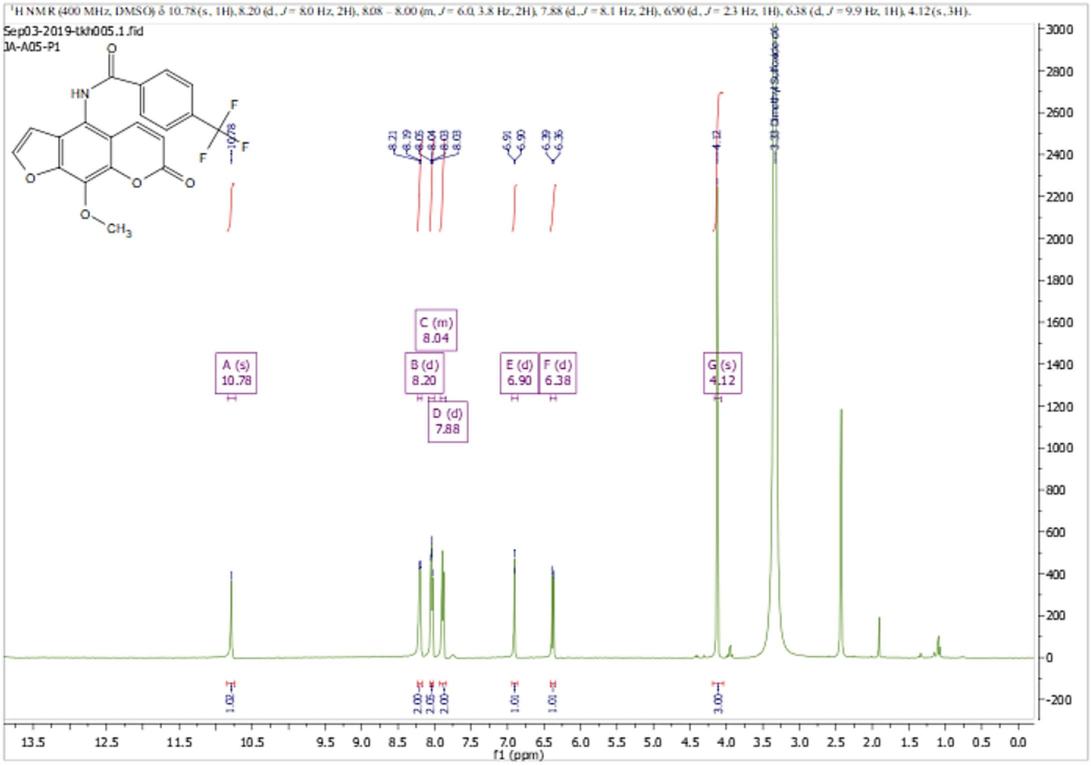

Figure S23 ^{1}H NMR spectrum of **3d**

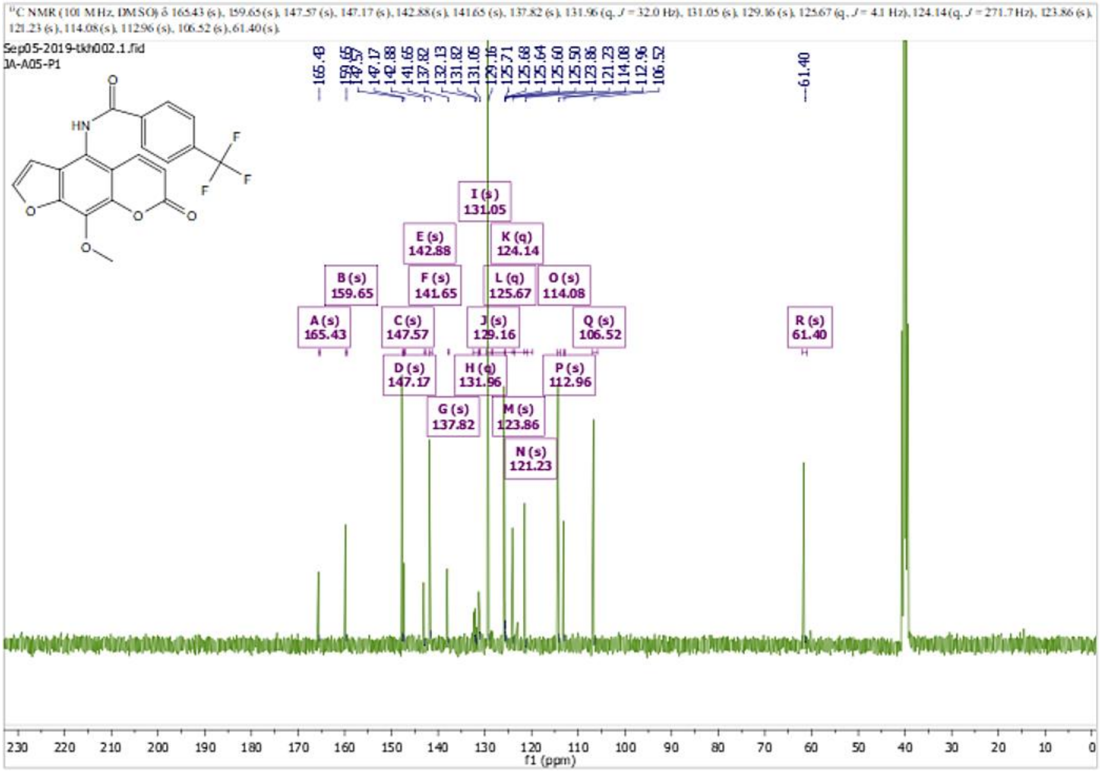

Figure S24 ^{13}C NMR spectrum of **3d**

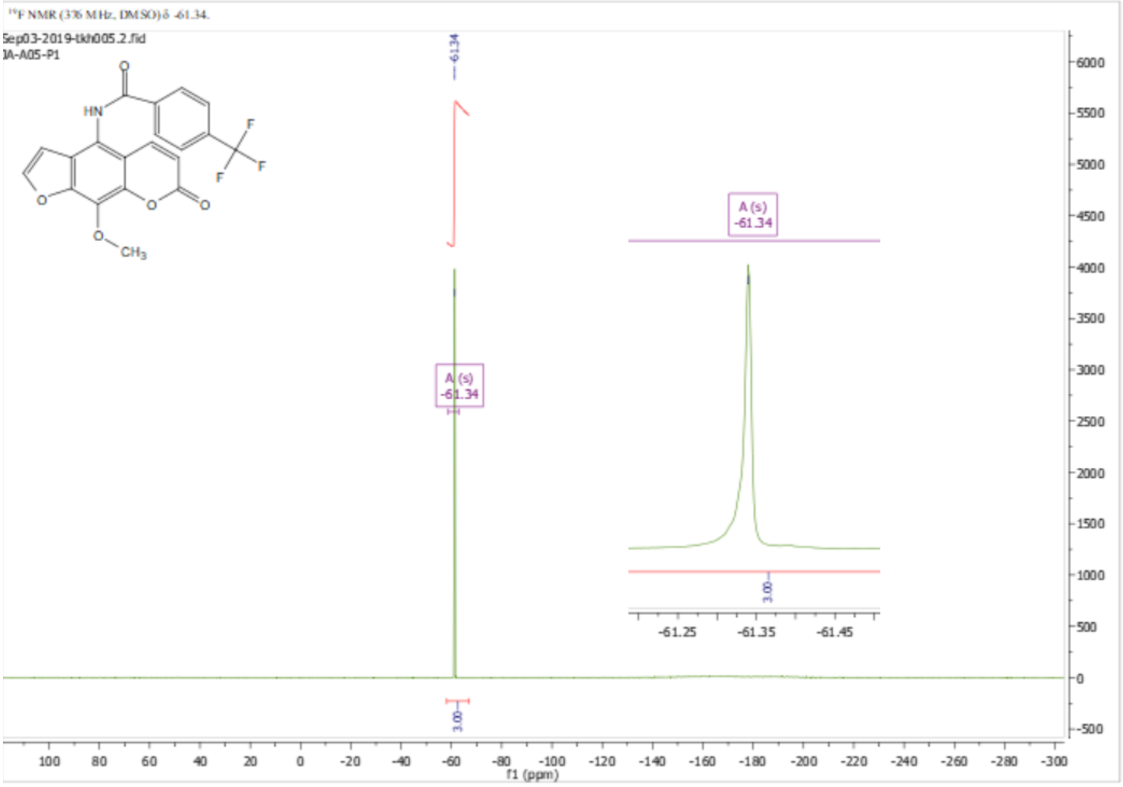

Figure S25 ^{19}F NMR spectrum of **3d**

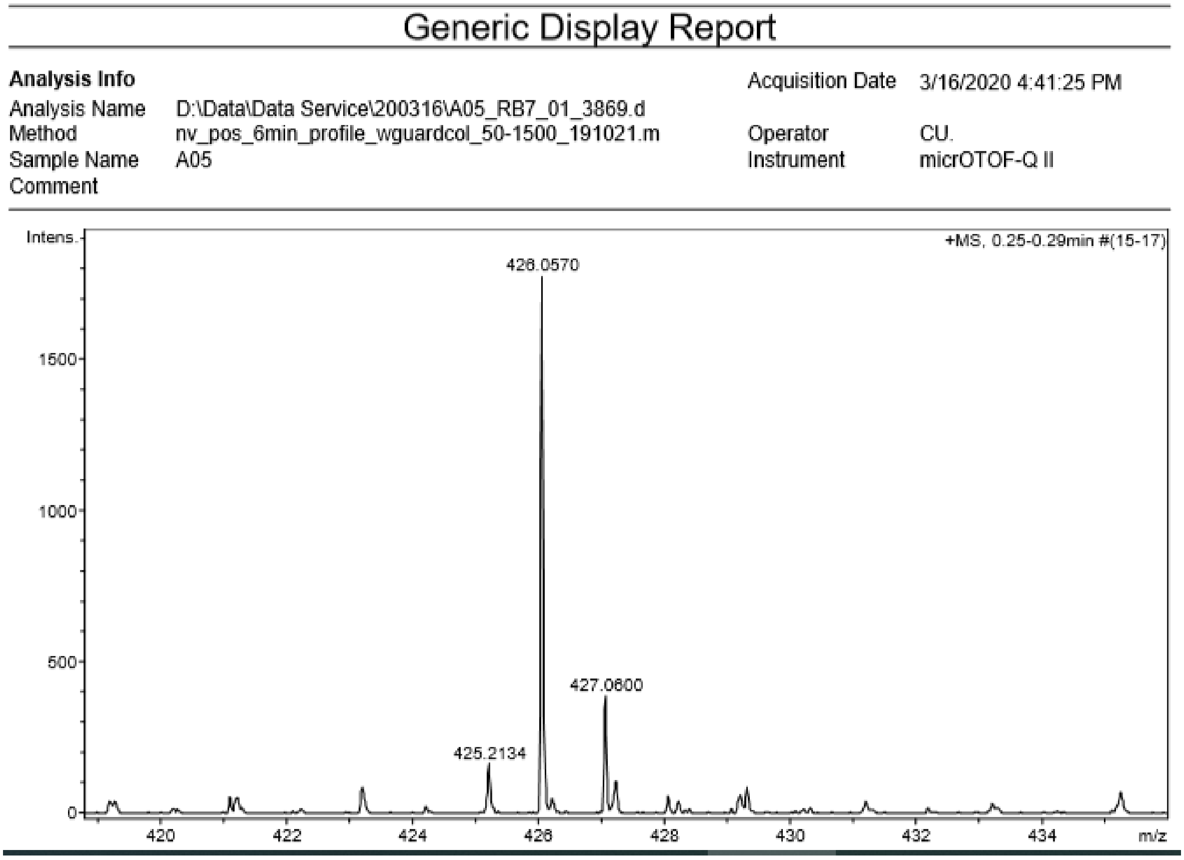

Figure S26 HRMS spectrum of **3d**

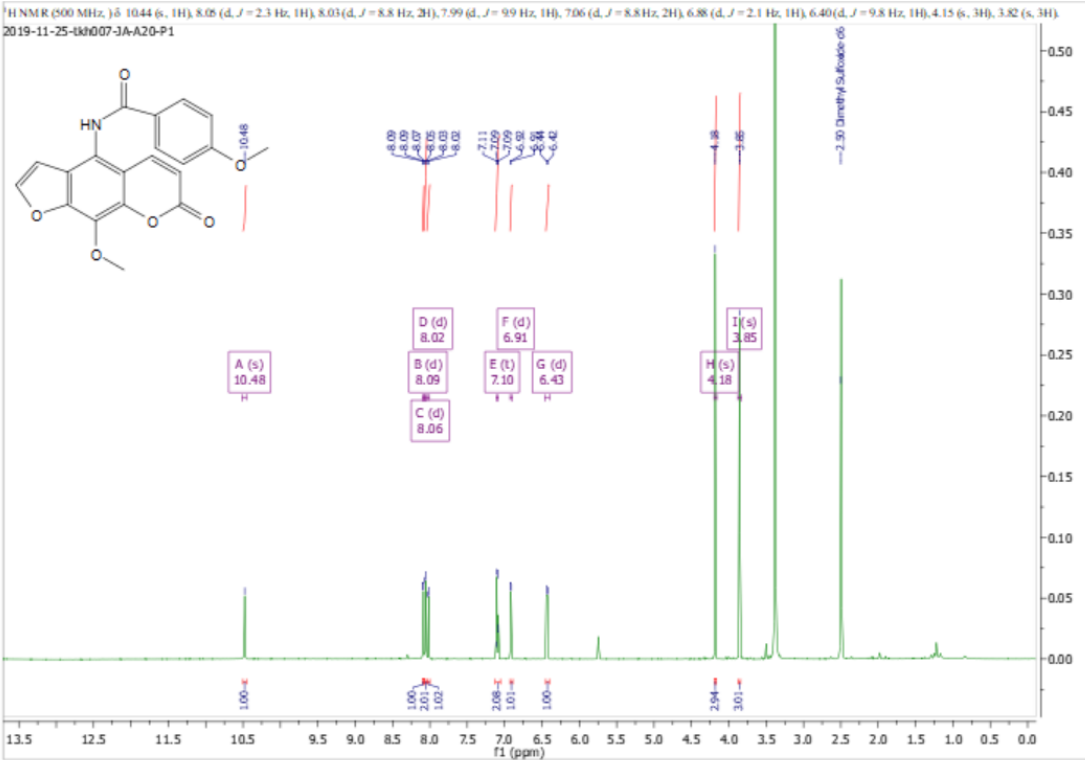

Figure S27 ^{1}H NMR spectrum of **3e**

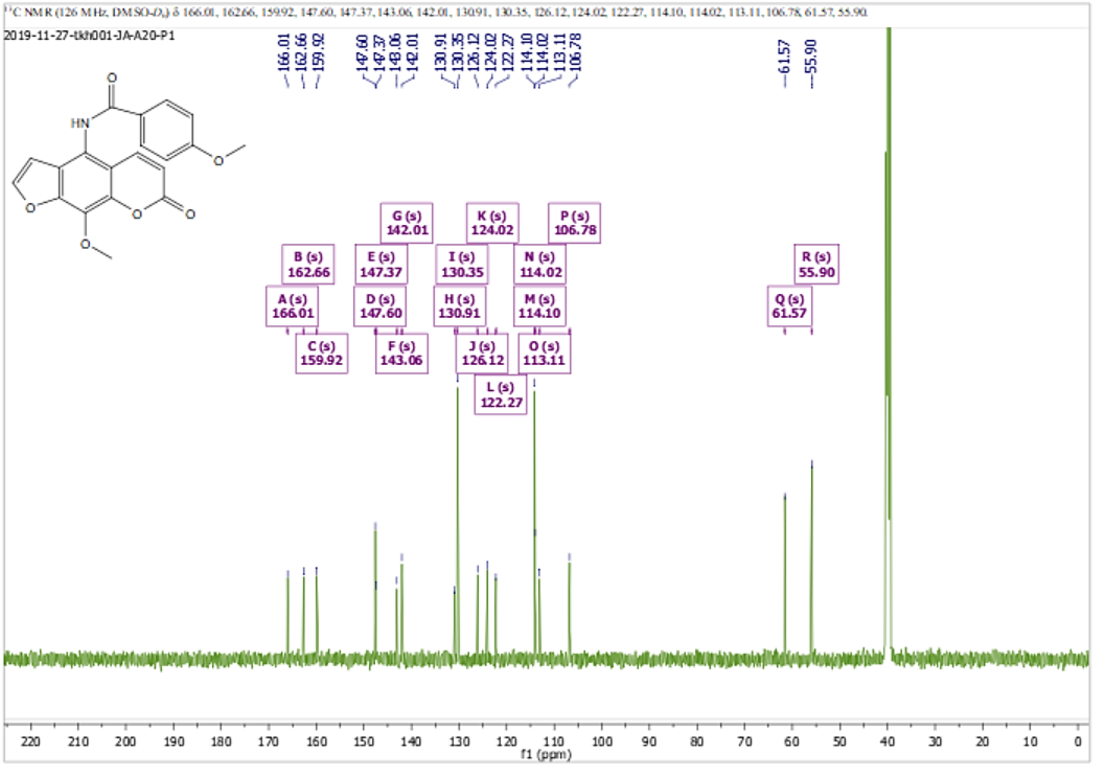

Figure S28 ^{13}C NMR spectrum of **3e**

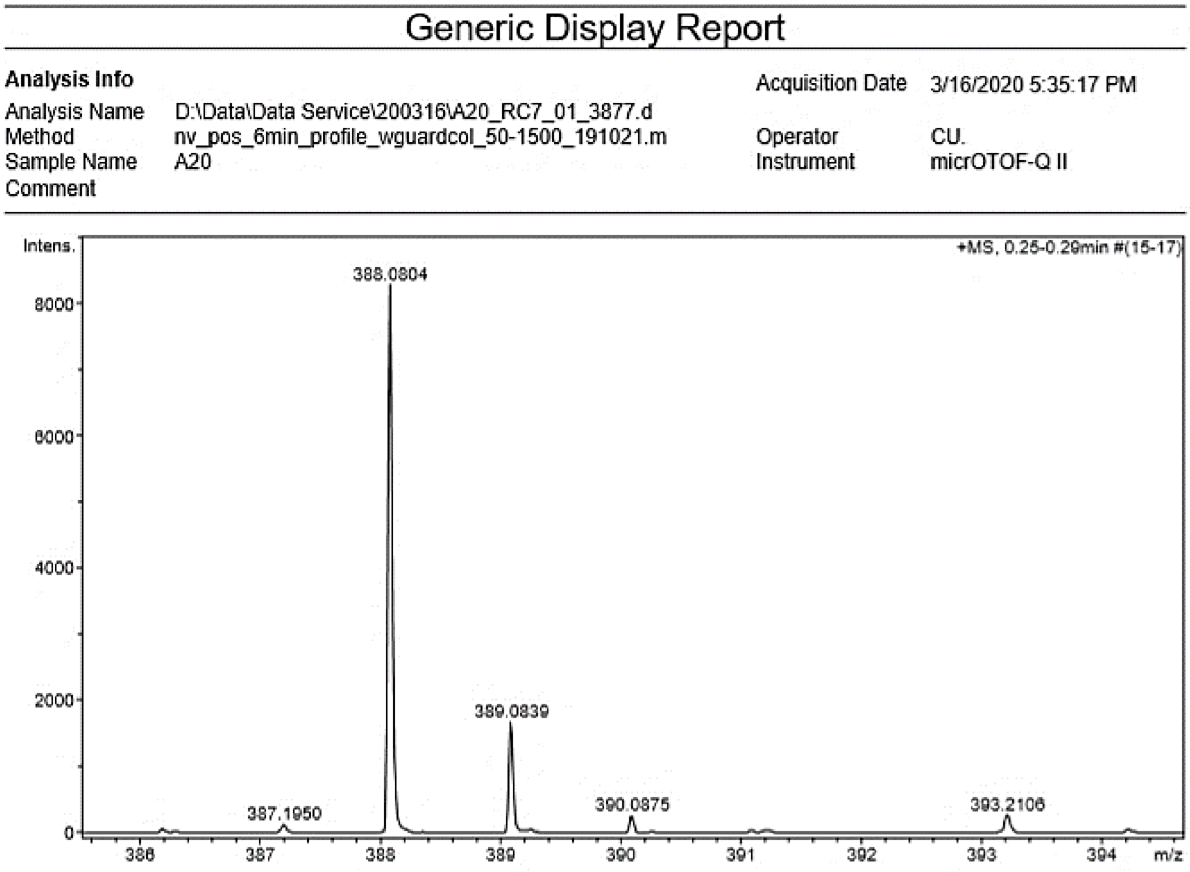

Figure S29 HRMS spectrum of **3e**

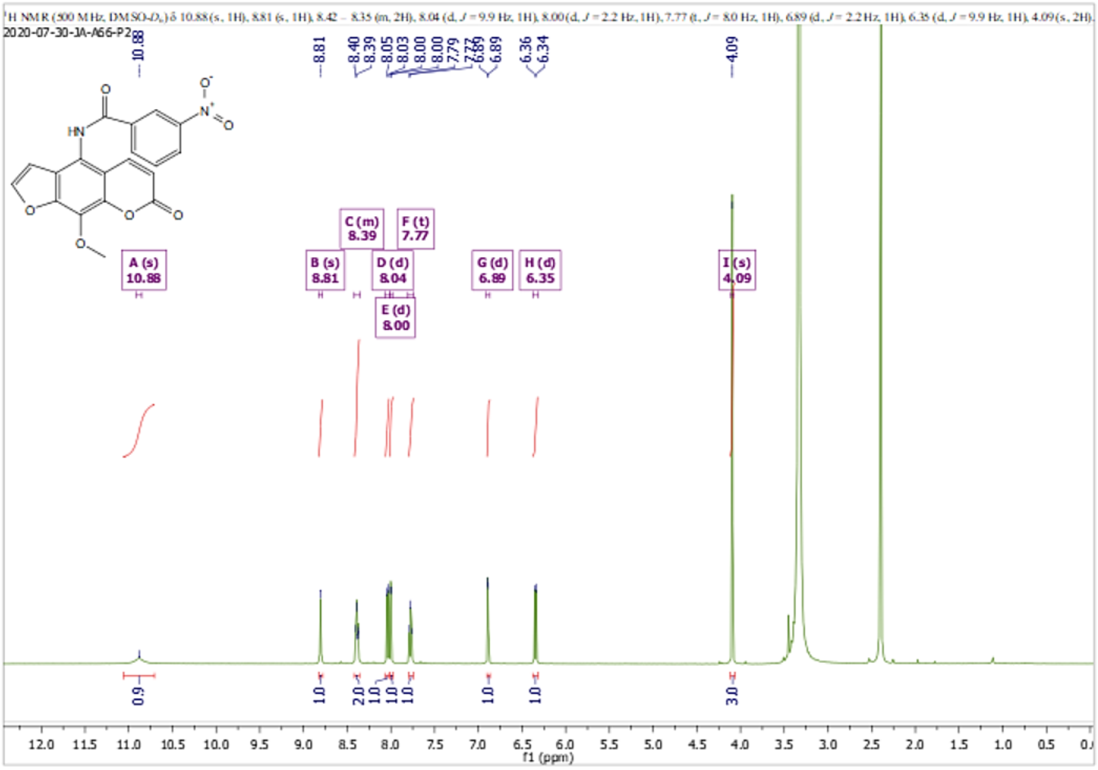

Figure S30 ^{1}H NMR spectrum of **3f**

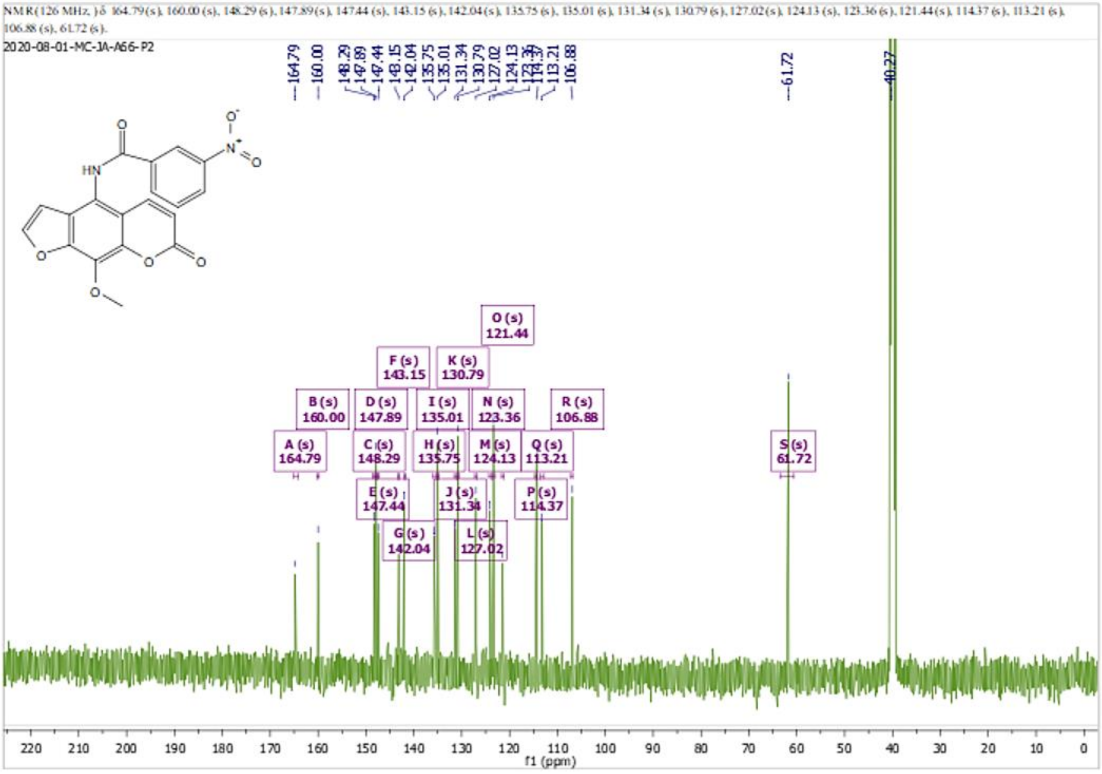

Figure S31 ^{13}C NMR spectrum of **3f**

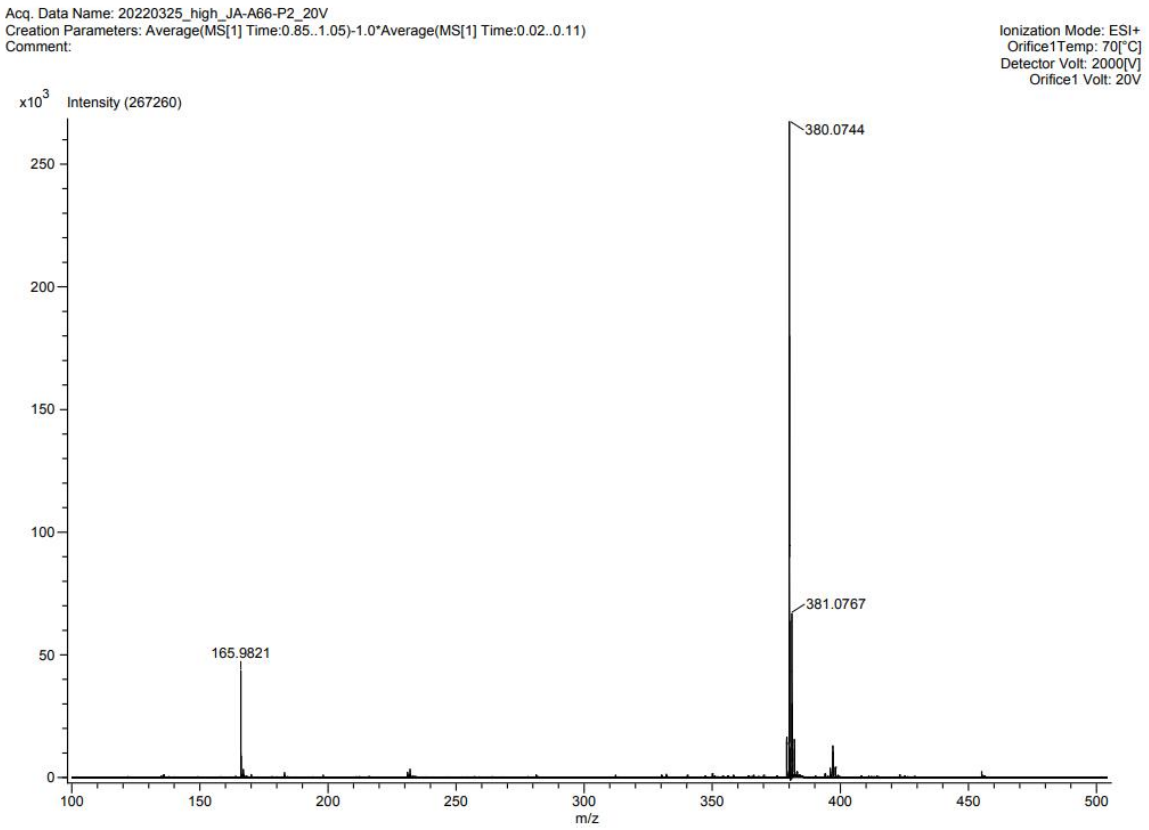

Figure S32 HRMS spectrum of **3f**

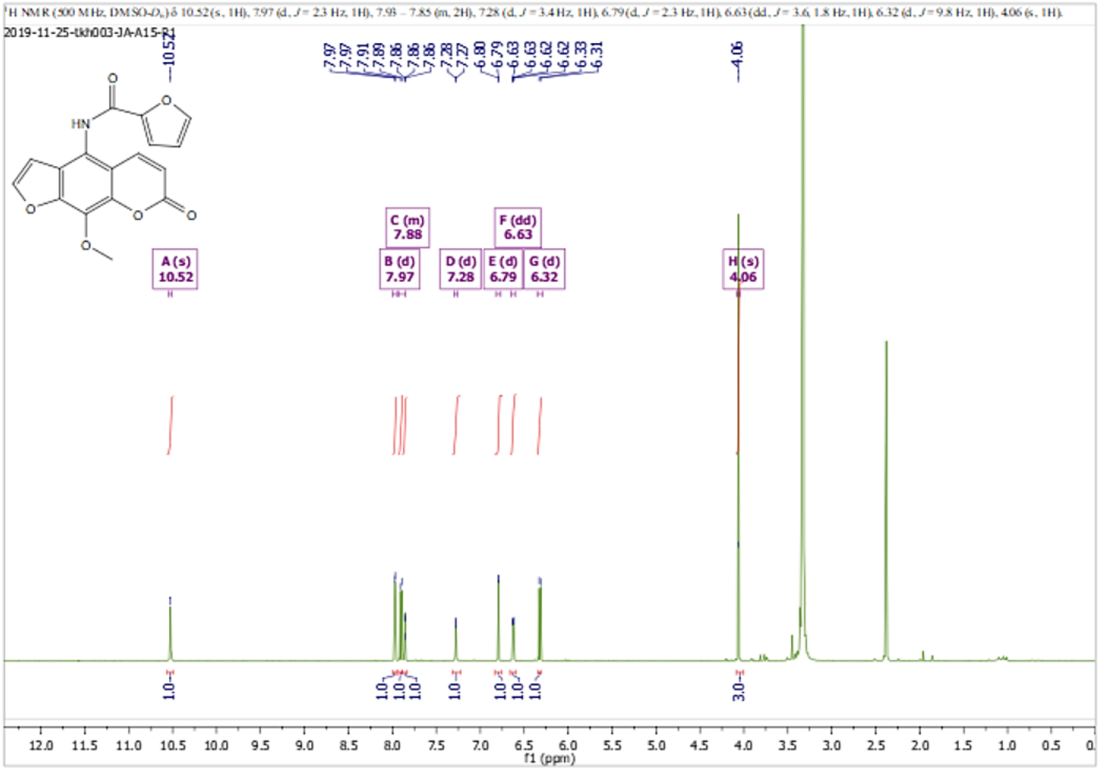

Figure S33 ^{1}H NMR spectrum of **3g**

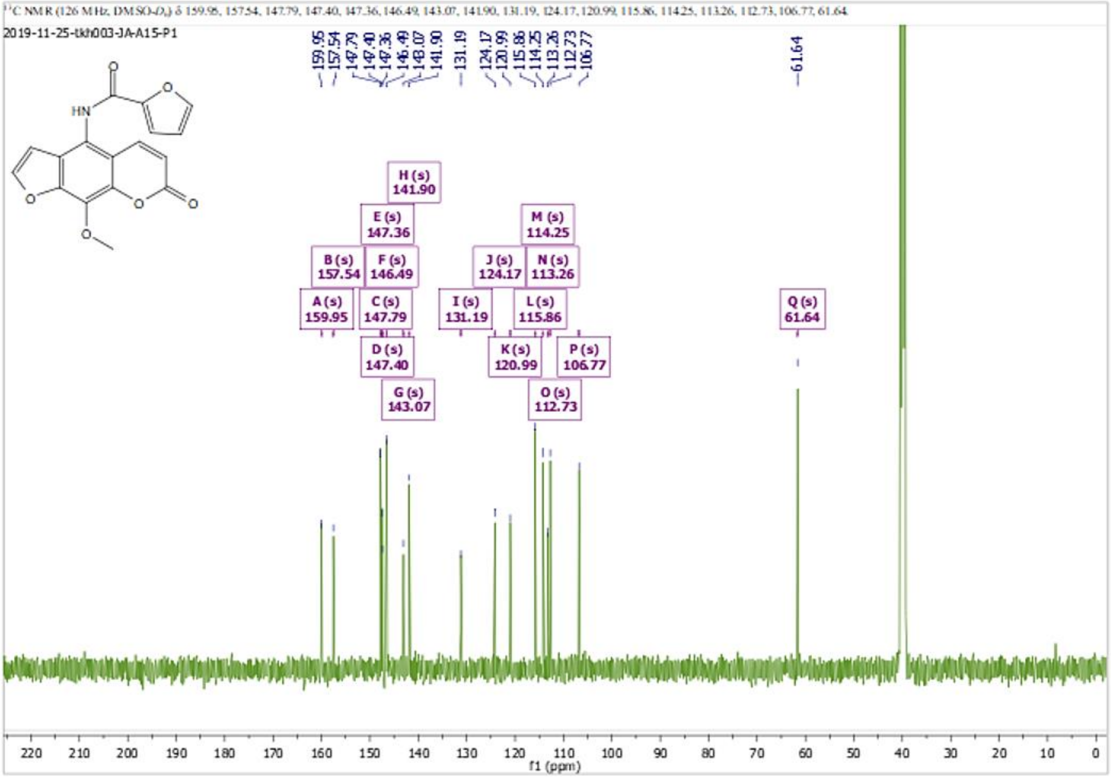

Figure S34 ^{13}C NMR spectrum of **3g**

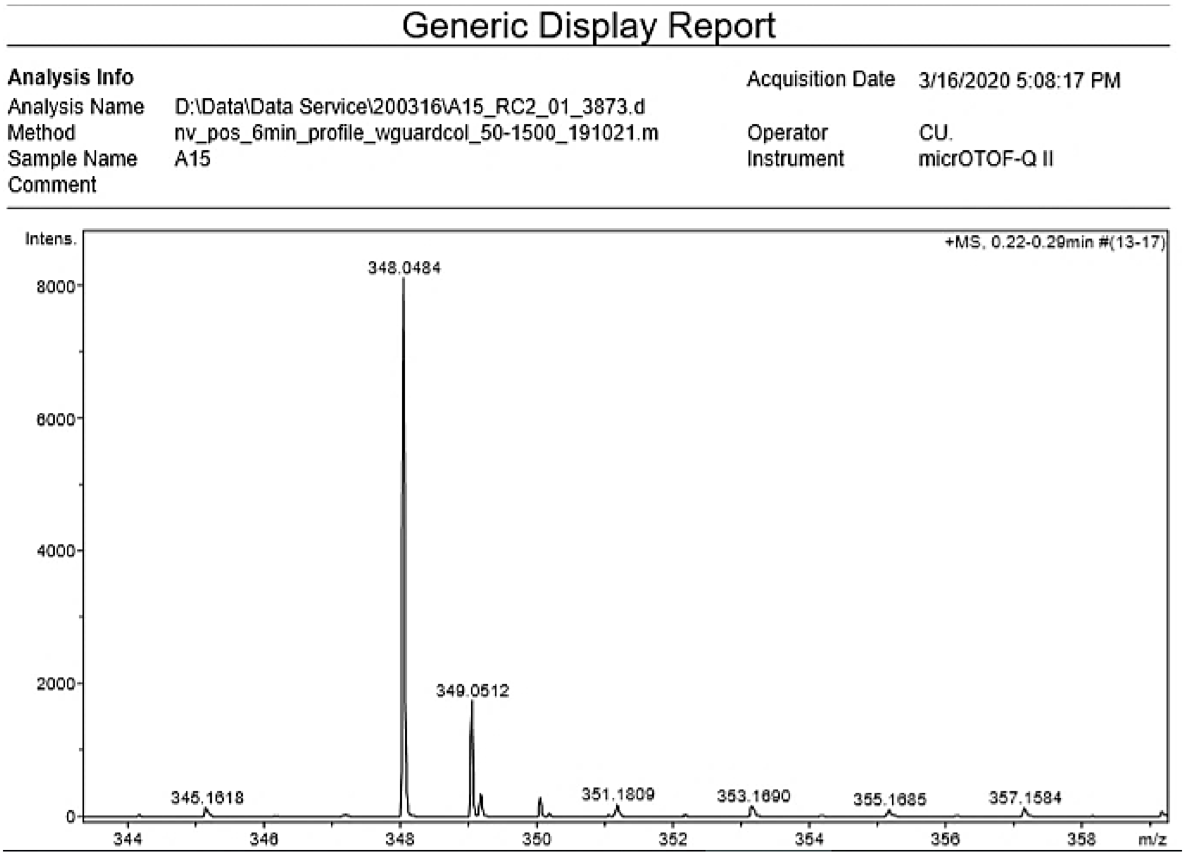

Figure S35 HRMS spectrum of **3g**

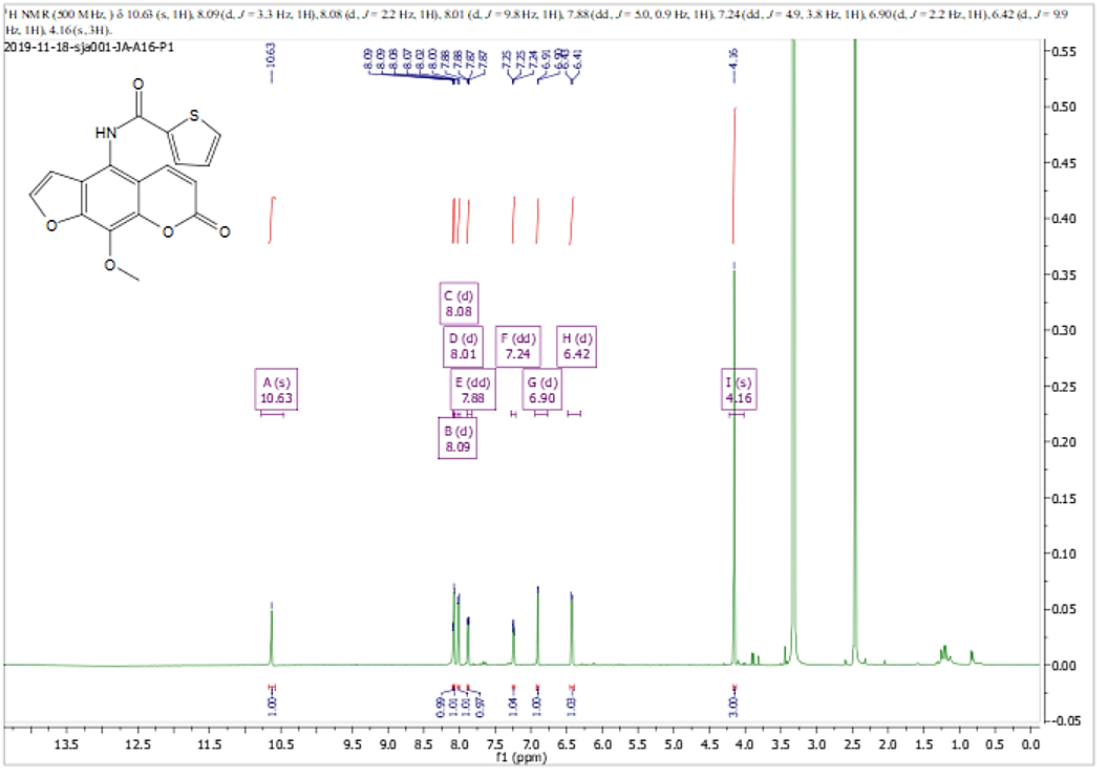

Figure S36 ^{1}H NMR spectrum of **3h**

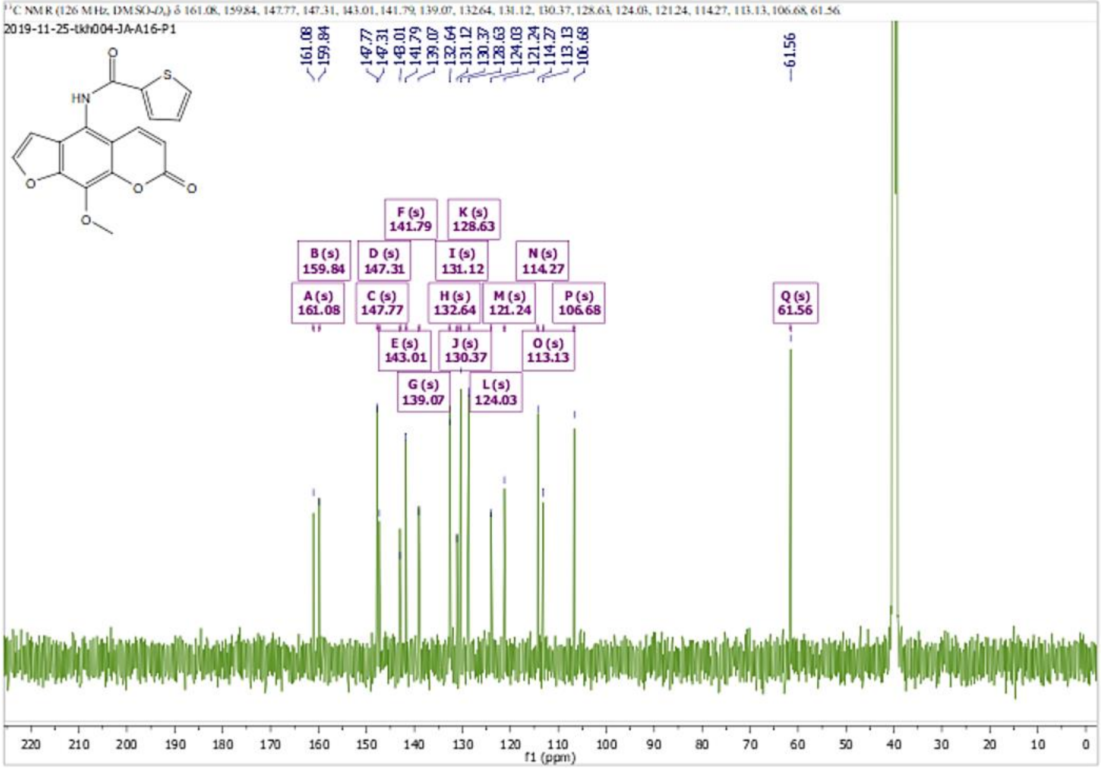

Figure S37 ^{13}C NMR spectrum of **3h**

Generic Display Report

**Analysis Info**		Acquisition Date	3/16/2020 5:15:02 PM
Analysis Name	D:\Data\Data Service\200316\A16_RC3_01_3874.d		
Method	nv_pos_6min_profile_wguardcol_50-1500_191021.m	Operator	CU.
Sample Name	A16	Instrument	micrOTOF-Q II
Comment			

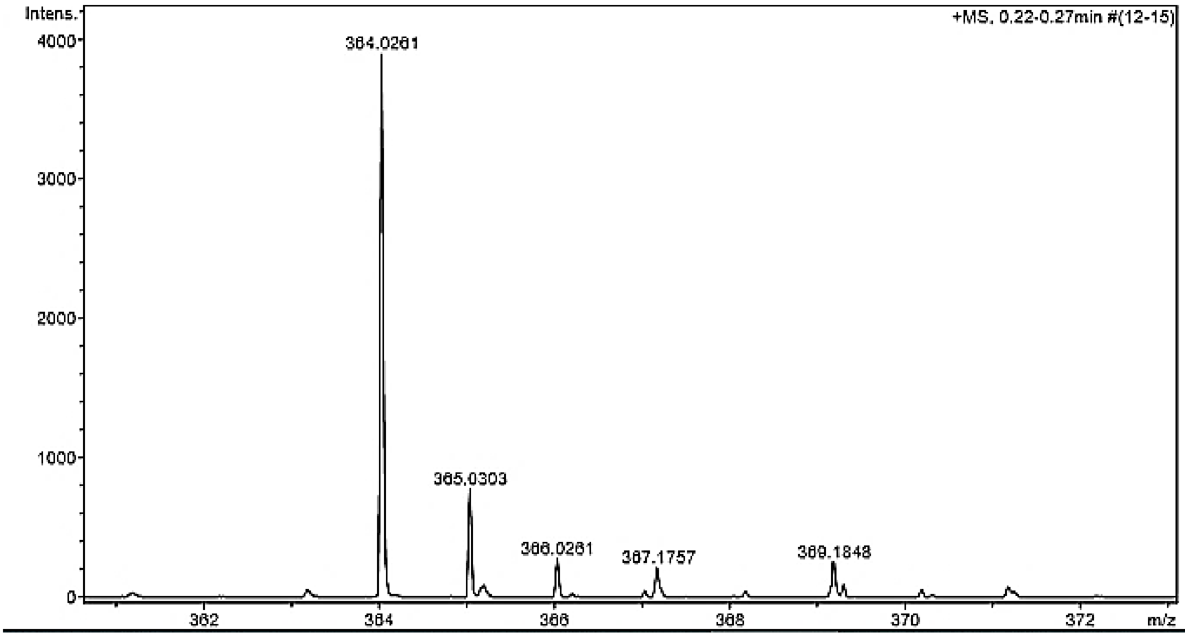

Figure S38 HRMS spectrum of **3h**

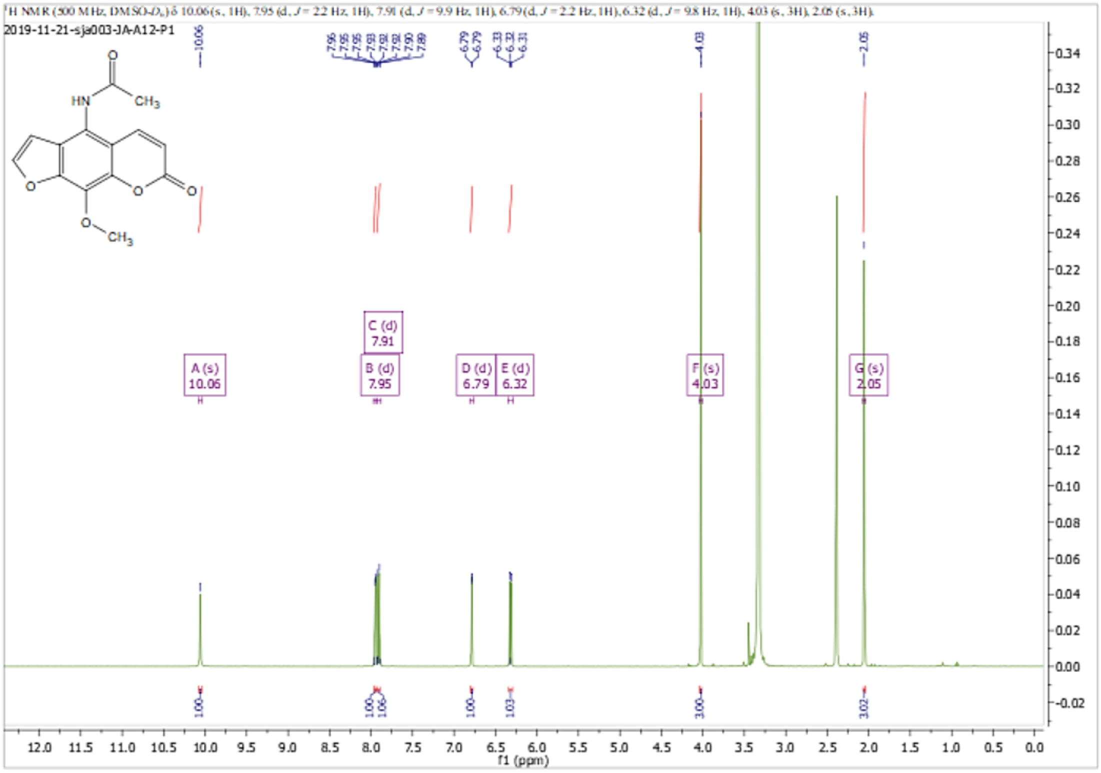

Figure S39 ^{1}H NMR spectrum of **3i**

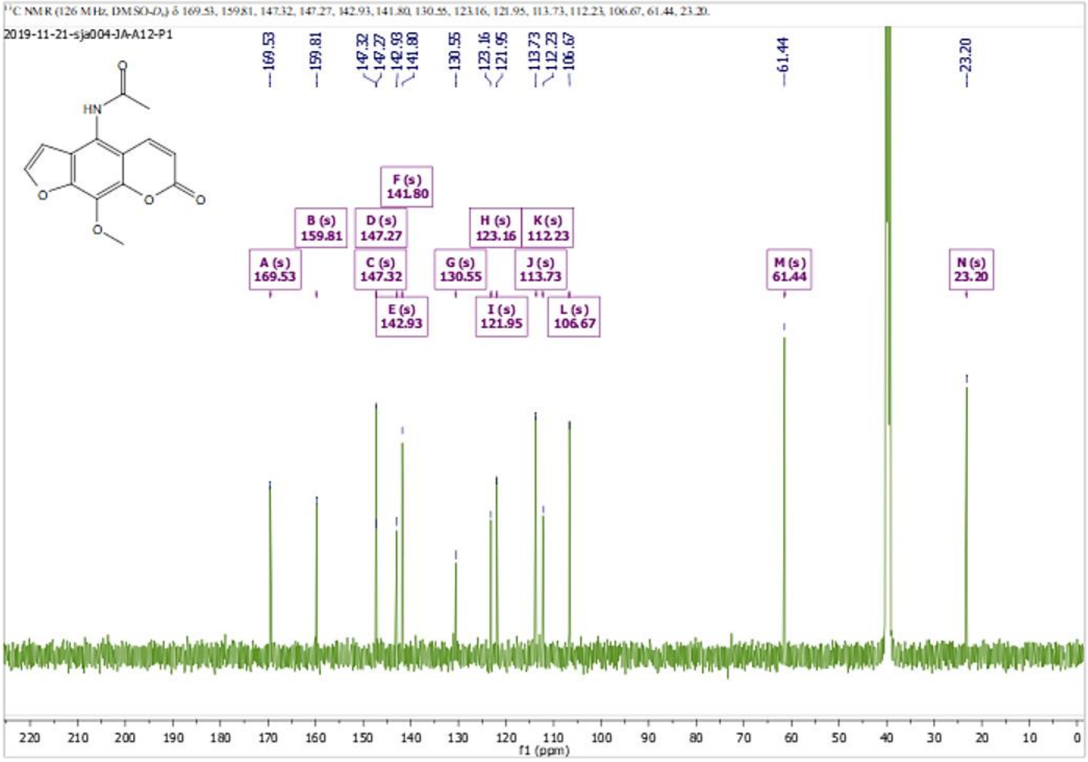

Figure S40 ^{13}C NMR spectrum of **3i**

Generic Display Report

Analysis Info			
Analysis Name	D:\Data\Data Service\200316\A12_RC1_01_3872.d	Acquisition Date	3/16/2020 5:01:31 PM
Method	nv_pos_6min_profile_wguardcol_50-1500_191021.m	Operator	CU.
Sample Name	A12	Instrument	micrOTOF-Q II
Comment			

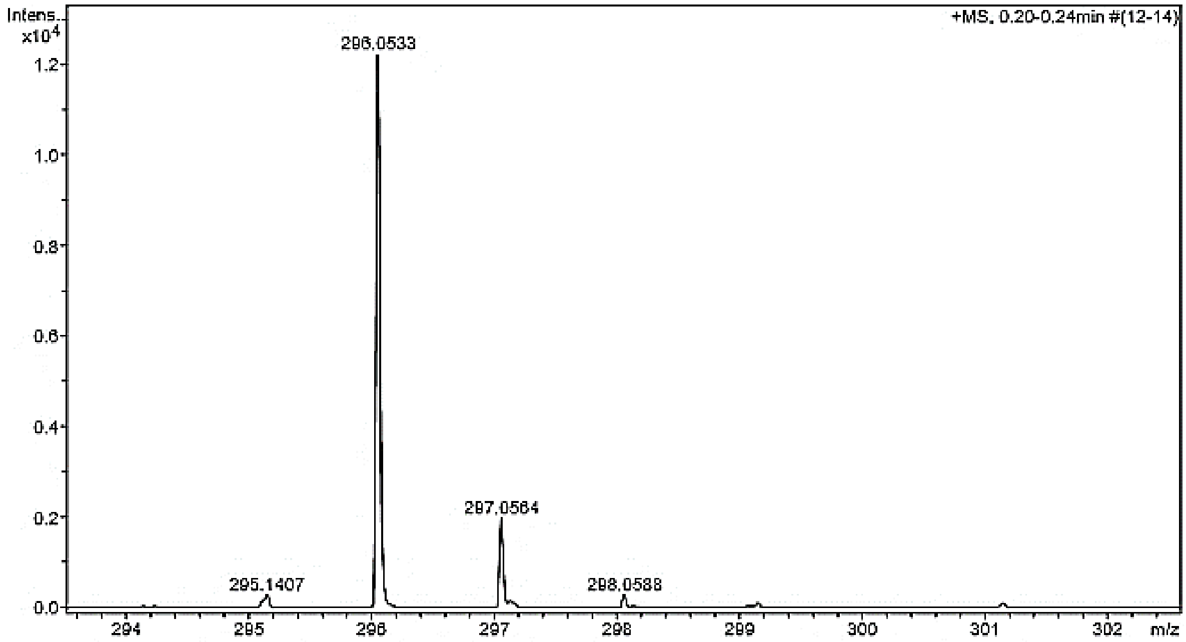

Figure S41 HRMS spectrum of **3i**

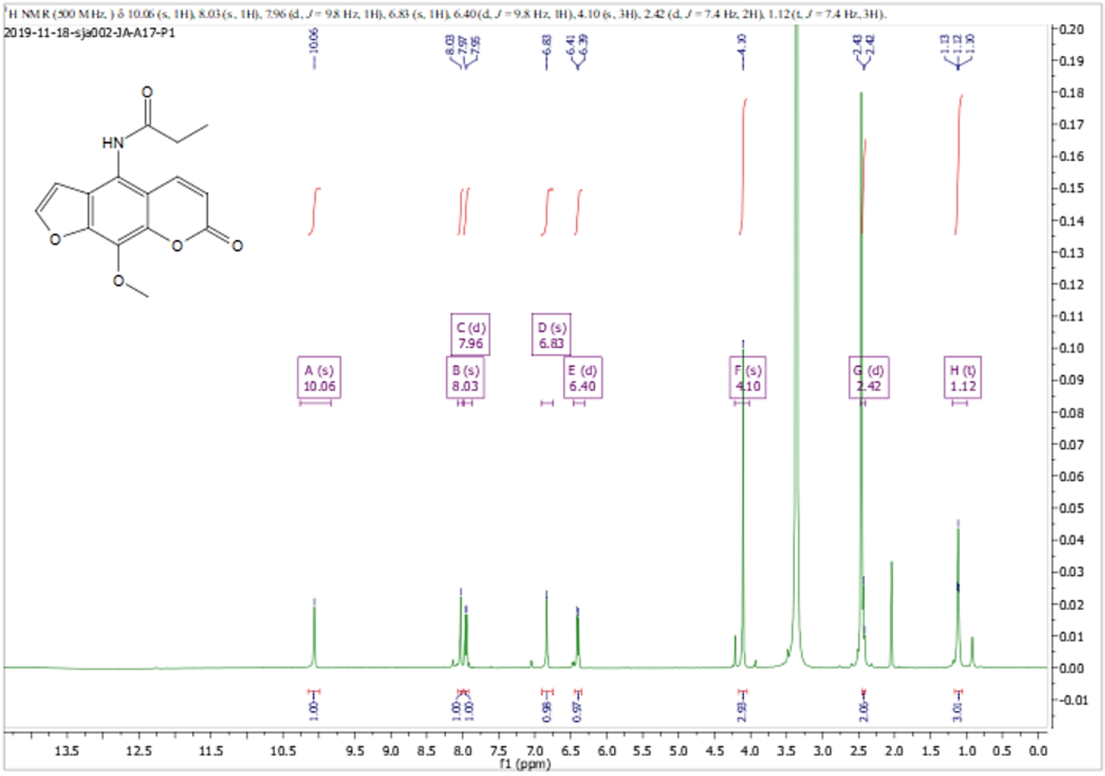

Figure S42 ^{1}H NMR spectrum of **3j**

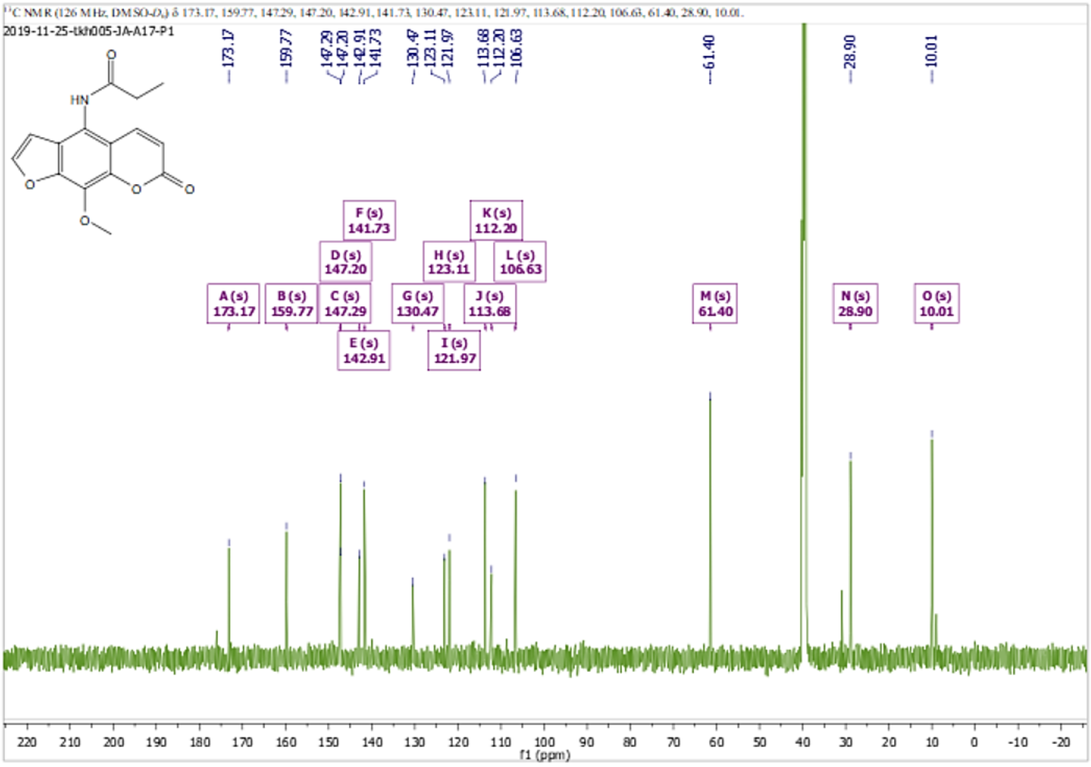

Figure S43 ^{13}C NMR spectrum of **3j**

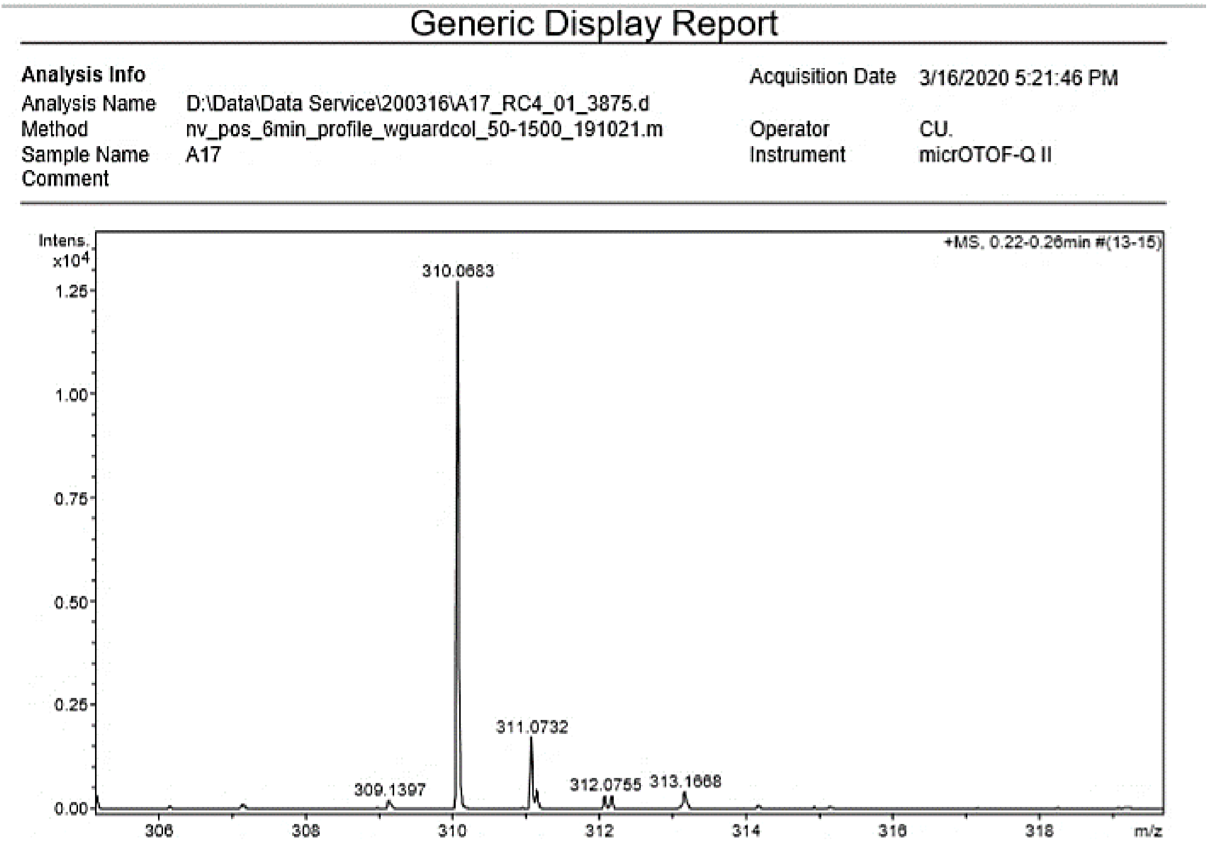

Figure S44 HRMS spectrum of **3j**

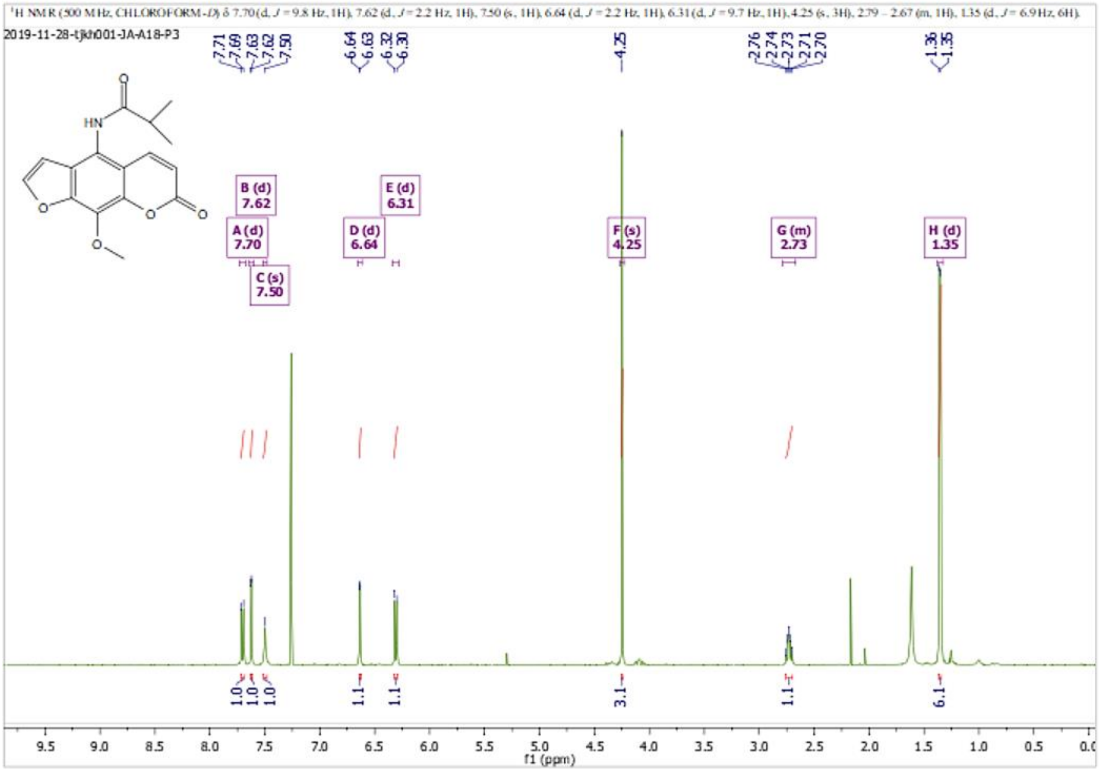

Figure S45 ^{1}H NMR spectrum of **3k**

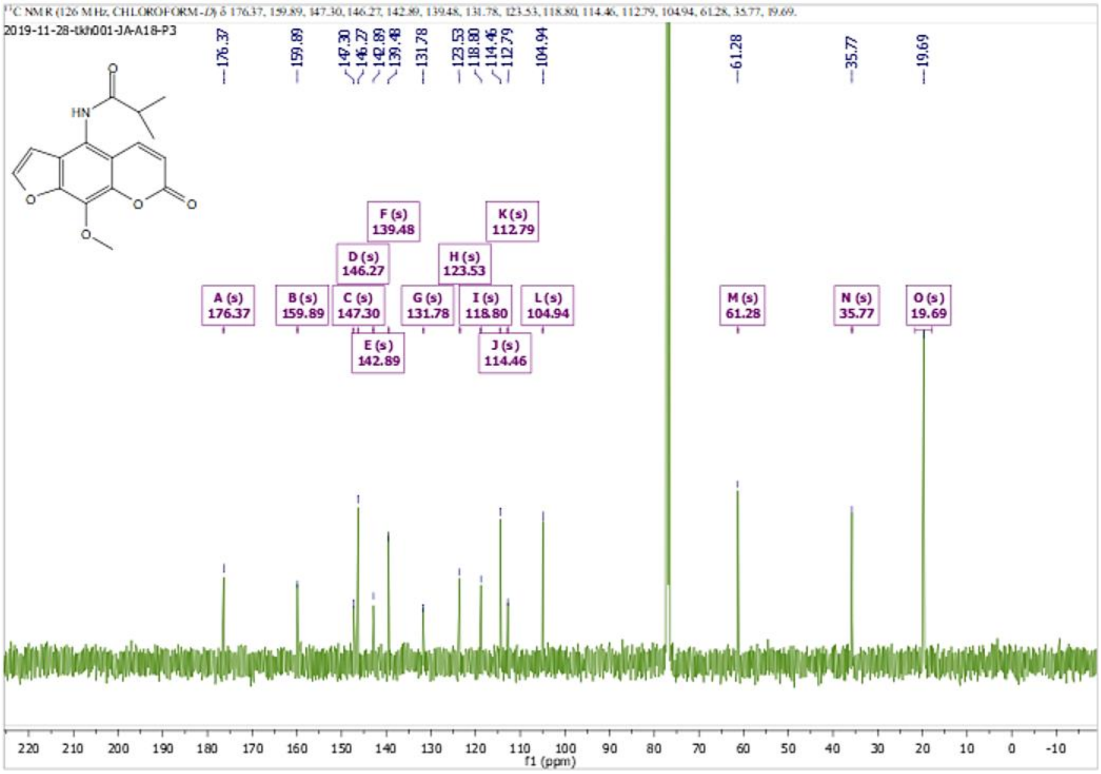

Figure S46 ^{13}C NMR spectrum of **3k**

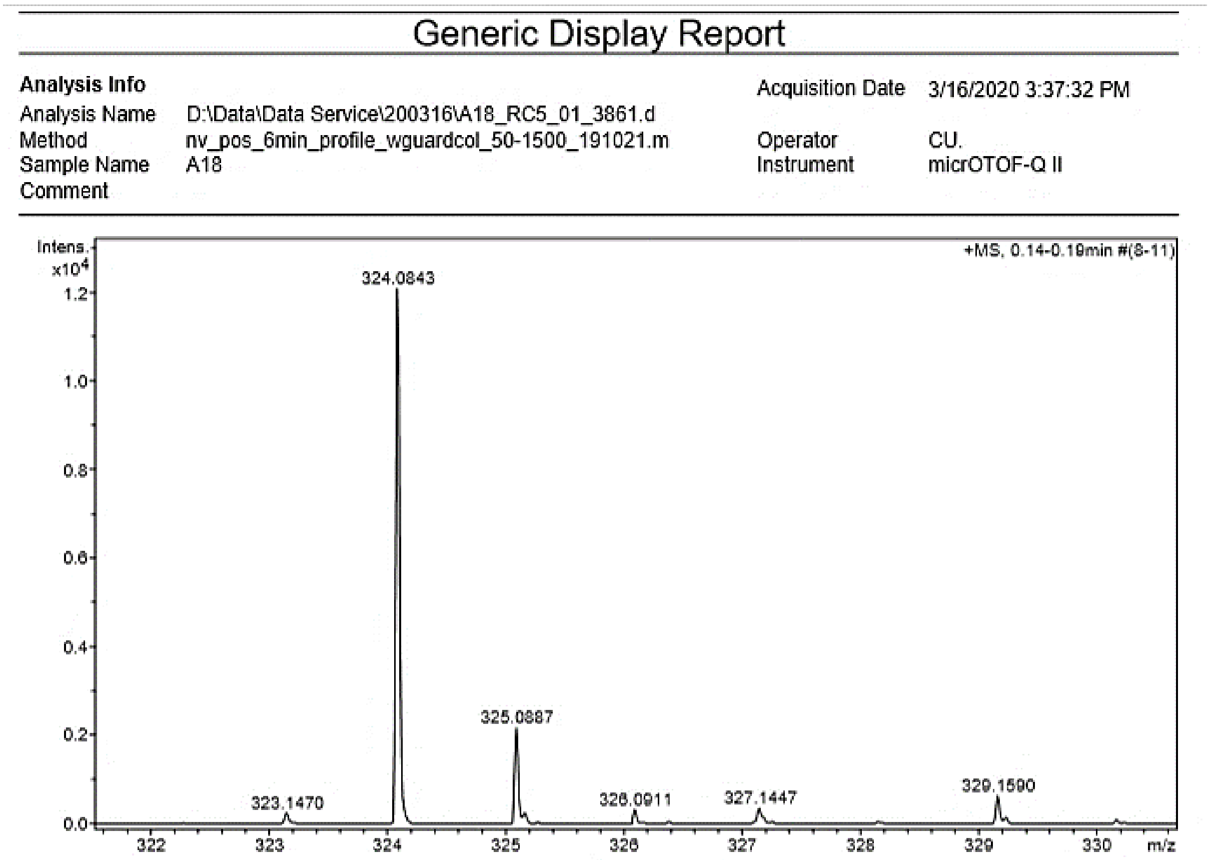

Figure S47 HRMS spectrum of **3k**

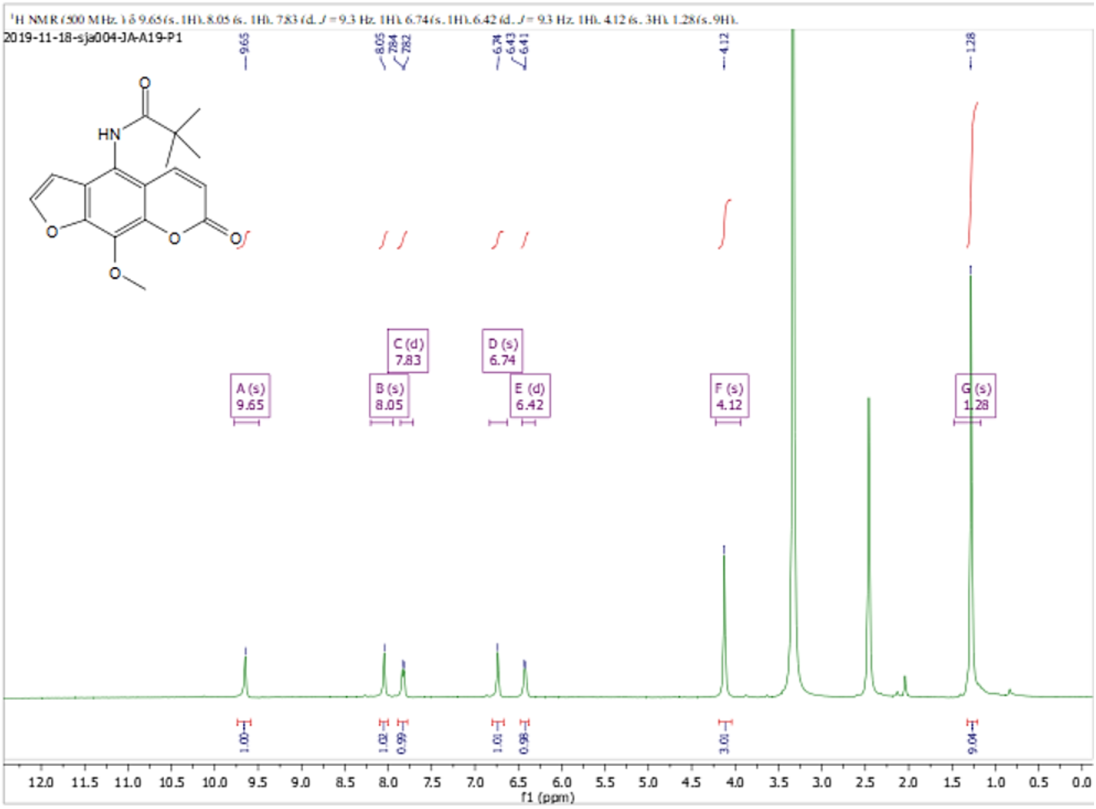

Figure S48 ^{1}H NMR spectrum of **3l**

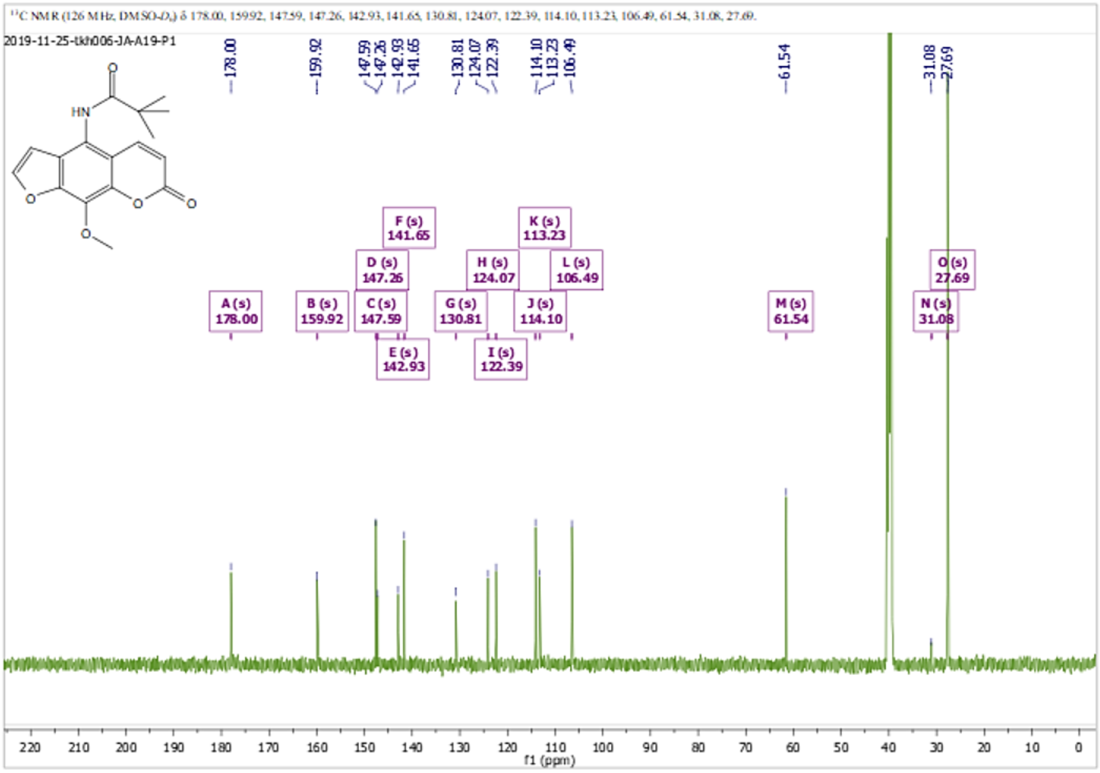

Figure S49 ^{13}C NMR spectrum of **3l**

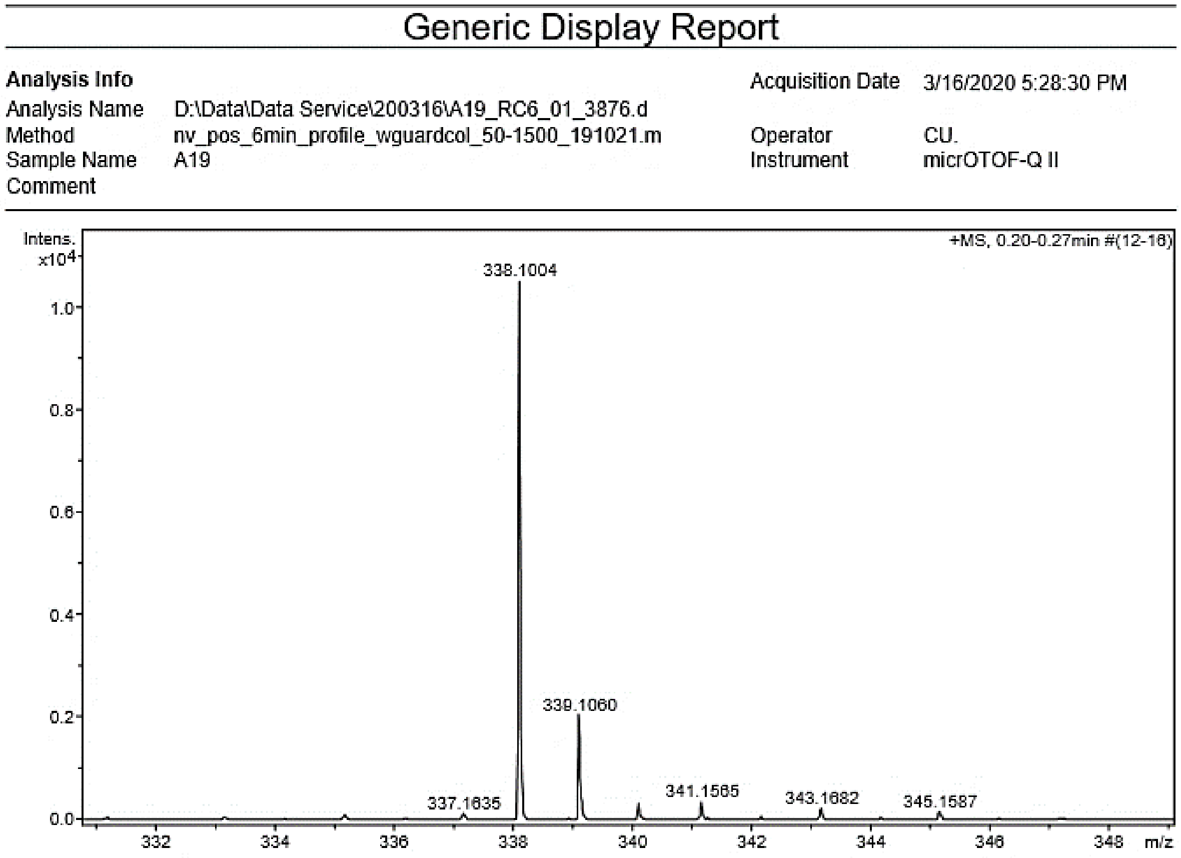

Figure S50 HRMS spectrum of **3l**

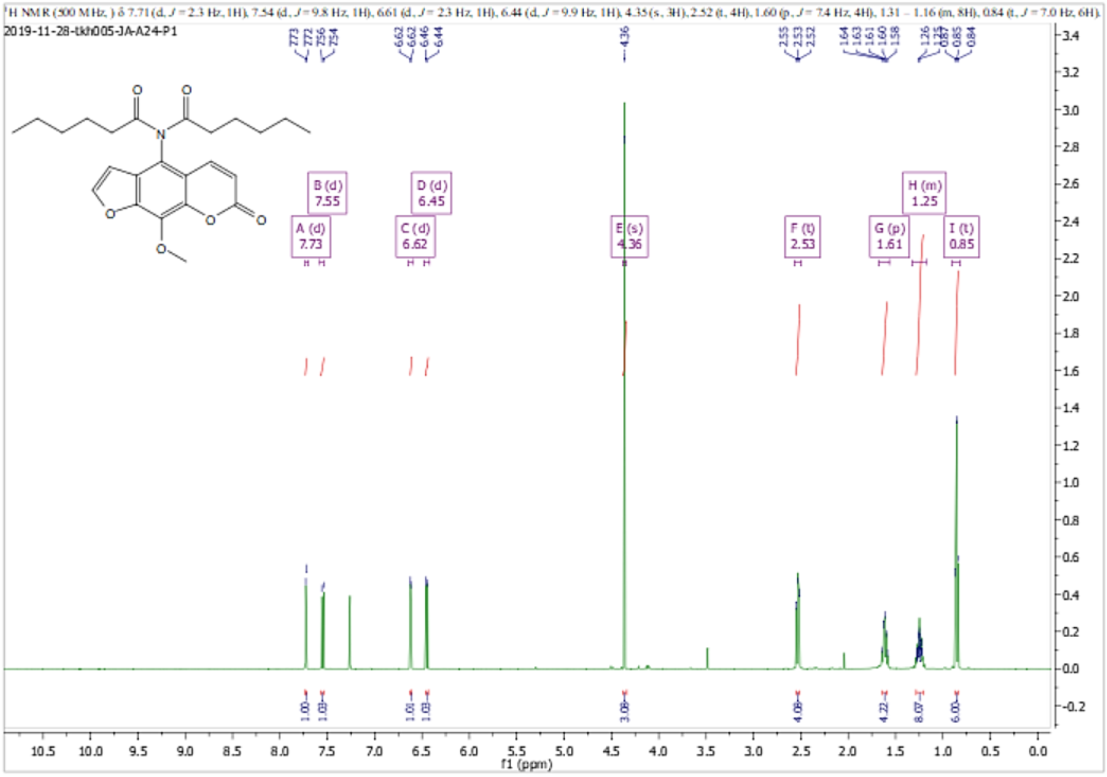

Figure S51 ^{1}H NMR spectrum of **3m**

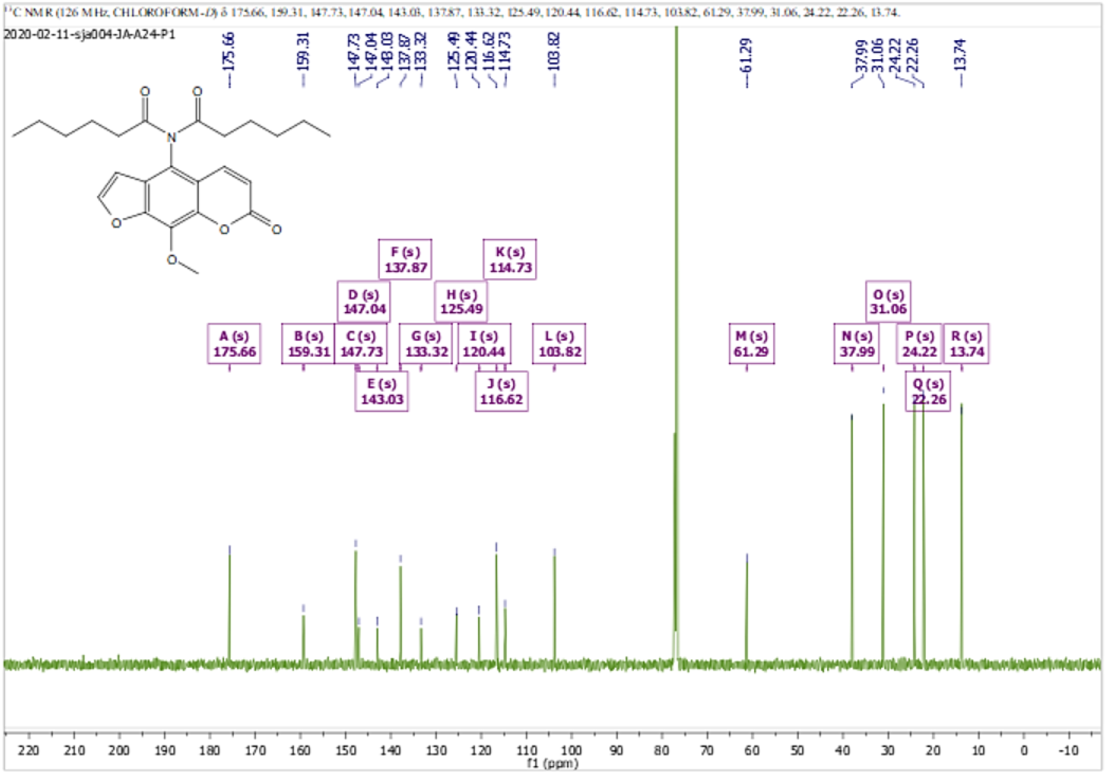

Figure S52 ^{13}C NMR spectrum of **3m**

Generic Display Report

**Analysis Info**		Acquisition Date	3/16/2020 5:42:02 PM
Analysis Name	D:\Data\Data Service\200316\A24_RC8_01_3878.d		
Method	nv_pos_6min_profile_wguardcol_50-1500_191021.m	Operator	CU.
Sample Name	A24	Instrument	micrOTOF-Q II
Comment			

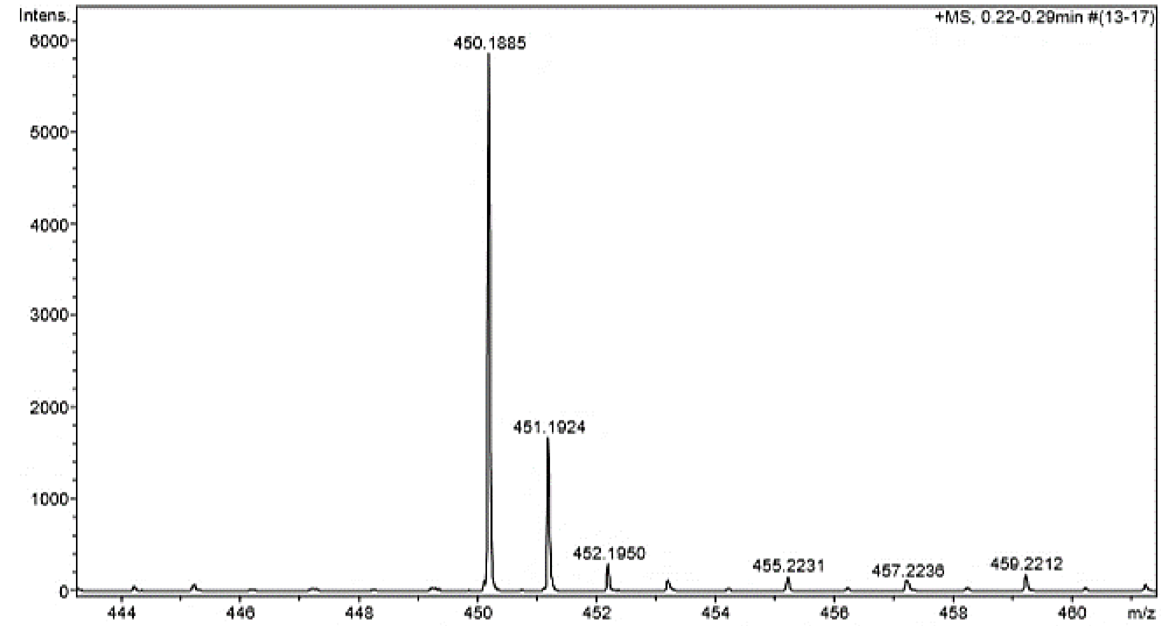

Figure S53 HRMS spectrum of **3m**

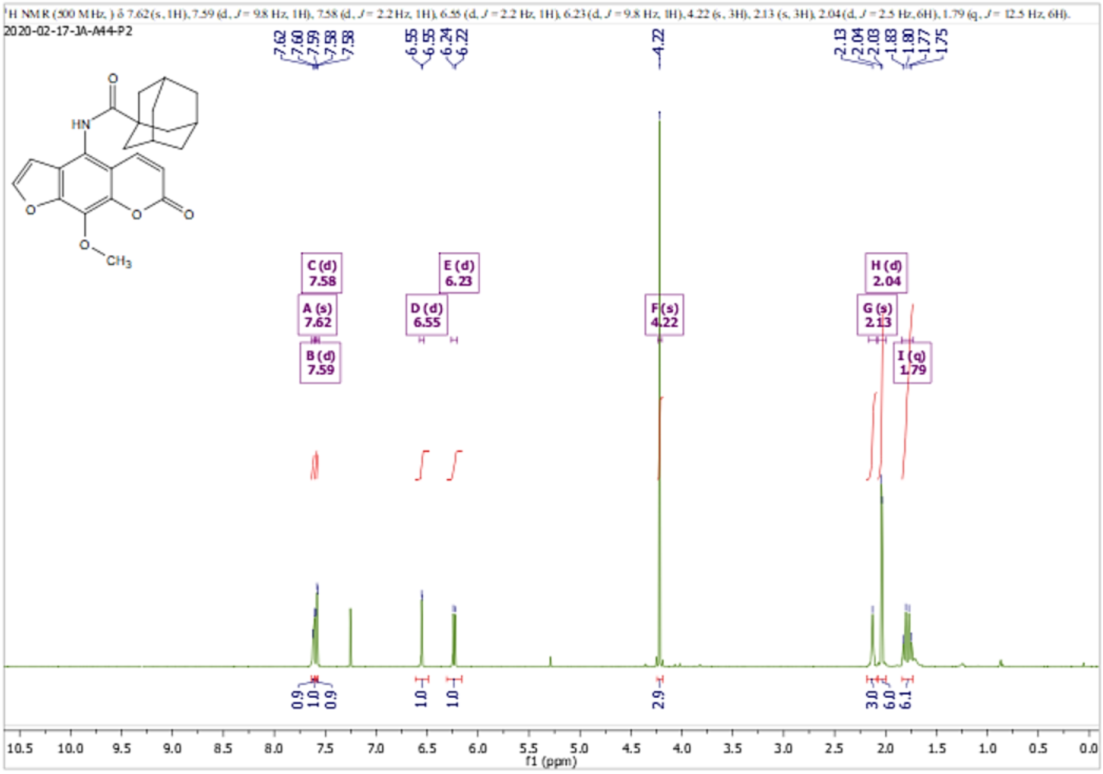

Figure S54 ^{1}H NMR spectrum of **3n**

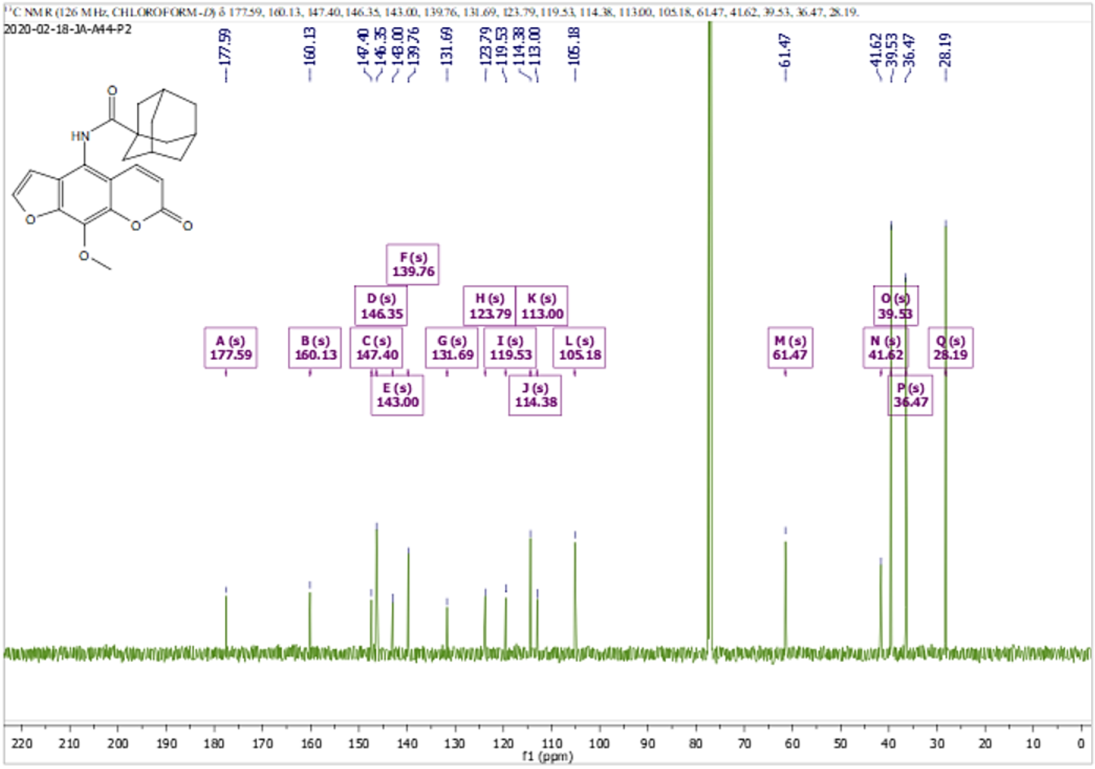

Figure S55 ^{13}C NMR spectrum of **3n**

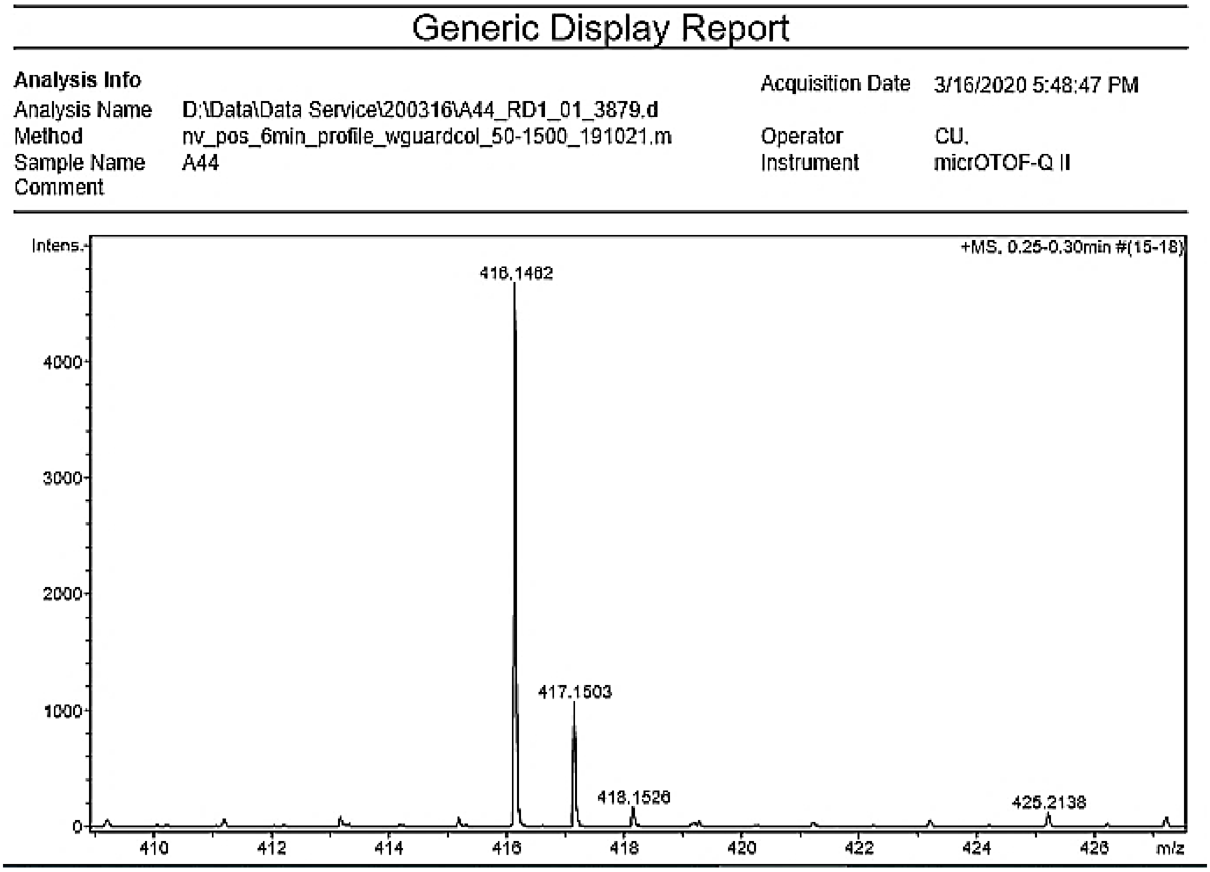

Figure S56 HRMS spectrum of **3n**

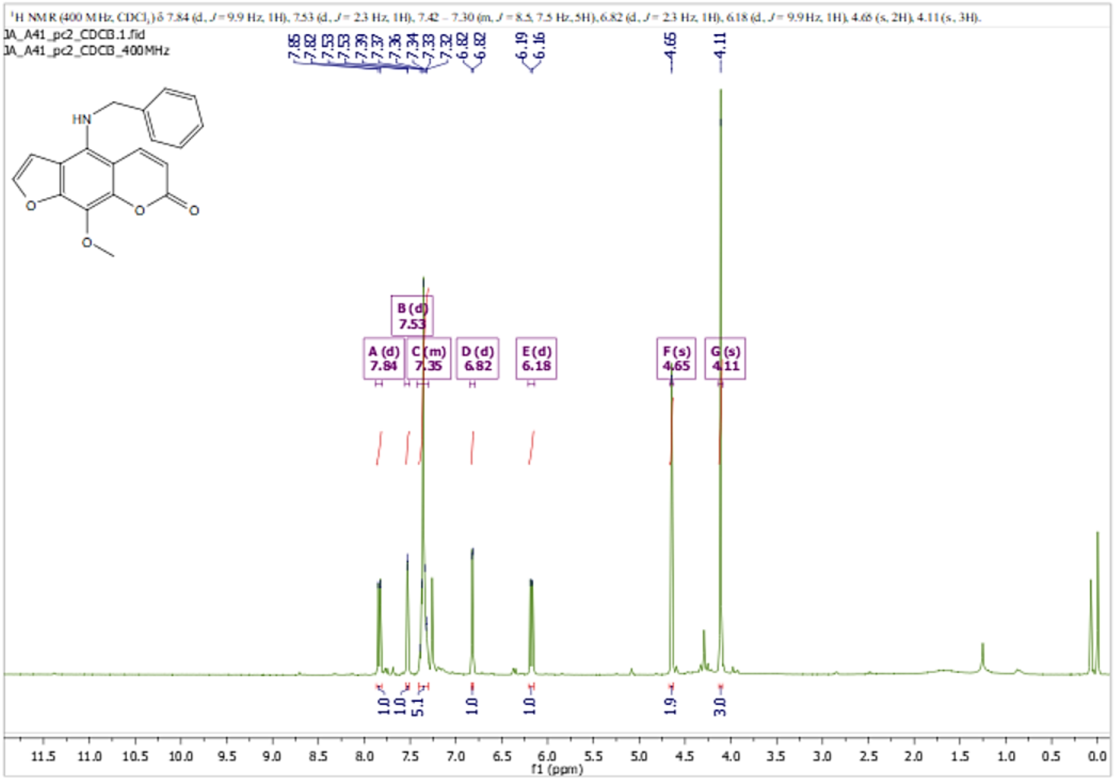

Figure S57 ^{1}H NMR spectrum of **4a**

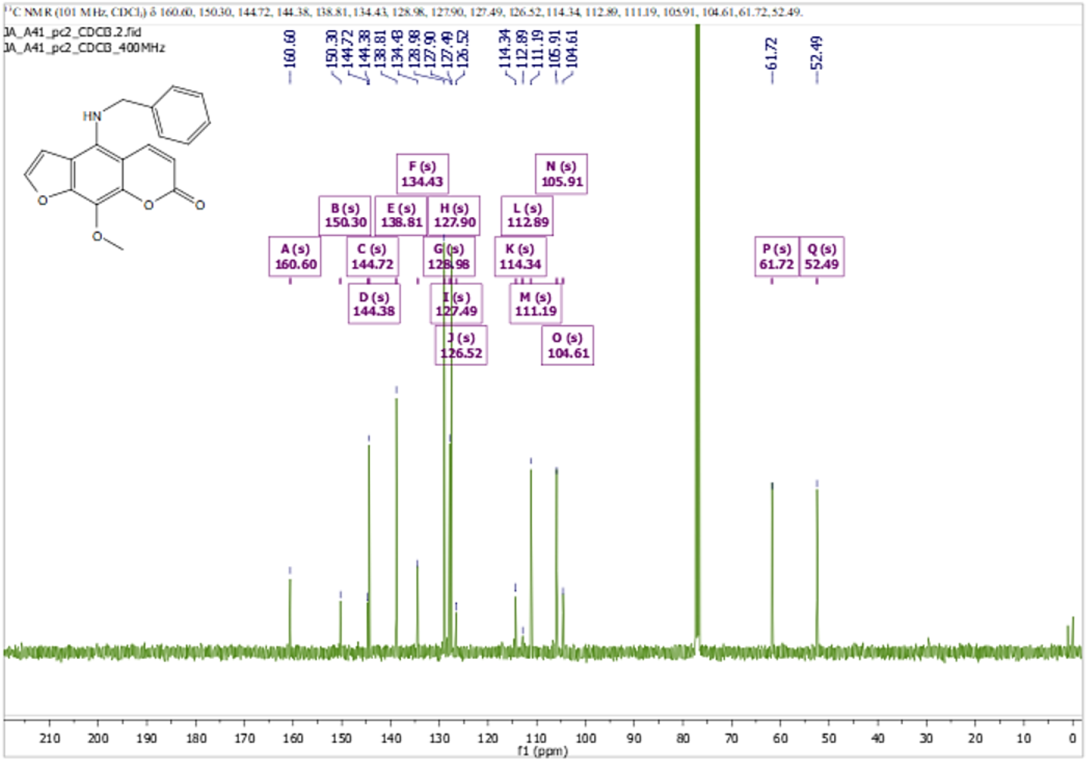

Figure S58 ^{13}C NMR spectrum of **4a**

Mass Spectrum List Report

Analysis Info

Analysis Name	TOFCRI25780 Jutatip JA741-PC2 E+.d	Acquisition Date	12/27/2019 1:54:37 AM
Method	Nitirat ESI pos 2019-1.m	Operator	Administrator
Sample Name	ESIpos	Instrument	micrOTOF 74

Acquisition Parameter

Source Type	ESI	Ion Polarity	Positive	Set Corrector Fill	64 V
Scan Range	n/a	Capillary Exit	110.0 V	Set Pulsar Pull	405 V
Scan Begin	100 m/z	Hexapole RF	160.0 V	Set Pulsar Push	405 V
Scan End	850 m/z	Skimmer 1	33.0 V	Set Reflector	1300 V
		Hexapole 1	22.9 V	Set Flight Tube	9000 V
				Set Detector TOF	1988 V

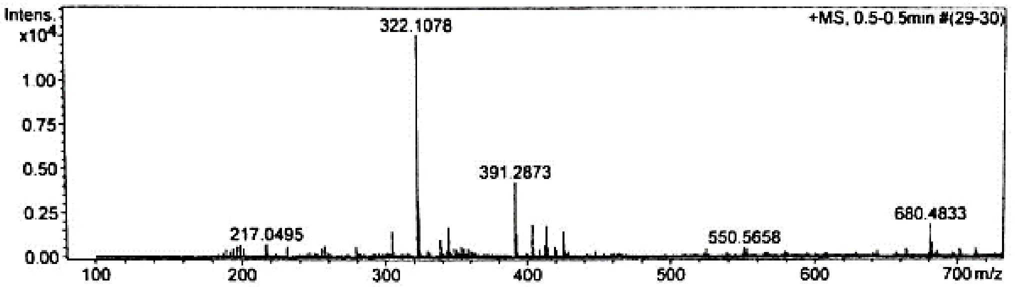

Figure S59 HRMS spectrum of **4a**

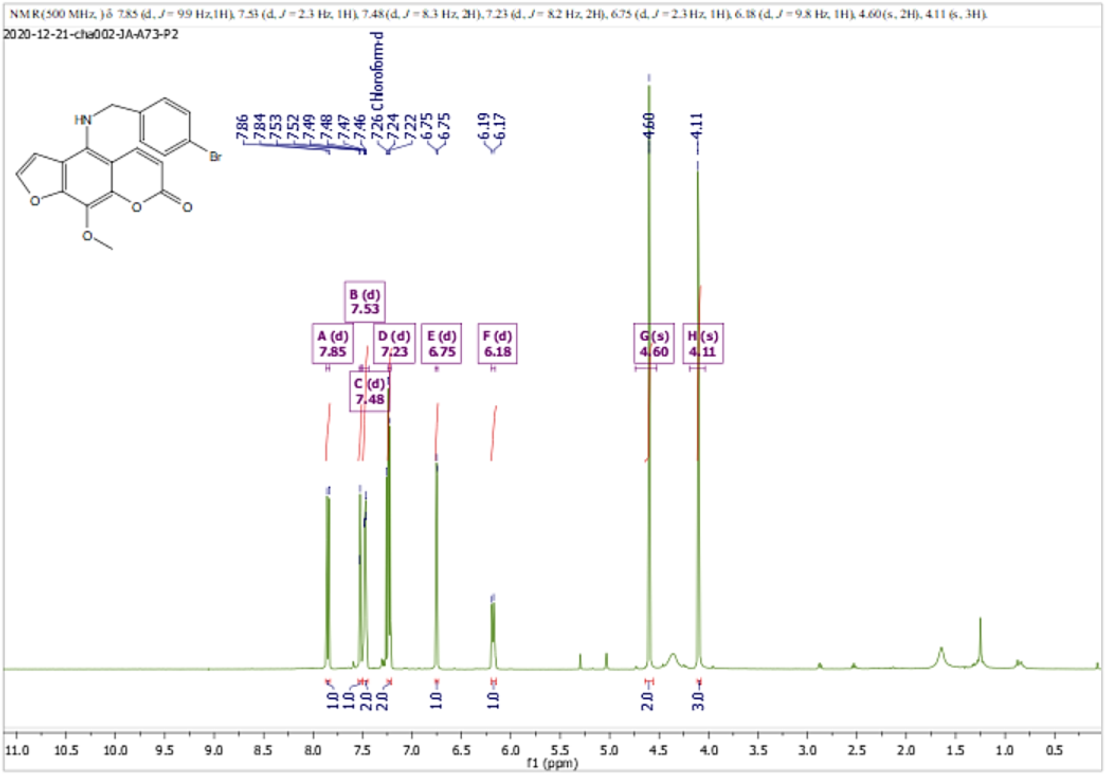

Figure S60 ^{1}H NMR spectrum of **4b**

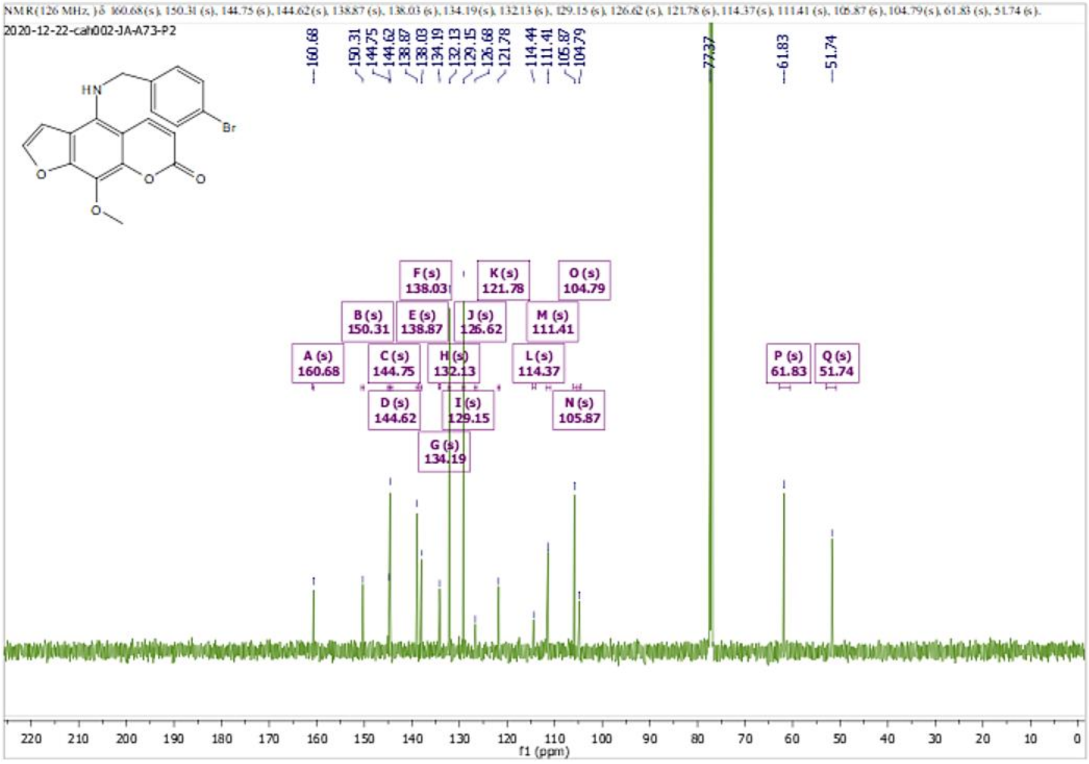

Figure S61 ^{13}C NMR spectrum of **4b**

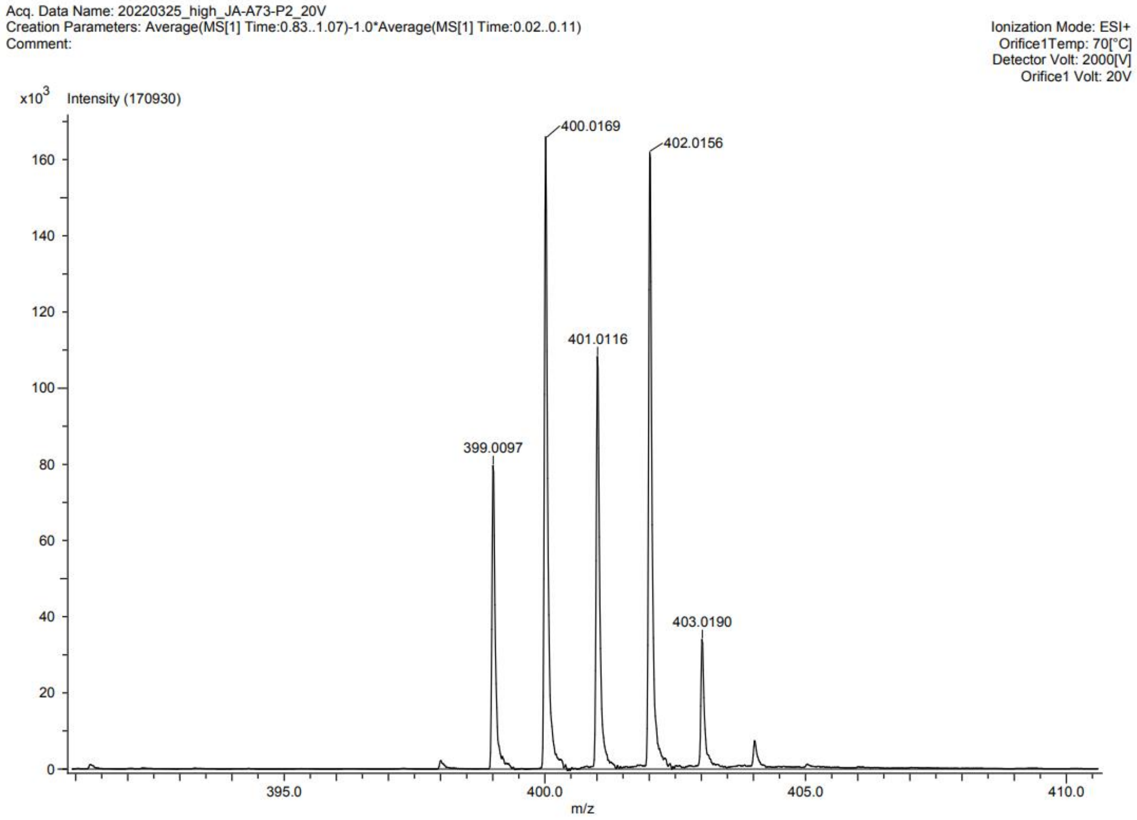

Figure S62 HRMS spectrum of **4b**

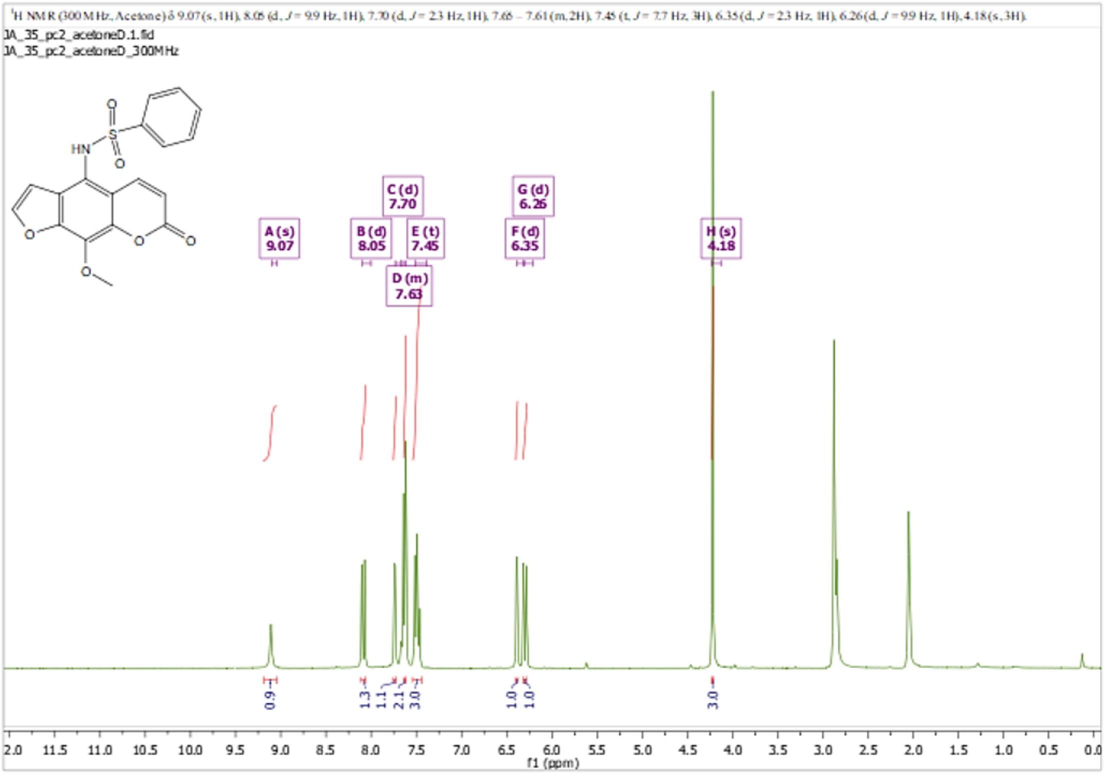

Figure S63 ^{1}H NMR spectrum of **5**

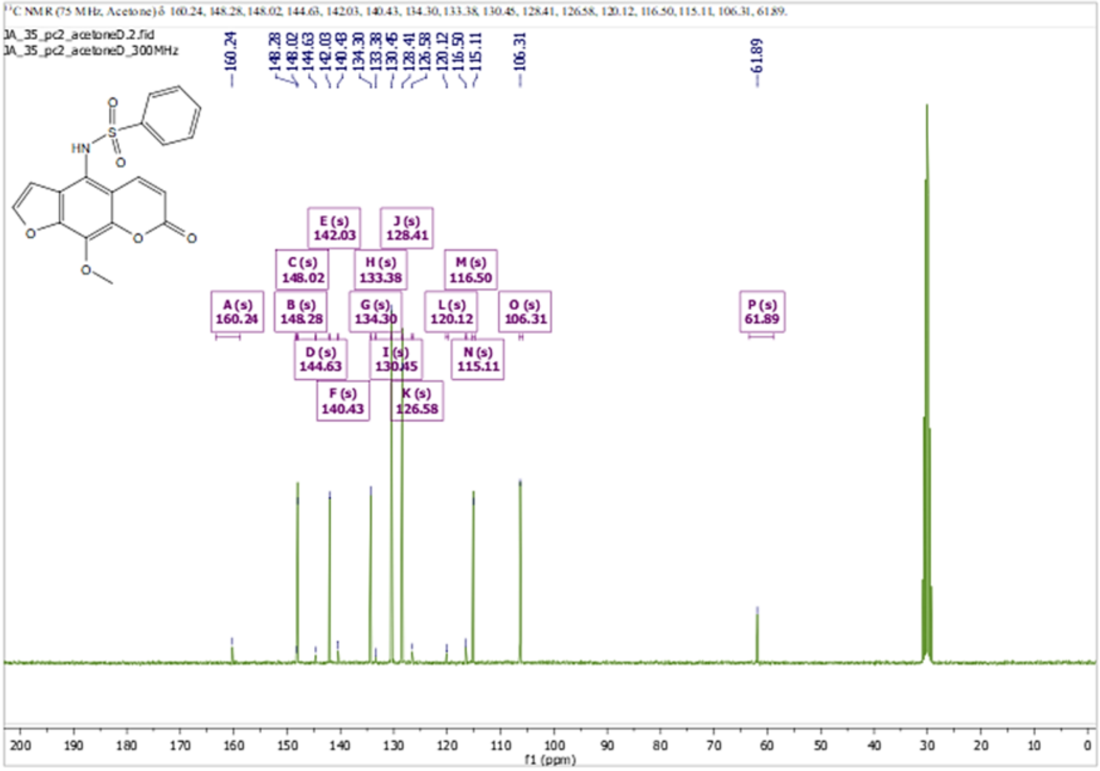

Figure S64 ^{13}C NMR spectrum of **5**

Mass Spectrum List Report

Analysis Info

Analysis Name	TOFCRI25770 Jutatip JAA36-PC2 E+.d	Acquisition Date	12/23/2019 1:34:51 AM
Method	Nitirat ESI pos 2019-1.m	Operator	Administrator
Sample Name	ESIpos	Instrument	micrOTOF 74

Acquisition Parameter

Source Type	ESI	Ion Polarity	Positive	Set Corrector Fill	64 V
Scan Range	n/a	Capillary Exit	110.0 V	Set Pulsar Pull	405 V
Scan Begin	100 m/z	Hexapole RF	160.0 V	Set Pulsar Push	405 V
Scan End	850 m/z	Skimmer 1	33.0 V	Set Reflector	1300 V
		Hexapole 1	22.9 V	Set Flight Tube	9000 V
				Set Detector TOF	1988 V

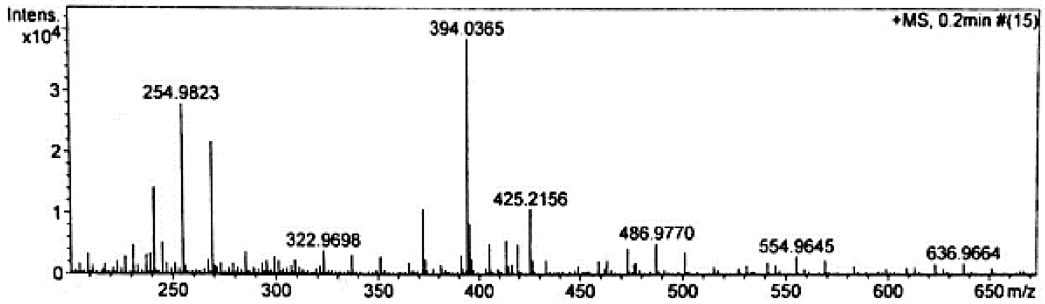

Figure S65 HRMS spectrum of **5**

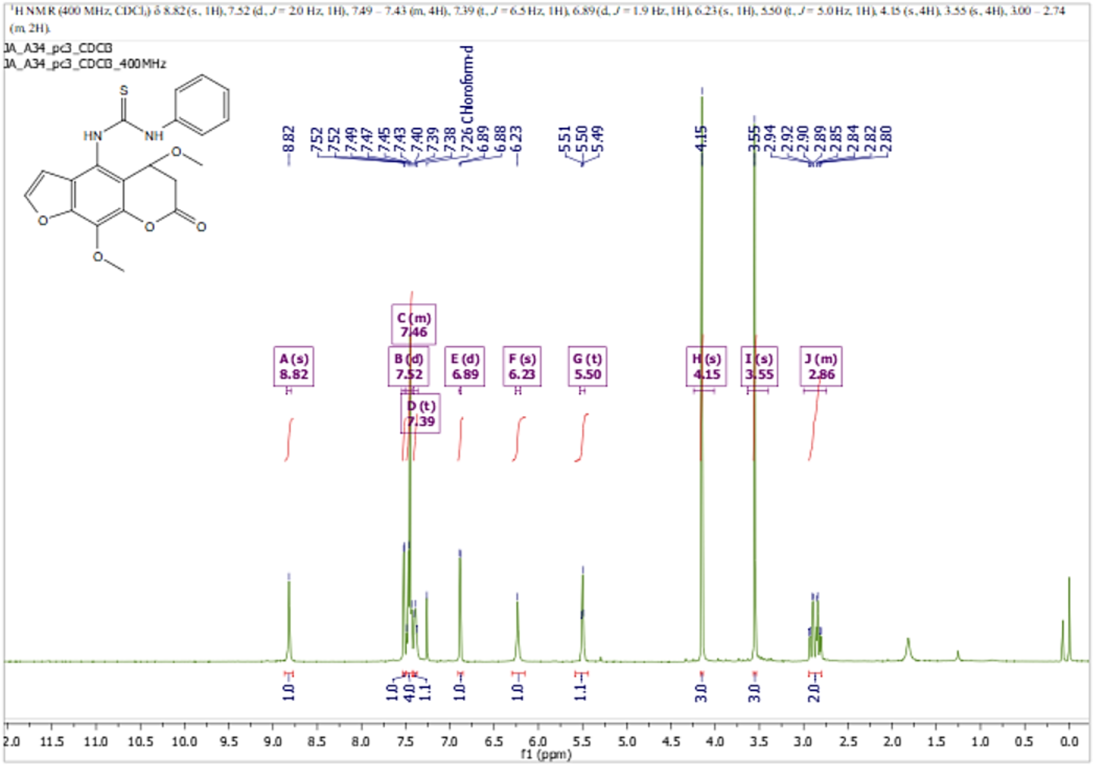

Figure S66 ^{1}H NMR spectrum of **6**

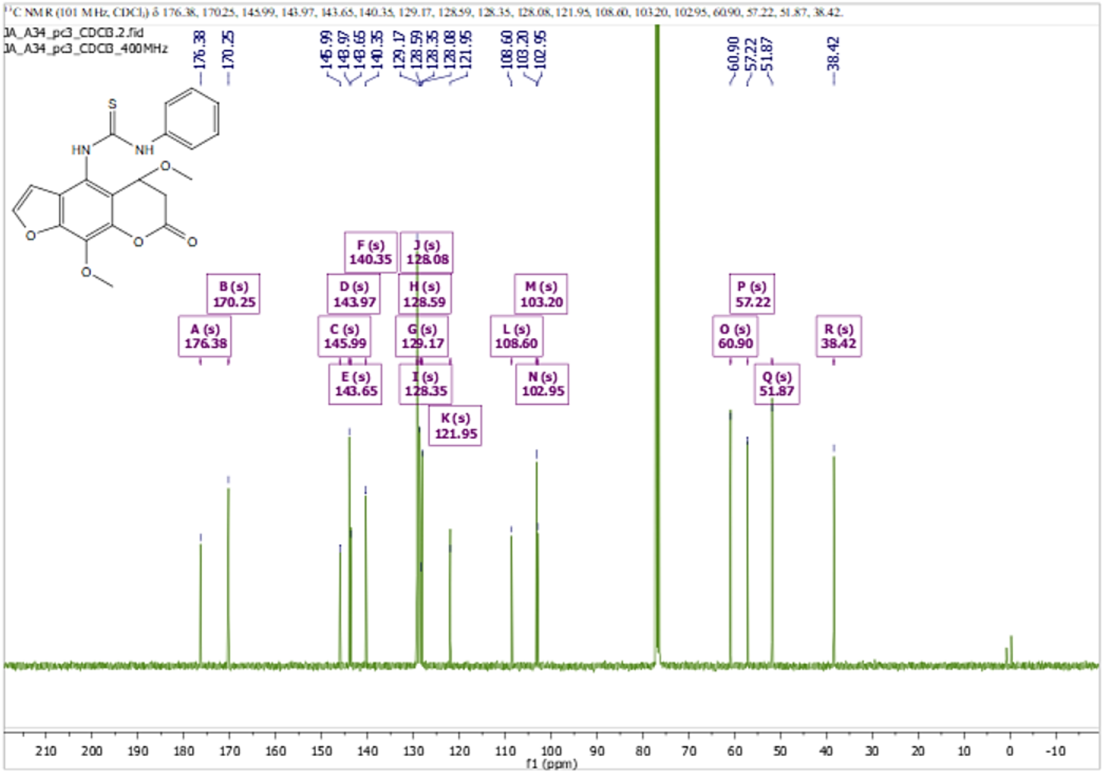

Figure S67 ^{13}C NMR spectrum of **6**

Mass Spectrum List Report

Analysis Info

Analysis Name	TOFCRI25777 Jutatip JAA34-PC3 E+.d	Acquisition Date	12/25/2019 12:46:05 AM
Method	Nitirat ESI pos 2019-1.m	Operator	Administrator
Sample Name	ESIpos	Instrument	micrOTOF 74

Acquisition Parameter

Source Type	ESI	Ion Polarity	Positive	Set Corrector Fill	64 V
Scan Range	n/a	Capillary Exit	110.0 V	Set Pulsar Pull	405 V
Scan Begin	100 m/z	Hexapole RF	160.0 V	Set Pulsar Push	405 V
Scan End	850 m/z	Skimmer 1	33.0 V	Set Reflector	1300 V
		Hexapole 1	22.9 V	Set Flight Tube	9000 V
				Set Detector TOF	1988 V

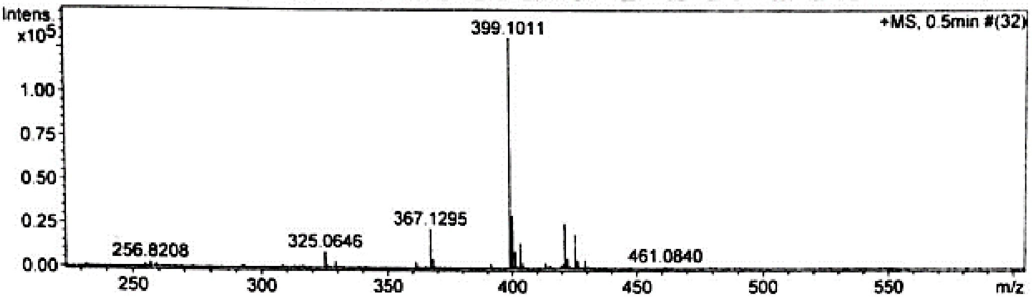

Figure S68 HRMS spectrum of **6**

6. HPLC spectra

SAMPLE INFORMATION

Sample Name:	JA-A01-P1	Acquired By:	natcri
Sample Type:	Unknown	Sample Set Name:	08032022
Vial:	7	Acq. Method Set:	
Injection #:	1	Processing Method	JA_A01_P1_RT16
Injection Volume:	10.00 ul	Channel Name:	254.0nm@2
Run Time:	80.0 Minutes	Proc. Chnl. Descr.:	2998 PDA 254.0 nm (2998
Date Acquired:	08-Mar-22 19:25:45 ICT		
Date Processed:	08-Mar-22 20:31:17 ICT		

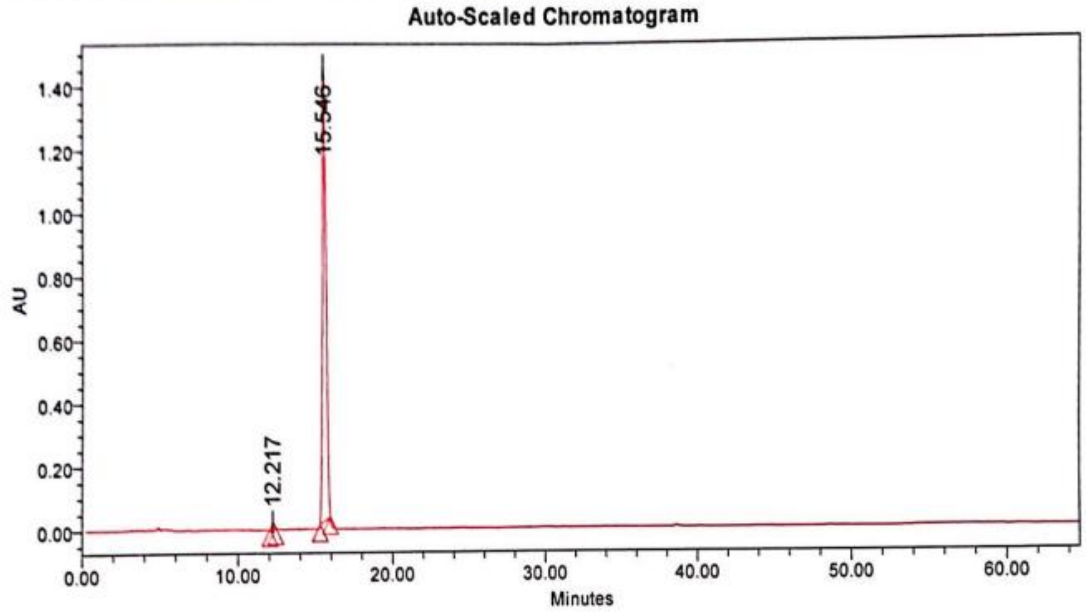

Peak Summary with Statistics
Name:

	Sample Name	Vial	Inj	Retention Time (min)	Area	% Area	Height
1	JA-A01-P1	7	1	15.546	23043402	99.11	1434921
2	JA-A01-P1	7	1	12.217	207470	0.89	16825
Mean				13.881			
Std. Dev.				2.354			

Figure S69 HPLC spectrum of **1**

SAMPLE INFORMATION

Sample Name:	JAA02P1	Acquired By:	natcri
Sample Type:	Unknown	Sample Set Name:	JAA02P1
Vial:	1	Acq. Method Set:	50_100_60m_100_20m_80m_Ana
Injection #:	1	Processing Method:	JAA02P1 06042022
Injection Volume:	10.00 ul	Channel Name:	254.0nm
Run Time:	80.0 Minutes	Proc. Chnl. Descr.:	PDA 254.0 nm

Date Acquired: 06/04/2022 11:16:23 AM ICT
Date Processed: 06/04/2022 12:19:27 PM ICT

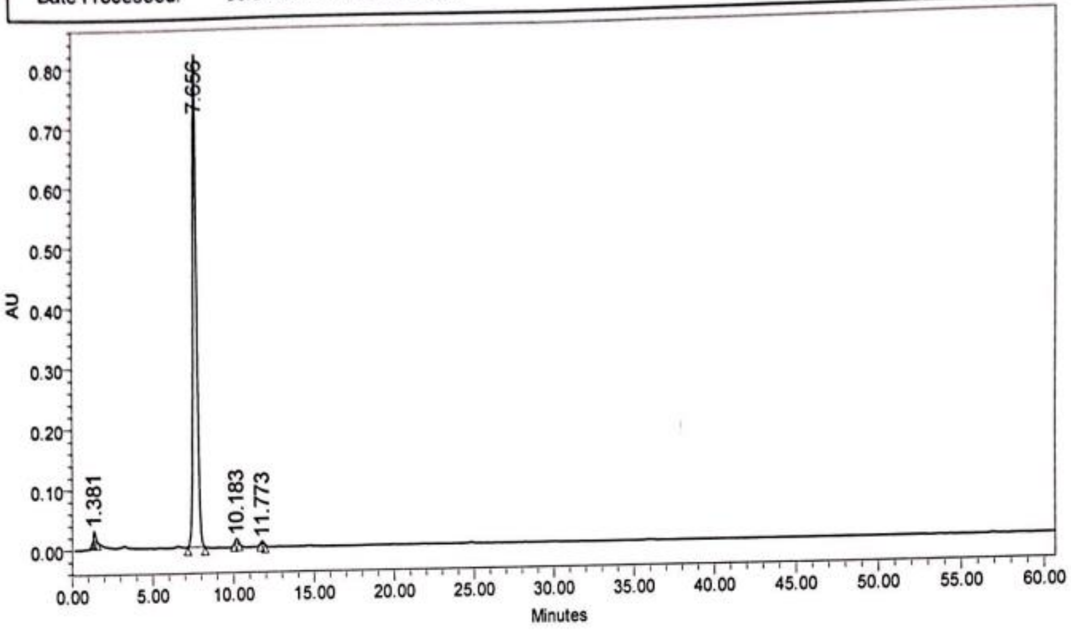

	RT	Area	% Area	Height
1	1.381	125723	0.95	16356
2	7.656	12960404	98.12	818805
3	10.183	81347	0.62	7020
4	11.773	40955	0.31	3881

Figure S70 HPLC spectrum of **2**

SAMPLE INFORMATION

Sample Name:	JAA03P1	Acquired By:	natcri
Sample Type:	Unknown	Sample Set Name:	JAA03P1_09032022
Vial:	2	Acq. Method Set:	50_100_60m_100_20m_80m_Ana
Injection #:	1	Processing Method:	JAA03P1 1
Injection Volume:	10.00 ul	Channel Name:	254.0nm
Run Time:	80.0 Minutes	Proc. Chnl. Descr.:	PDA 254.0 nm
Date Acquired:	09/03/2022 11:51:51 AM ICT		
Date Processed:	09/03/2022 12:55:15 PM ICT		

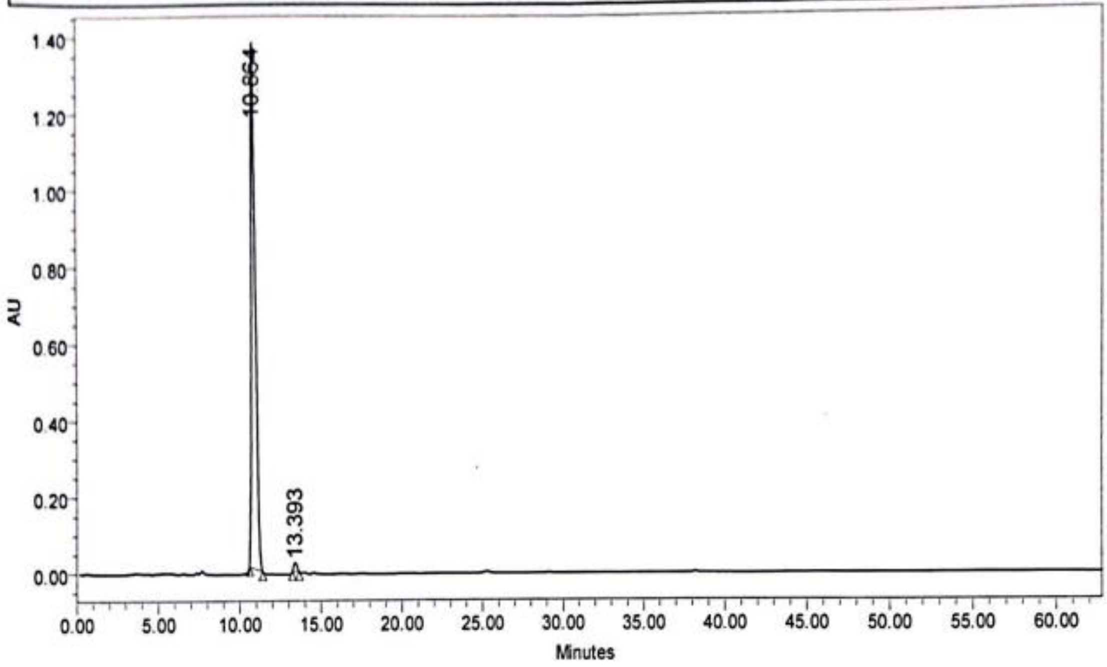

	RT	Area	% Area	Height
1	10.864	23931504	98.67	1371477
2	13.393	323401	1.33	22900

Figure S71 HPLC spectrum of **3a**

SAMPLE INFORMATION

Sample Name:	JAA04P1	Acquired By:	natcri
Sample Type:	Unknown	Sample Set Name:	JAA04P1_09032022
Vial:	4	Acq. Method Set:	50_100_60m_100_20m_80m_Ana
Injection #:	1	Processing Method:	JAA04P1 09032022
Injection Volume:	10.00 ul	Channel Name:	254.0nm
Run Time:	80.0 Minutes	Proc. Chnl. Descr.:	PDA 254.0 nm
Date Acquired:	09/03/2022 3:26:43 PM ICT		
Date Processed:	09/03/2022 4:31:33 PM ICT		

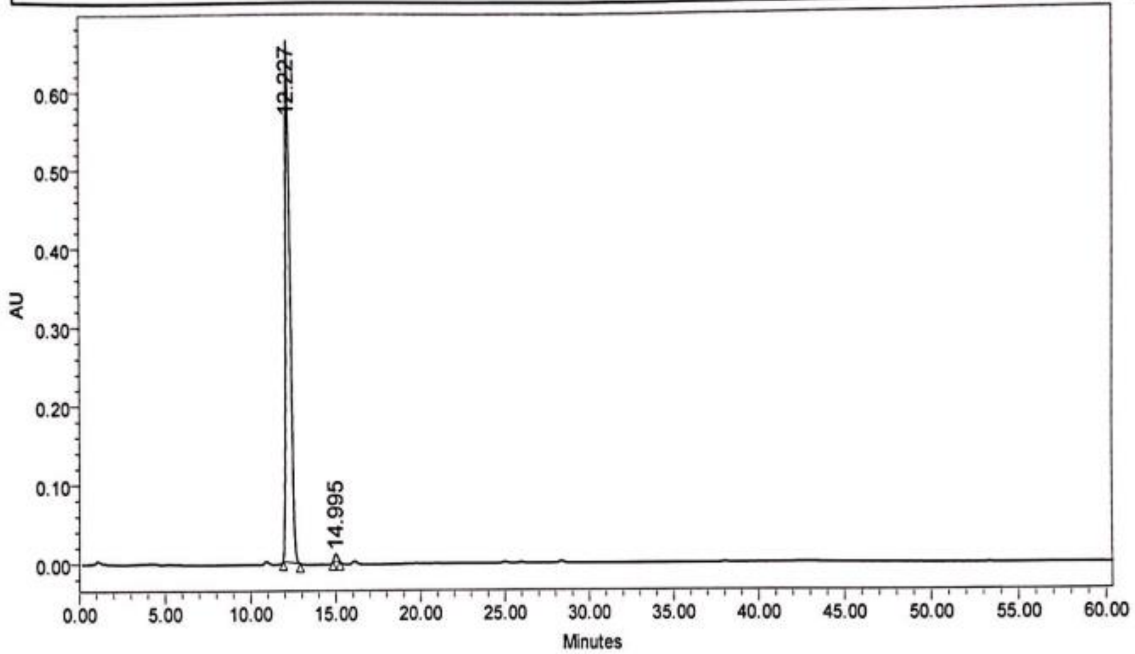

	RT	Area	% Area	Height
1	12.227	12206127	98.91	662490
2	14.995	134614	1.09	9304

Figure S72 HPLC spectrum of **3b**

SAMPLE INFORMATION

Sample Name:	JAA07P1	Acquired By:	natcri
Sample Type:	Unknow n	Sample Set Name:	JAA07P1
Vial:	1	Acq. Method Set:	50_100_60m_100_20m_80m_Ana
Injection #:	1	Processing Method:	JAA07P1 010032022
Injection Volume:	10.00 ul	Channel Name:	254.0nm@1
Run Time:	80.0 Minutes	Proc. Chnl. Descr.:	PDA 254.0 nm
Date Acquired:	10/03/2022 9:17:31 AM ICT		
Date Processed:	10/03/2022 10:18:40 AM ICT		

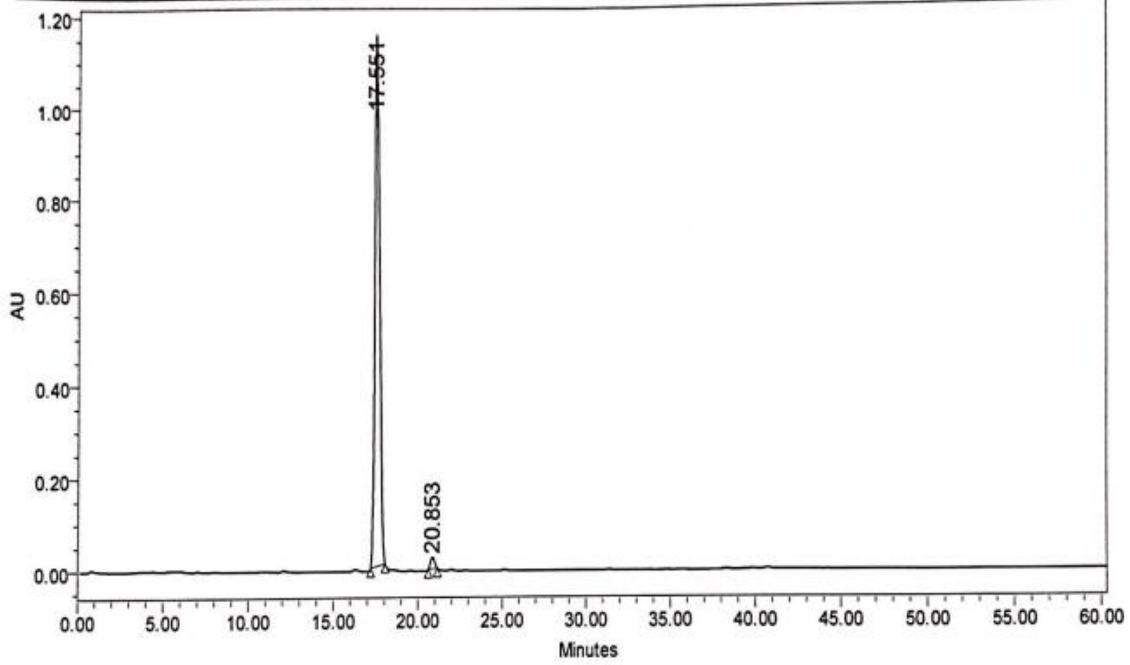

	RT	Area	% Area	Height
1	17.551	22520174	98.12	1149995
2	20.853	430905	1.88	25164

Figure S73 HPLC spectrum of **3c**

SAMPLE INFORMATION

Sample Name:	JAA05P1	Acquired By:	natcri
Sample Type:	Unknow n	Sample Set Name:	JAA04P1_09032022
Vial:	5	Acq. Method Set:	50_100_60m_100_20m_80m_Ana
Injection #:	1	Processing Method:	JAA05P1 09032022
Injection Volume:	10.00 ul	Channel Name:	254.0nm@1
Run Time:	80.0 Minutes	Proc. Chnl. Descr.:	PDA 254.0 nm
Date Acquired:	09/03/2022 4:48:31 PM ICT		
Date Processed:	09/03/2022 5:50:12 PM ICT		

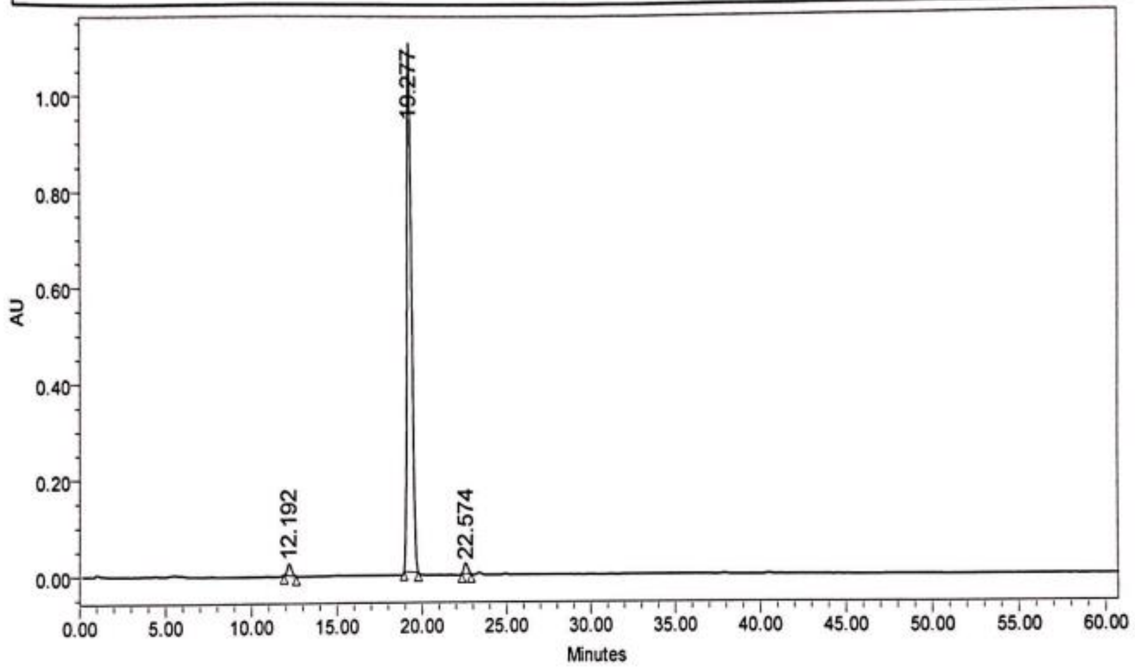

	RT	Area	% Area	Height
1	12.192	413766	1.95	23666
2	19.277	20410977	96.30	1102930
3	22.574	369811	1.74	22031

Figure S74 HPLC spectrum of **3d**

SAMPLE INFORMATION

Sample Name:	JAA20P1	Acquired By:	natcri
Sample Type:	Unknown	Sample Set Name:	JAA20P1
Vial:	2	Acq. Method Set:	50_100_60m_100_20m_80m_Ana
Injection #:	1 26	Processing Method:	JAA20P1 1 11032022
Injection Volume:	10.00 ul	Channel Name:	254.0nm
Run Time:	80.0 Minutes	Proc. Chnl. Descr.:	PDA 254.0 nm

Date Acquired: 11/03/2022 10:02:17 AM ICT
Date Processed: 11/03/2022 11:03:28 AM ICT

②

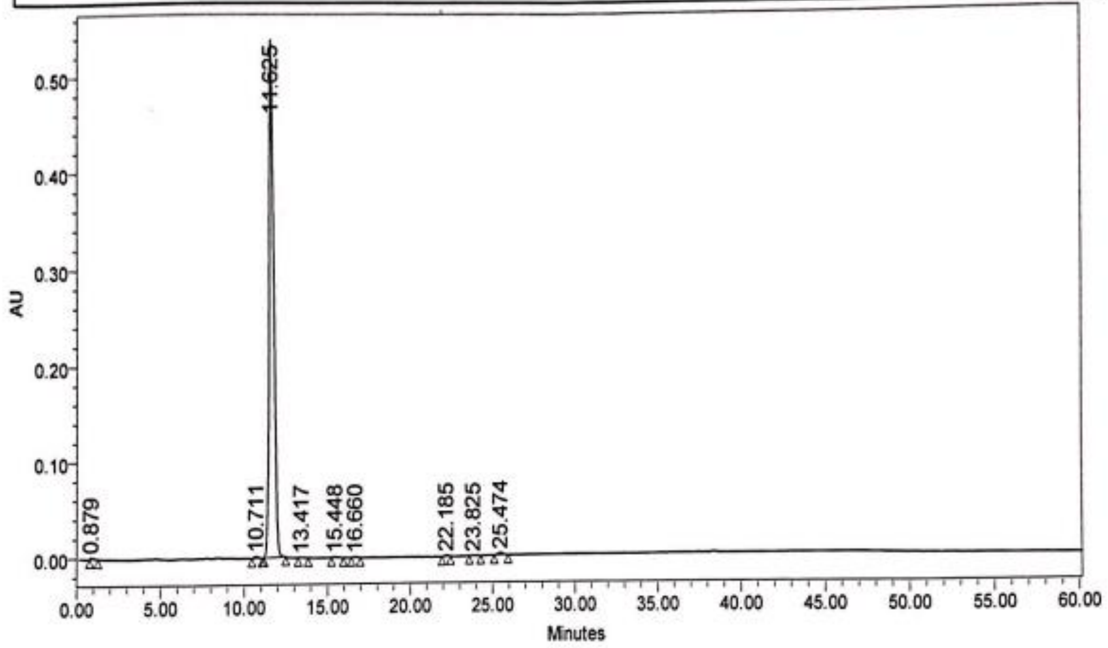

	RT	Area	% Area	Height
1	0.879	23294	0.22	1612
2	10.711	36291	0.35	2181
3	11.625	10219088	98.31	540281
4	13.417	7689	0.07	507
5	15.448	16108	0.15	785
6	16.660	11742	0.11	726
7	22.185	20240	0.19	1296
8	23.825	8145	0.08	436
9	25.474	51801	0.50	2503

Figure S75 HPLC spectrum of **3e**

SAMPLE INFORMATION

Sample Name:	JAA66P1	Acquired By:	natcri
Sample Type:	Unknow n	Sample Set Name:	JAA66P1
Vial:	3	Acq. Method Set:	50_100_60m_100_20m_80m_Ana
Injection #:	1	Processing Method:	JAA66P1 18032022
Injection Volume:	10.00 ul	Channel Name:	254.0nm
Run Time:	80.0 Minutes	Proc. Chnl. Descr.:	PDA 254.0 nm

Date Acquired: 18/03/2022 11:14:42 AM ICT
Date Processed: 18/03/2022 12:21:50 PM ICT

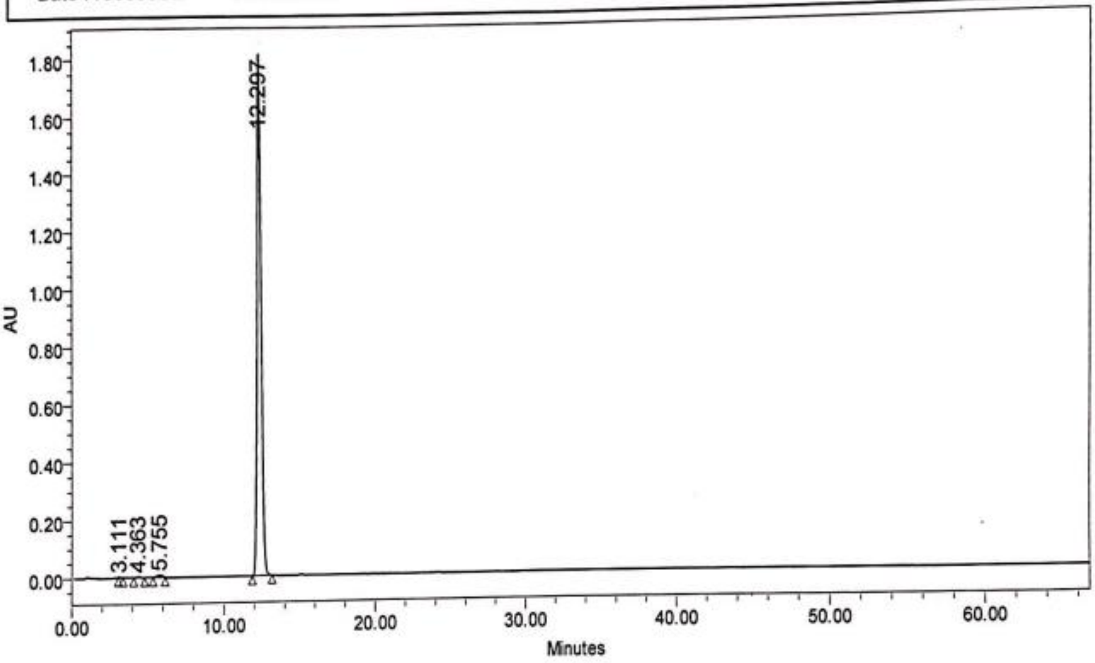

	RT	Area	% Area	Height
1	3.111	7734	0.02	1391
2	4.363	48525	0.15	3862
3	5.755	206375	0.62	8057
4	12.297	33108747	99.21	1816113

Figure S76 HPLC spectrum of **3f**

SAMPLE INFORMATION

Sample Name:	JAA15P1	Acquired By:	natcri
Sample Type:	Unknown	Sample Set Name:	JAA07P1
Vial:	2	Acq. Method Set:	50_100_60m_100_20m_80m_Ana
Injection #:	1	Processing Method:	JAA15P1 010032022
Injection Volume:	10.00 ul	Channel Name:	254.0nm@1
Run Time:	80.0 Minutes	Proc. Chnl. Descr.:	PDA 254.0 nm

①

Date Acquired: 10/03/2022 10:45:18 AM ICT
Date Processed: 10/03/2022 11:47:18 AM ICT

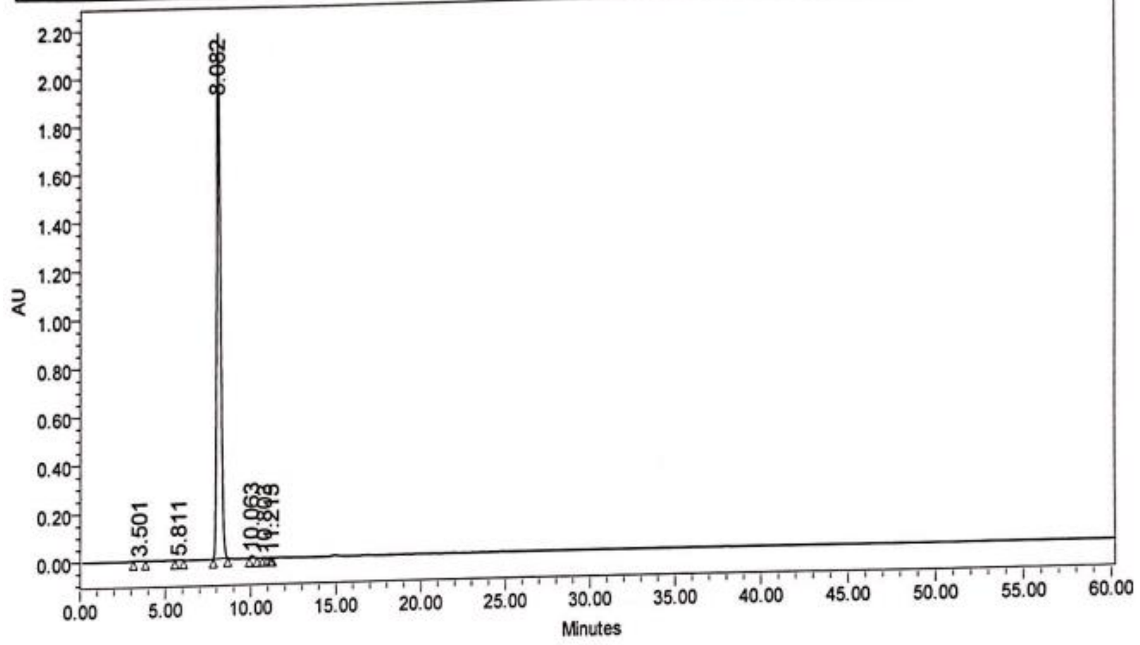

	RT	Area	% Area	Height
1	3.501	25219	0.08	1061
2	5.811	14172	0.04	785
3	8.082	32456556	99.45	2175253
4	10.063	128989	0.40	9268
5	10.803	10516	0.03	1139
6	11.215	1286	0.00	275

Figure S77 HPLC spectrum of **3g**

SAMPLE INFORMATION

Sample Name:	JAA16P1	Acquired By:	natcri
Sample Type:	Unknown	Sample Set Name:	JAA07P1
Vial:	5	Acq. Method Set:	50_100_60m_100_20m_80m_Ana
Injection #:	1	Processing Method:	JAA16P1 1 10032022
Injection Volume:	10.00 ul	Channel Name:	254.0nm
Run Time:	80.0 Minutes	Proc. Chnl. Descr.:	PDA 254.0 nm

Date Acquired: 10/03/2022 2:52:52 PM ICT
Date Processed: 10/03/2022 3:54:22 PM ICT

(2)

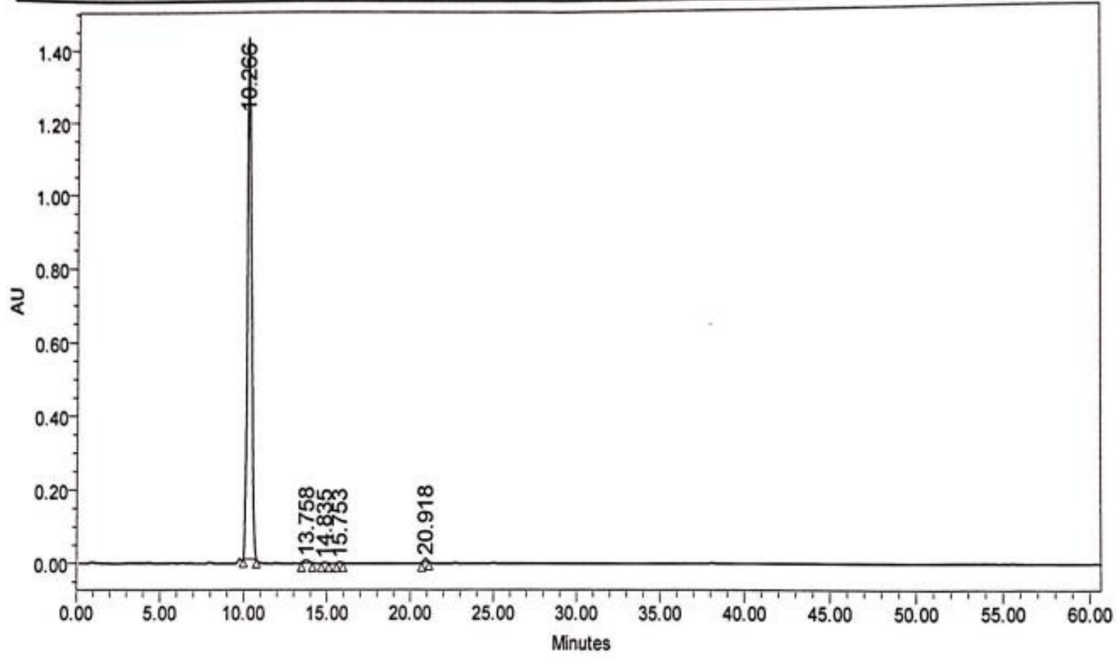

	RT	Area	% Area	Height
1	10.266	24162409	98.42	1419794
2	13.758	175724	0.72	7411
3	14.835	34552	0.14	2324
4	15.753	38185	0.16	2772
5	20.918	139157	0.57	9322

Figure S78 HPLC spectrum of **3h**

SAMPLE INFORMATION

Sample Name:	JAA12P1	Acquired By:	natcri
Sample Type:	Unknow n	Sample Set Name:	JAA12P1
Vial:	1	Acq. Method Set:	50_100_60m_100_20m_80m_Ana
Injection #:	1	Processing Method:	JAA12P1 13032022
Injection Volume:	10.00 ul	Channel Name:	254.0nm
Run Time:	80.0 Minutes	Proc. Chnl. Descr.:	PDA 254.0 nm

①

Date Acquired: 13/03/2022 10:05:34 AM ICT
Date Processed: 13/03/2022 11:12:32 AM ICT

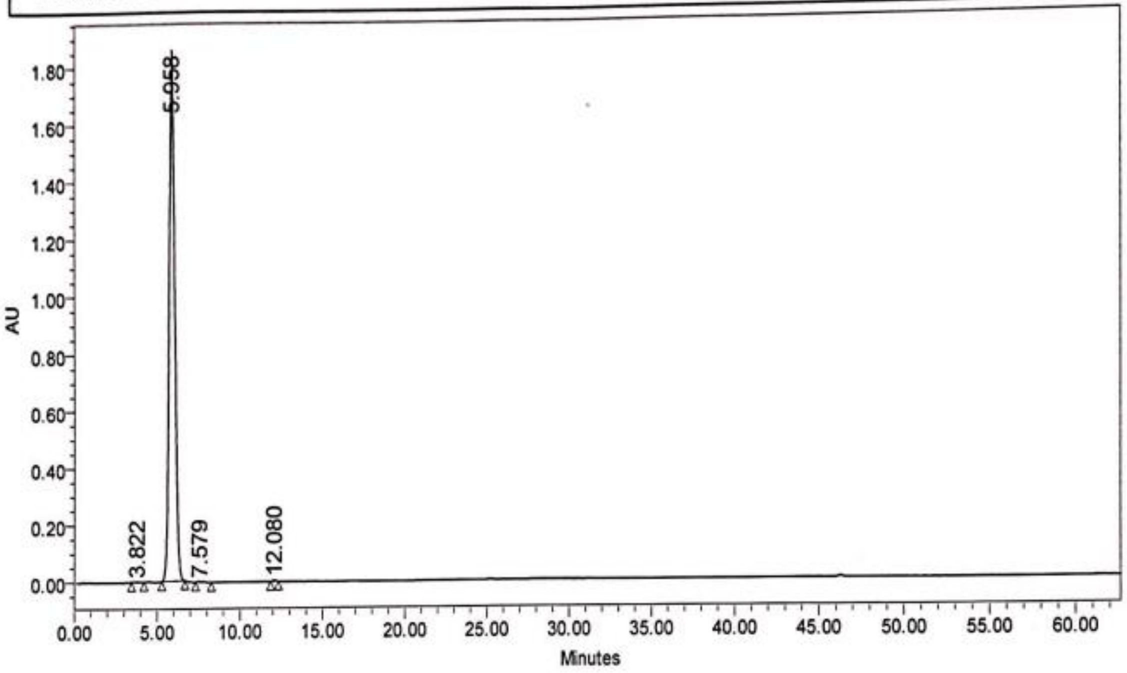

	RT	Area	% Area	Height
1	3.822	37043	0.09	1483
2	5.958	42658541	99.61	1859139
3	7.579	62952	0.15	2208
4	12.080	68946	0.16	4573

Figure S79 HPLC spectrum of **3i**

SAMPLE INFORMATION

Sample Name:	JAA17P1	Acquired By:	natcri
Sample Type:	Unknown	Sample Set Name:	JAA17P01
Vial:	1	Acq. Method Set:	50_100_60m_100_20m_80m_Ana
Injection #:	1	Processing Method:	JAA17P1 07042022
Injection Volume:	10.00 ul	Channel Name:	254.0nm
Run Time:	80.0 Minutes	Proc. Chnl. Descr.:	PDA 254.0 nm

Date Acquired: 07/04/2022 11:24:58 AM ICT
Date Processed: 07/04/2022 12:28:41 PM ICT

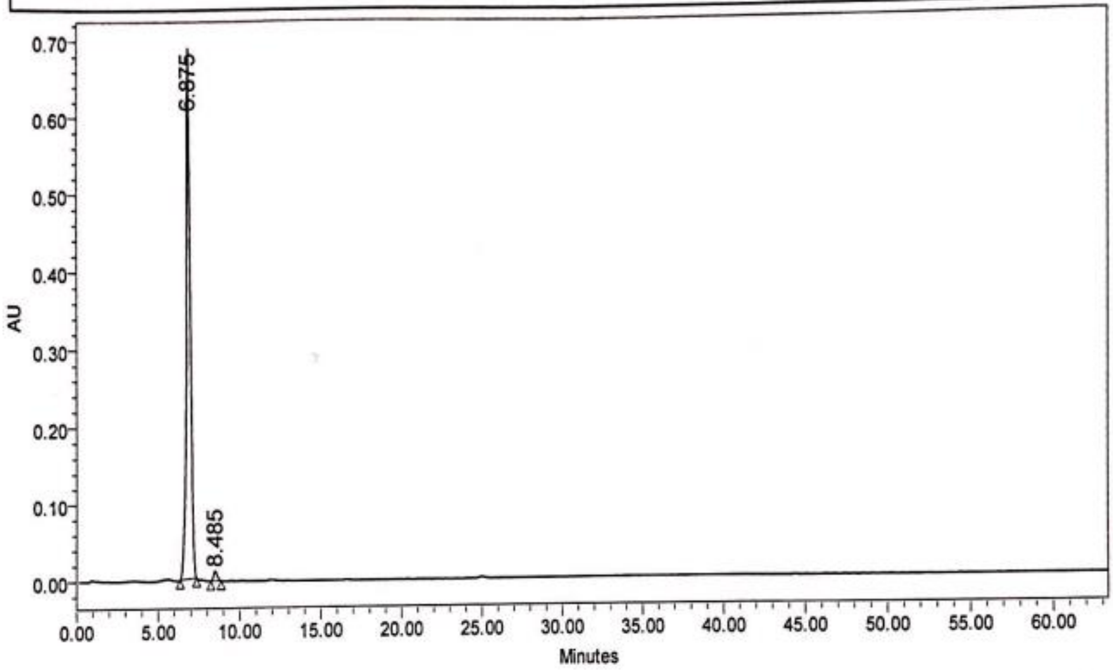

	RT	Area	% Area	Height
1	6.875	11461717	98.25	689256
2	8.485	203585	1.75	12902

Figure S80 HPLC spectrum of **3j**

SAMPLE INFORMATION

Sample Name:	JAA18P31	Acquired By:	natcri
Sample Type:	Unknown	Sample Set Name:	JAA07P1
Vial:	7	Acq. Method Set:	50_100_60m_100_20m_80m_Ana
Injection #:	1	Processing Method:	JAA18P3 010032022
Injection Volume:	10.00 ul	Channel Name:	254.0nm
Run Time:	80.0 Minutes	Proc. Chnl. Descr.:	PDA 254.0 nm

Date Acquired: 10/03/2022 5:55:10 PM ICT
Date Processed: 10/03/2022 6:56:51 PM ICT

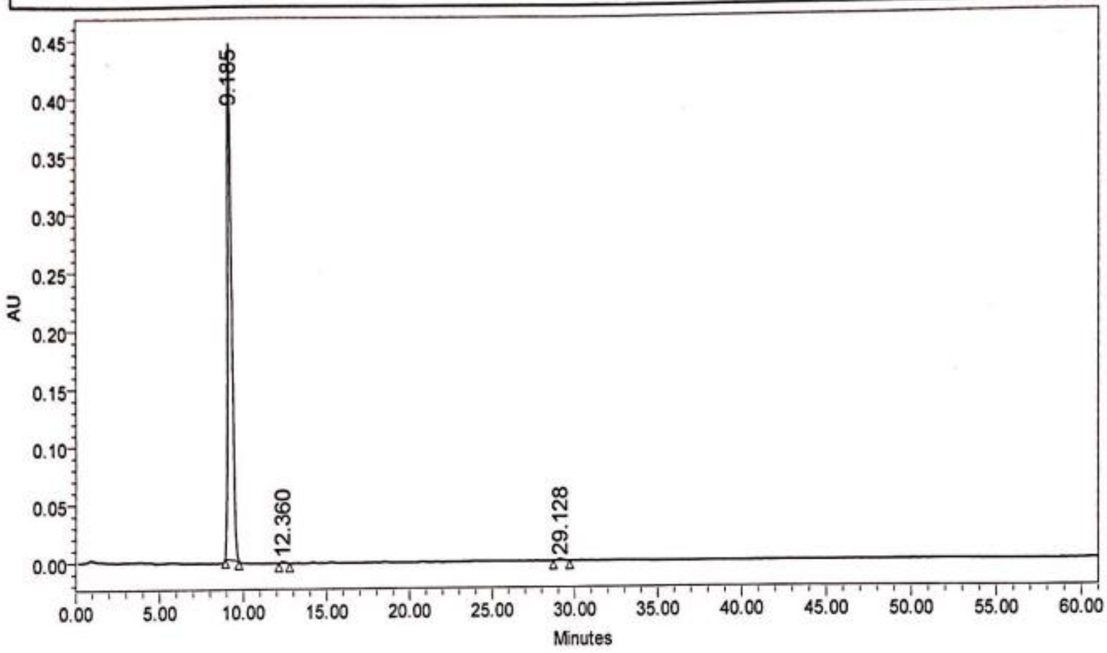

	RT	Area	% Area	Height
1	9.185	7342453	99.19	443680
2	12.360	27358	0.37	1506
3	29.128	32845	0.44	1379

Figure S81 HPLC spectrum of **3k**

SAMPLE INFORMATION

Sample Name:	JAA19P1	Acquired By:	natcri
Sample Type:	Unknown	Sample Set Name:	JAA07P1
Vial:	8	Acq. Method Set:	50_100_60m_100_20m_80m_Ana
Injection #:	1	Processing Method:	JAA19P1 10032022
Injection Volume:	10.00 ul	Channel Name:	254.0nm
Run Time:	80.0 Minutes	Proc. Chnl. Descr.:	PDA 254.0 nm

Date Acquired: 10/03/2022 7:17:37 PM ICT
Date Processed: 10/03/2022 7:59:47 PM ICT

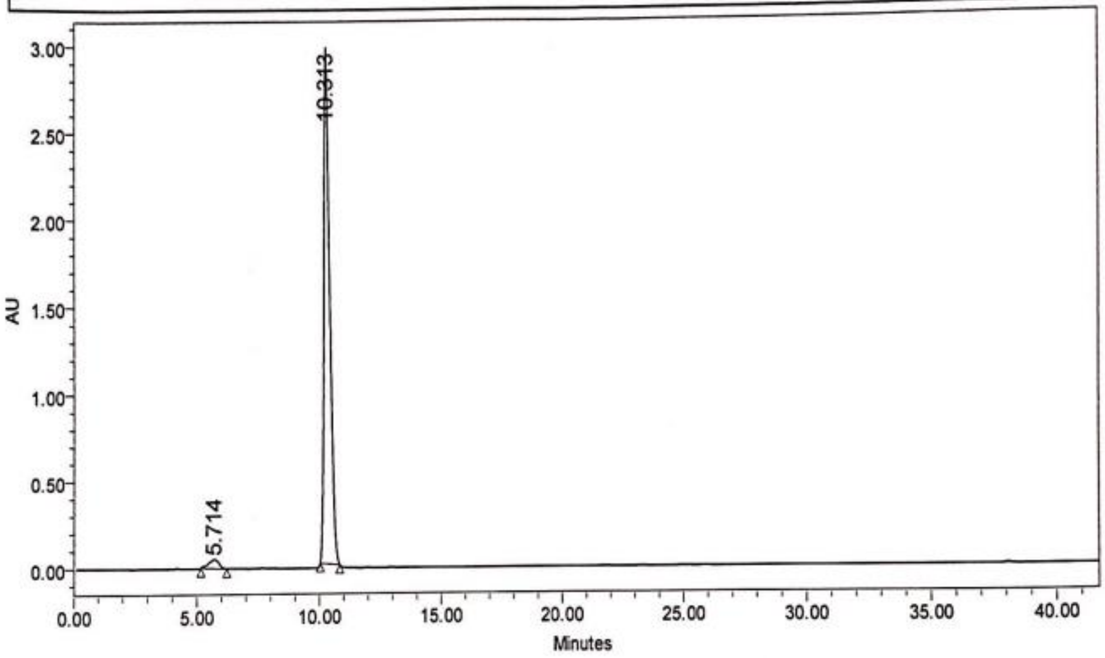

	RT	Area	% Area	Height
1	5.714	1488059	2.99	51651
2	10.313	48313552	97.01	2960463

Figure S82 HPLC spectrum of **3l**

SAMPLE INFORMATION

Sample Name:	JAA24P1	Acquired By:	natcri
Sample Type:	Unknown	Sample Set Name:	JAA20P1
Vial:	4	Acq. Method Set:	50_100_60m_100_20m_80m_Ana
Injection #:	1 20	Processing Method:	JAA24P1 1 11032022
Injection Volume:	10.00 ul	Channel Name:	254.0nm
Run Time:	80.0 Minutes	Proc. Chnl. Descr.:	PDA 254.0 nm

(2)

Date Acquired: 11/03/2022 12:50:17 PM ICT
Date Processed: 11/03/2022 1:53:30 PM ICT

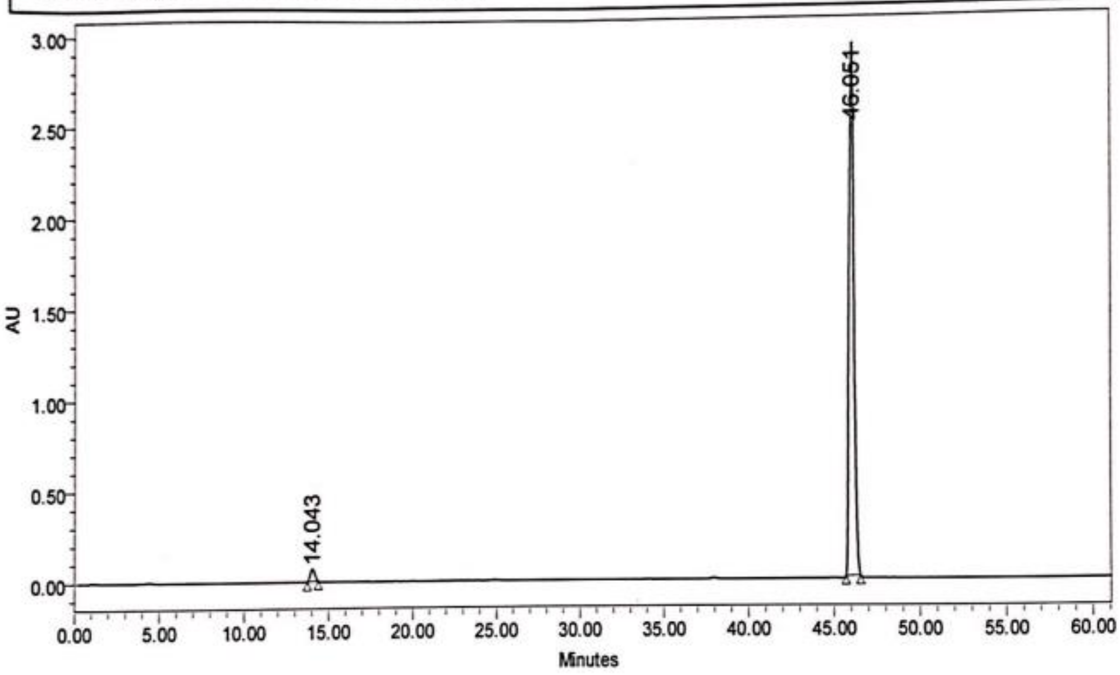

	RT	Area	% Area	Height
1	14.043	1273400	2.20	68054
2	46.051	56614494	97.80	2919734

Figure S83 HPLC spectrum of **3m**

SAMPLE INFORMATION

Sample Name:	JAA41P1	Acquired By:	natcri
Sample Type:	Unknown	Sample Set Name:	JAA40P1
Vial:	7	Acq. Method Set:	50_100_60m_100_20m_80m_Ana
Injection #:	1	Processing Method:	JAA44P1 14032022
Injection Volume:	10.00 ul	Channel Name:	254.0nm
Run Time:	80.0 Minutes	Proc. Chnl. Descr.:	PDA 254.0 nm
Date Acquired:	14/03/2022 5:37:13 PM ICT		
Date Processed:	14/03/2022 6:40:01 PM ICT		

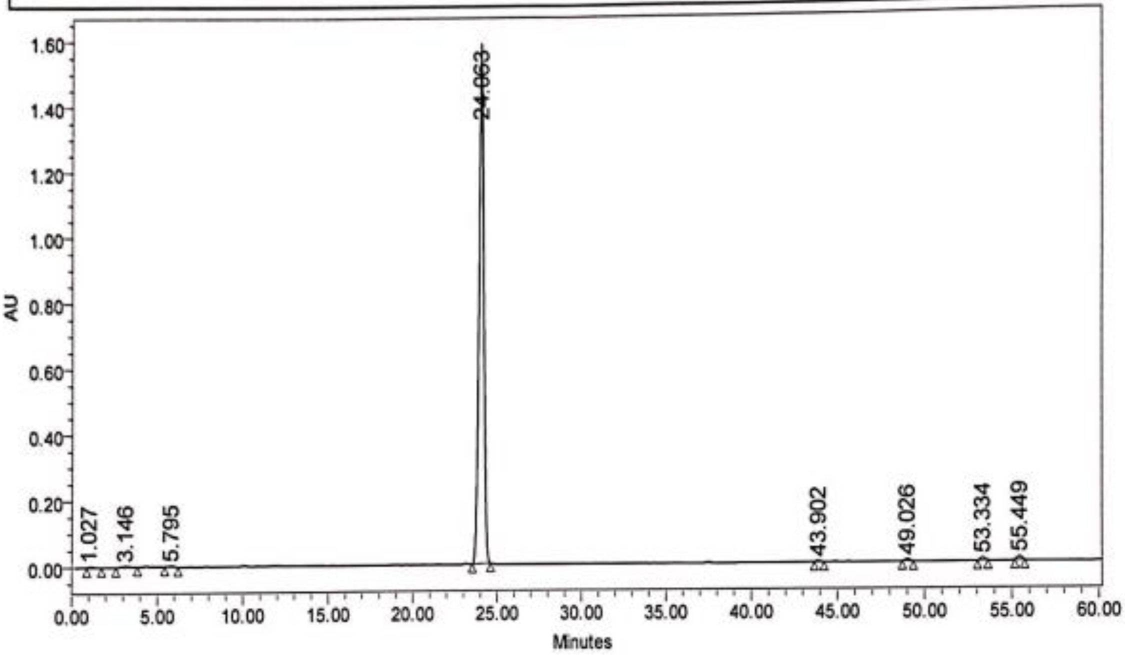

	RT	Area	% Area	Height
1	1.027	60731	0.19	2571
2	3.146	102522	0.32	3175
3	5.795	77877	0.25	3214
4	24.063	30856279	97.53	1586555
5	43.902	72551	0.23	4267
6	49.026	125617	0.40	6758
7	53.334	147054	0.46	8213
8	55.449	196620	0.62	11560

Figure S84 HPLC spectrum of **3n**

SAMPLE INFORMATION

Sample Name:	JA40P2	Acquired By:	natcri
Sample Type:	Unknow n	Sample Set Name:	JAA40P2_03062022
Vial:	2	Acq. Method Set:	50_100_60m_100_20m_80m_Ana
Injection #:	1	Processing Method:	JAA40P2 2 13032022
Injection Volume:	10.00 ul	Channel Name:	254.0nm
Run Time:	80.0 Minutes	Proc. Chnl. Descr.:	PDA 254.0 nm
Date Acquired:	03/06/2022 11:55:50 AM ICT		
Date Processed:	03/06/2022 1:06:33 PM ICT		

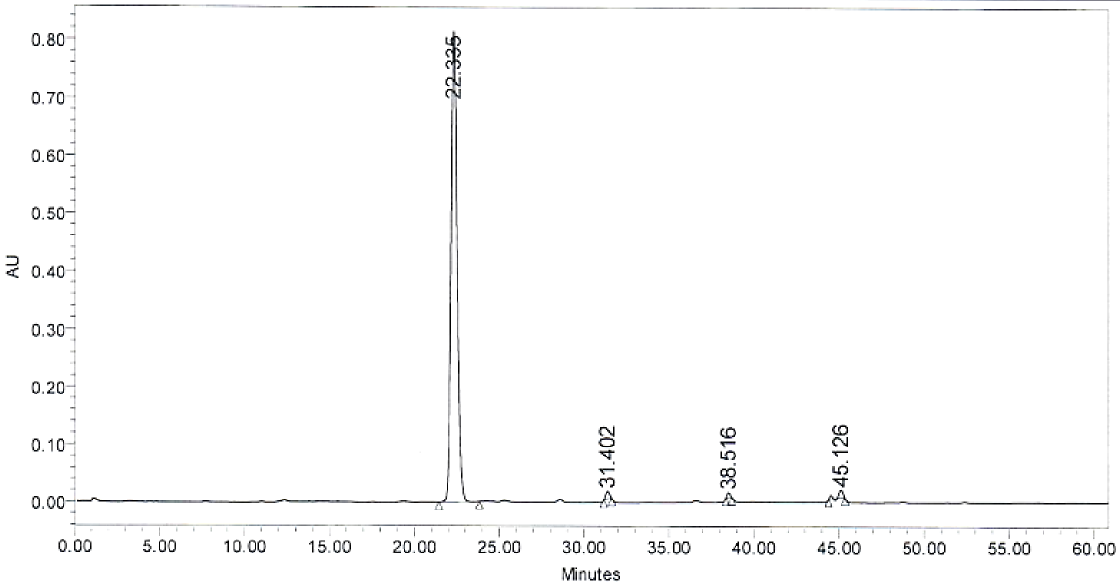

	RT	Area	% Area	Height
1	22.335	20044015	97.00	814330
2	31.402	211681	1.02	13057
3	38.516	98738	0.48	8129
4	45.126	308779	1.49	13576

Figure S85 HPLC spectrum of **4a**

SAMPLE INFORMATION

Sample Name:	JAA73P2	Acquired By:	natcri
Sample Type:	Unknown	Sample Set Name:	JAA763P2
Vial:	2	Acq. Method Set:	50_100_60m_100_20m_80m_Ana
Injection #:	1	Processing Method:	JAA73P2 18032022
Injection Volume:	10.00 ul	Channel Name:	254.0nm
Run Time:	80.0 Minutes	Proc. Chnl. Descr.:	PDA 254.0 nm
Date Acquired:	15/03/2022 2:24:43 PM ICT		(3)
Date Processed:	18/03/2022 8:52:51 AM ICT		

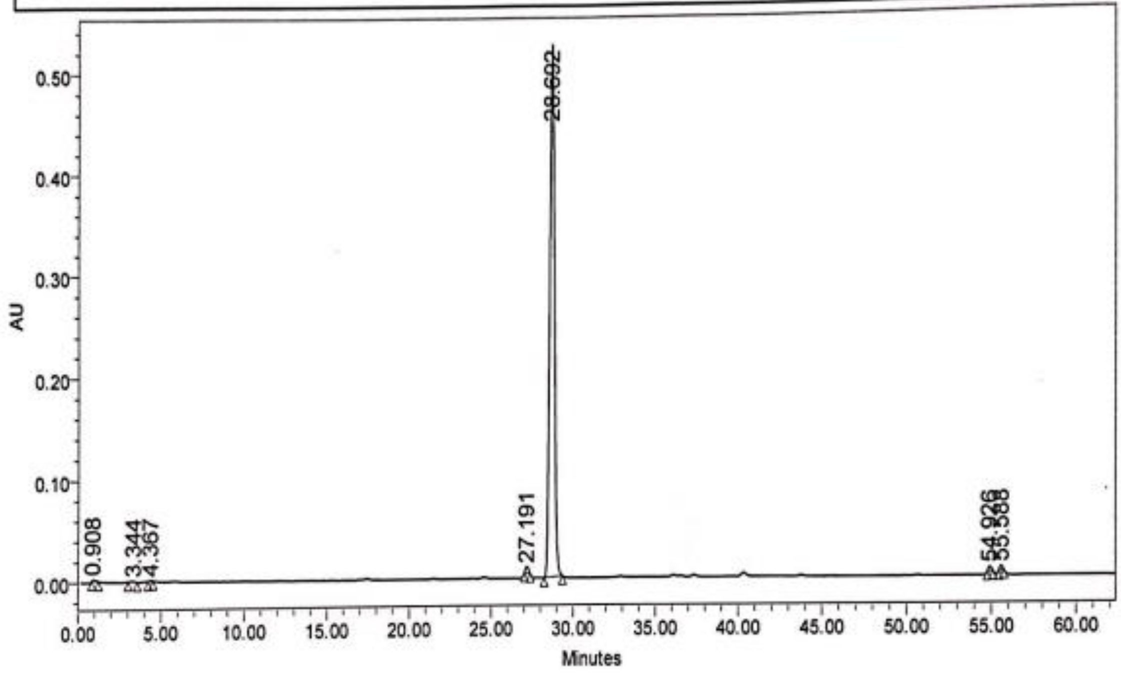

	RT	Area	% Area	Height
1	0.908	21877	0.21	1759
2	3.344	16567	0.16	843
3	4.367	8496	0.08	803
4	27.191	80536	0.76	6280
5	28.692	10355529	97.78	527186
6	54.926	54724	0.52	4453
7	55.588	52704	0.50	4529

Figure S86 HPLC spectrum of **4b**

SAMPLE INFORMATION

Sample Name:	JAA36P1	Acquired By:	natcri
Sample Type:	Unknown	Sample Set Name:	JAA12P1
Vial:	6	Acq. Method Set:	50_100_60m_100_20m_80m_Ana
Injection #:	1	Processing Method:	JAA36P1 13032022
Injection Volume:	10.00 ul	Channel Name:	254.0nm
Run Time:	80.0 Minutes	Proc. Chnl. Descr.:	PDA 254.0 nm

Date Acquired: 13/03/2022 4:42:49 PM ICT
Date Processed: 13/03/2022 5:43:56 PM ICT

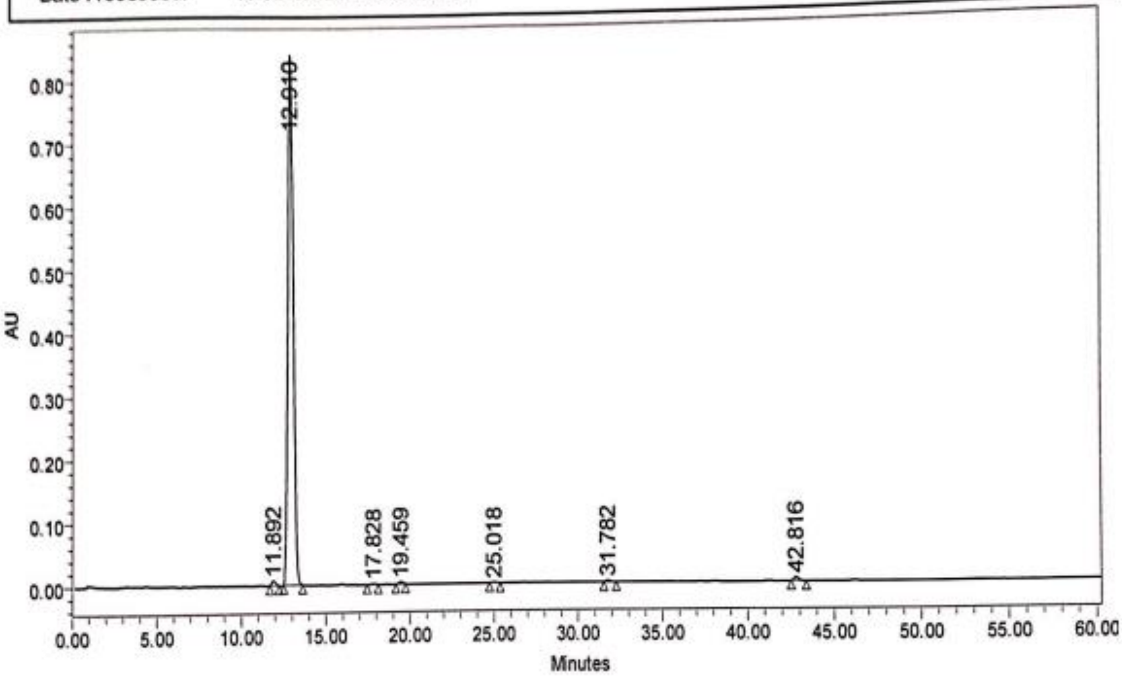

	RT	Area	% Area	Height
1	11.892	114533	0.61	7252
2	12.910	18464747	98.02	840545
3	17.828	27835	0.15	1618
4	19.459	59910	0.32	3404
5	25.018	24123	0.13	1230
6	31.782	41047	0.22	2329
7	42.816	105405	0.56	5960

Figure S87 HPLC spectrum of **5**

SAMPLE INFORMATION

Sample Name:	JAA34P1	Acquired By:	natcri
Sample Type:	Unknow n	Sample Set Name:	JAA12P1
Vial:	5	Acq. Method Set:	50_100_60m_100_20m_80m_Ana
Injection #:	1	Processing Method:	JAA34P1 2 13032022
Injection Volume:	10.00 ul	Channel Name:	254.0nm
Run Time:	80.0 Minutes	Proc. Chnl. Descr.:	PDA 254.0 nm

Date Acquired: 13/03/2022 3:18:03 PM ICT
Date Processed: 13/03/2022 4:22:14 PM ICT

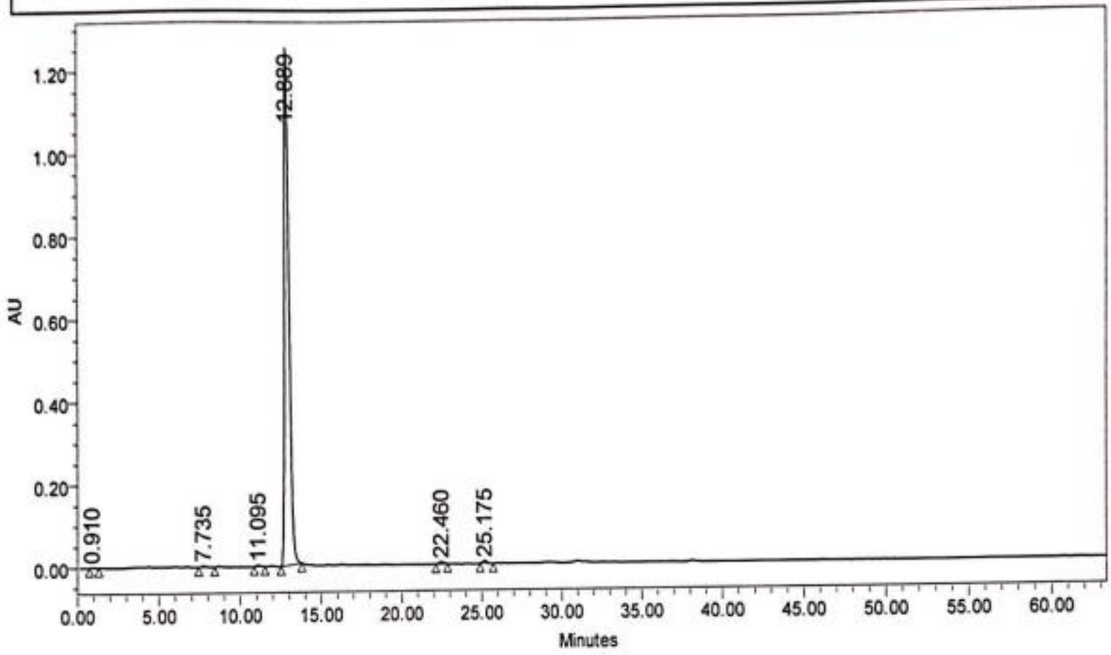

	RT	Area	% Area	Height
1	0.910	18181	0.07	1516
2	7.735	66484	0.27	4289
3	11.095	63099	0.25	3257
4	12.889	24629860	98.42	1254186
5	22.460	116689	0.47	5519
6	25.175	131545	0.53	6856

Figure S88 HPLC spectrum of **6**

7. References

[i] van Amsterdam, F. T. M., Roveri, A., Maiorino, M., Ratti, E. & Ursini, F. Lacidipine: A Dihydropyridine Calcium Antagonist with Antioxidant Activity. *Free Radic. Biol. Med.* **12,** 183–187. https://doi.org/10.1016/0891-5849(92)90025-c (1992).

[ii] Gerhäuser, C. *el at*. Mechanism-based *in vitro* Screening of Potential Cancer Chemopreventive Agents. *Mutat. Res.* **523–524**, 163–172. https://doi.org/10.1016/S0027-5107(02)00332-9 (2003).

[iii] Rangkadilok, N. *el at*. Evaluation of Free Radical Scavenging and Antityrosinase Activities of Standardized Longan Fruit Extract. *Food Chem. Toxicol.* **45**, 328–336. https://doi.org/10.1016/j.fct.2006.08.022 (2007).

[iv] Gleason, M.M., Rojas, C.J., Learn, K.S., Perrone, M.H. & Bilder, G.E. Characterization and Inhibition of 15-lipoxygenase in Human Monocytes: Comparison with Soybean 15-lipoxygenase. *Am. J. Physiol.* **268**, C1301–C1307. https://doi.org/10.1152/ajpcell.1995.268.5.c1301 (1995).
